# In silico identification of two peptides with antibacterial activity against multidrug-resistant *Staphylococcus aureus*

**DOI:** 10.1038/s41522-022-00320-0

**Published:** 2022-07-14

**Authors:** Linda B. Oyama, Hamza Olleik, Ana Carolina Nery Teixeira, Matheus M. Guidini, James A. Pickup, Brandon Yeo Pei Hui, Nicolas Vidal, Alan R. Cookson, Hannah Vallin, Toby Wilkinson, Denise M. S. Bazzolli, Jennifer Richards, Mandy Wootton, Ralf Mikut, Kai Hilpert, Marc Maresca, Josette Perrier, Matthias Hess, Hilario C. Mantovani, Narcis Fernandez-Fuentes, Christopher J. Creevey, Sharon A. Huws

**Affiliations:** 1 Institute for Global Food Security, School of Biological Sciences, Queen’s University Belfast, 19 Chlorine Gardens, Belfast, Northern Ireland BT9 5DL UK; 2 CNRS Enzyme and Cell Engineering Laboratory, Université de Technologie de Compiègne, Sorbonne Universités, Rue du Docteur Schweitzer, CS 60319, CEDEX, 60203 Compiègne, France; 3 Departamento de Microbiologia, Universidade Federal de Viçosa, Viçosa, 36570-900 Brasil; 4 University College Fairview (UCF), 4178, Jalan 1/27D, Section 6, Wangsa Maju, 53300 Kuala Lumpur, Malaysia; 5 Yelen Analytics, Aix-Marseille University ICR, 13013 Marseille, France; 6 Institute of Biological Environmental and Rural Sciences, Aberystwyth University, Aberystwyth, Wales SY23 3DA UK; 7 The Roslin Institute and R(D)SVS, University of Edinburgh, Edinburgh, United Kingdom; 8 Specialist Antimicrobial Chemotherapy Unit, Public Health Wales, University Hospital of Wales, Heath Park, Cardiff, CF14 4XW UK; 9 Karlsruhe Institute of Technology, Institute for Automation and Applied Informatics, Hermann-von-Helmholtz-Platz 1, 76344 Eggenstein, Leopoldshafen Germany; 10 Institute of Infection and Immunity, St George’s, University of London, Cranmer Terrace, London, SW17 0RE UK; 11 Aix Marseille University, CNRS, Centrale Marseille, iSm2, Marseille, France; 12 UC Davis, College of Agricultural and Environmental Sciences, California, 95616 CA USA

**Keywords:** Antimicrobials, Metagenomics, Microbiome, Biofilms, Cellular microbiology

## Abstract

Here we report two antimicrobial peptides (AMPs), HG2 and HG4 identified from a rumen microbiome metagenomic dataset, with activity against multidrug-resistant (MDR) bacteria, especially methicillin-resistant *Staphylococcus aureus* (MRSA) strains, a major hospital and community-acquired pathogen. We employed the classifier model design to analyse, visualise, and interpret AMP activities. This approach allowed in silico discrimination of promising lead AMP candidates for experimental evaluation. The lead AMPs, HG2 and HG4, are fast-acting and show anti-biofilm and anti-inflammatory activities in vitro and demonstrated little toxicity to human primary cell lines. The peptides were effective in vivo within a *Galleria mellonella* model of MRSA USA300 infection. In terms of mechanism of action, HG2 and HG4 appear to interact with the cytoplasmic membrane of target cells and may inhibit other cellular processes, whilst preferentially binding to bacterial lipids over human cell lipids. Therefore, these AMPs may offer additional therapeutic templates for MDR bacterial infections.

## Introduction

The decline in effective treatment strategies for multidrug-resistant (MDR) bacterial infections, caused mainly by antibacterial resistance, threatens our ability to treat infections and calls for alternative treatment strategies^1^. The MDR Gram-positive bacteria, methicillin-resistant *Staphylococcus aureus* (MRSA), a human opportunistic pathogen, has become a leading causative agent of hospital and community-acquired infections over the past few decades, posing a number of challenges for physicians^2,3^. Due to its pathogenicity and potential impact on a large population, the World Health Organisation (WHO) has classified MRSA and vancomycin-intermediate and resistant *S. aureus* as one of its priority pathogens^4^. Concomitantly, the Centre for Disease Control and Prevention (CDC), states that MRSA represents a major burden on healthcare as it can acquire resistance to almost any class of antibiotic^5^, leading to more than 80,000 invasive infections and 11,000 deaths each year in the United States alone^6^. In 2017, the prevalence of MRSA infections in England and Northern Ireland increased for the first time since 2011^7^. Moreover, treatment of MRSA bacteraemia is a long-standing challenge for the healthcare profession, often complicated by metastatic infections, treatment failure and mortality^3^. Therefore, antimicrobial compounds with new modes of action that are effective for the treatment of MRSA infections are urgently needed.

The research focussed on identifying and optimising the use of antimicrobial peptides (AMPs) in infectious disease treatment has recently intensified as they have shown great promise as a new class of therapeutic agents^1^. AMPs have a broad spectrum of activity including against bacteria, fungi, viruses and parasites, form amphipathic structures, which aid interaction with the cell membrane, and have a multimodal mechanism of action, which contributes to the delayed onset of resistance in the target pathogen^8^. Recent research have identified novel AMPs from a variety of microbiomes from the human to nematode gastrointestinal tract microbiomes, emphasising the potential to harvest microbiome-based chemicals to treat MDR bacteria^9–11^. Indeed, the rumen microbiome has also been shown to be a resource for novel AMP discovery^12–15^ and urgently needed alternative therapeutics to tackle multidrug-resistant bacterial infections.

Advances in nucleic acid-based technology (i.e. second-generation sequencing, meta omic) and high-throughput sequence analytic methods (e.g. advanced bioinformatic approaches) have created new opportunities to investigate the complex relationships and niches within microbial communities, redefining our understanding and improving our ability to describe various microbiomes, including the rumen microbiome. An enhanced understanding of microbiomes facilitates the identification and utilisation of some of the beneficial traits that are conveyed by these microbiomes^16^. Several metagenomic datasets from the rumen have been generated in the last few years, illustrating some of the beneficial traits of the rumen microbiome including the presence of large numbers of novel glycosyl hydrolases^17^, esterases^18^, lipolytic enzymes^18^ and more recently, antimicrobial compounds^14,16^, possessing therapeutic potential for the treatment of MDR bacteria.

Here, we combined the application of metagenomics, using one of the largest rumen metagenomic dataset^17^ available, with advanced computational analytic tools and chemical models to identify and characterise AMP candidates for the treatment of MDR infections. This metagenomic dataset contains more than 268 Gb, or 1.5 billion read pairs, of metagenomic DNA from microorganisms that colonised plant fibre during incubation in the rumen of fistulated cows. De novo assembly of reads resulted in more than 2.5 million predicted open reading frames at an average of 542 bp and 55% predicted full-length genes. We employed the classifier model design, a feature extraction method using molecular descriptors for amino acids for the analysis, visualisation, and interpretation of AMP activities, and the in silico discrimination of active and inactive peptides in order to define a small number of promising new lead AMP candidates for chemical synthesis and experimental evaluation. We also show the innocuity ex vivo, and the anti-MRSA efficacy of two of these AMPs both in vitro and in vivo.

## Results

### In silico identification of AMPs using computational analysis

**Fig. 1:**
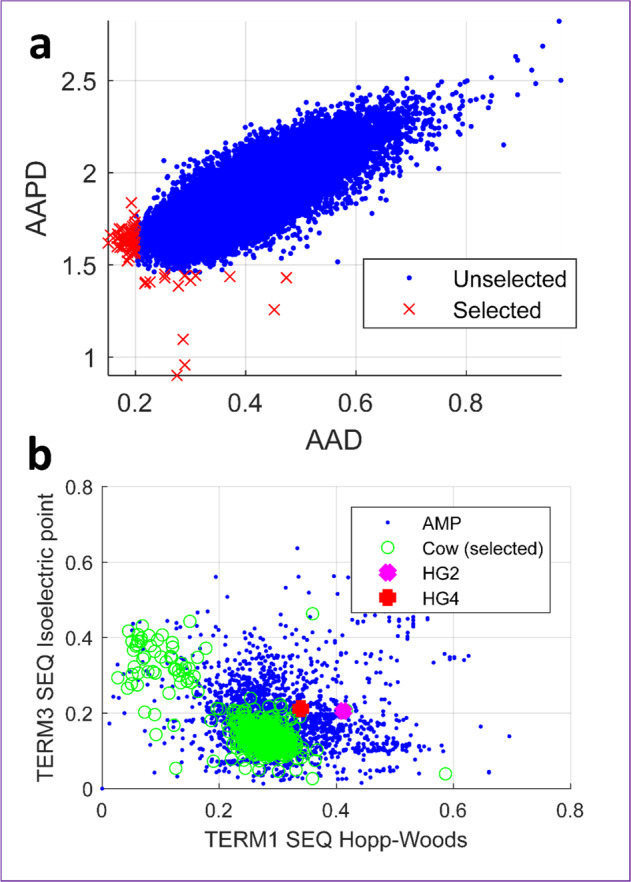
In silico identification of peptides. **a** Visualisation of distances for AA acids (AAD) and AA pairs (AAPD) for the first 68,274 sequences from library “Cow”^17^ meeting the first selection criteria: candidates with AAD <0.2 or AAPD <1.45 are selected as candidates (here: 65). **b** standard hydrophobicity (TERM1 SEQ Hopp-Woods)—loading (positively charged, TERM3 SEQ Isoelectric Point) plot. Blue dots are known AMPs (library ‘AMP’ consisting of AMPs from the APD2^45^ and Hilpert Library^46^), green coloured signs are AMP hits identified from library ‘Cow’, and finally selected peptides HG2 (magenta) and HG4 (red).

After applying the first selection criteria (i.e. protein sequences with a maximum length of 200 amino acids (AAs) and not more than 5% unknown AAs (marked by X, *)^19^, the computational analysis identified 917,636 sequences (36%) of the 2,547,270 predicted protein sequences in the Hess et al. dataset (termed Library ‘Cow’). Of these 917,636 sequences, only 829 sequences fulfilled the criteria of AA distances (AAD) <0.2 or small AA pair distances (AAPD) <1.45, ensuring high possibility of these sequences being AMPs^20,21^. For example, only 65 sequences met these AAD and AAPD criteria in the first 68,274 sequences analysed, with isolated points outside a relatively dense distribution area as illustrated in Fig. 1a. Descriptor computations generated positively charged loading—hydrophobicity plots, indicating that the selected sequences from the Library ‘Cow’ are represented in only a small portion of all AMP regions from the Library ‘AMP’ (see Fig. 1b). Results from each computational step used in the identification of potentially novel AMPs from the Hess et al. rumen metagenomic dataset^17^ is summarised in Supplementary Table 1.

Six promising AMP sequences (Hess-Gene 1–6 (HG1- HG6) (Supplementary Table 2) were identified from the 829 sequences as holding the greatest potential for being active AMPs. The corresponding nucleotide sequence and additional information for the identified genes can be found in Supplementary Table 3. Two of these six promising candidates, HG2 (MKKLLLILFCLALALAGCKKAP) and HG4 (VLGLALIVGGALLIKKKQAKS), the shortest length AMPs containing 22 and 21 AA residues respectively, were selected for subsequent characterisation. Sequence homology analysis using NCBI’s BLASTP^22^ against the non-redundant (nr) protein sequences (see Table 1) suggests that HG2 has similarity (71%) to an ABC transporter substrate-binding protein from *Megasphaera elsdenii* and many uncharacterised proteins, while HG4 has similarity to a hypothetical protein from *Megasphaera elsdenii* (77%). It is important to note that the AMPs, HG2 and HG4 match only a small portion of their homologous sequences (see Table 1), which are automatically curated unreviewed sequences, and this may indicate the novelty of the identified AMPs from the rumen metagenomic dataset. In addition, eggNOG mapper^23^ annotations revealed that the HG2 and HG4 bearing sequences map to different membrane-associated proteins including transport and receptor proteins, amongst other functions (see Supplementary Table 4).

**Table 1:** Homology of antimicrobial peptides HG2 and HG4 to known sequences.

Assigned AMP name	HG2	HG4	
Sequence	MKKLLLILFCLALALAGCKKAP*	VLGLALIVGGALLIKKKQAKS*	
Amino acid (AA) (length)	22	22	
Location on cow dataset	NODE_664976_length_19740_cov_2.033485_orf_00810 19724..19789	NODE_3958153_length_85376_cov_8.525382_orf_203250 82784..82849	
Most similar homologue on APD3 (stop codon ‘*’ removed)	APD ID	AP02395	AP01737
	Similarity %	41.67%	48%
Most similar homologue on NCBI blastp (stop codon ‘*’ removed)	Accession number	MBM6702036.1	WP_226888091.1
	Description	ABC transporter substrate-binding protein [*Megasphaera elsdenii*]	Hypothetical protein [*Megasphaera elsdenii*]
	Score bits/Identities %/E-value	39.2 bits (85)/ 17/24(71%) / 1e-05	27.4 bits (57)/ 10/13(77%)/ 0.13

HG2 and HG4 were chemically synthesised as C-terminal amidated peptides on resin (≥95% purity, see Supplementary Fig. 1 for mass spectrometry analysis and peptide synthesis reports) using solid-phase Fmoc peptide chemistry^24^. HG2 was synthesised with a disulphide bond linking cysteine residues at positions 10 and 18 since HG2 lacking this disulphide bond showed no antimicrobial activity (ie. MICs greater than the highest concentration tested (>1024 µg/mL) for all bacterial strains included in the initial screen (see the antimicrobial activity of linear HG2 in Supplementary Table 5). Similar to previously reported antimicrobial peptides^25^, HG2 (C_111_H_196_N_26_O_23_S_3_; MW = 2359.12 Da) and HG4 (C_99_H_182_N_26_O_24_; MW = 2120.69 Da) are cationic, both having a net positive charge of +4. A hydrophobicity ratio of 57% was calculated for HG4 using ExPASy’s ProtParam tool^26^, while HG2 was predicted to possess a hydrophobicity ratio of 72%, which is high compared to the ratio that has been reported for most AMPs^27,28^. This puts HG2 into the small group of AMPs, representing <1% of AMPs deposited in the APD3 database for which a hydrophobicity ratio of ≥72% has been reported^19^. The positive charge and hydrophobicity of AMPs are known to contribute to their antimicrobial activity as they play a role in their ability to interact with the bacterial cell membrane^29^.

### Three-dimensional modelling of peptide structures

**Fig. 2:**
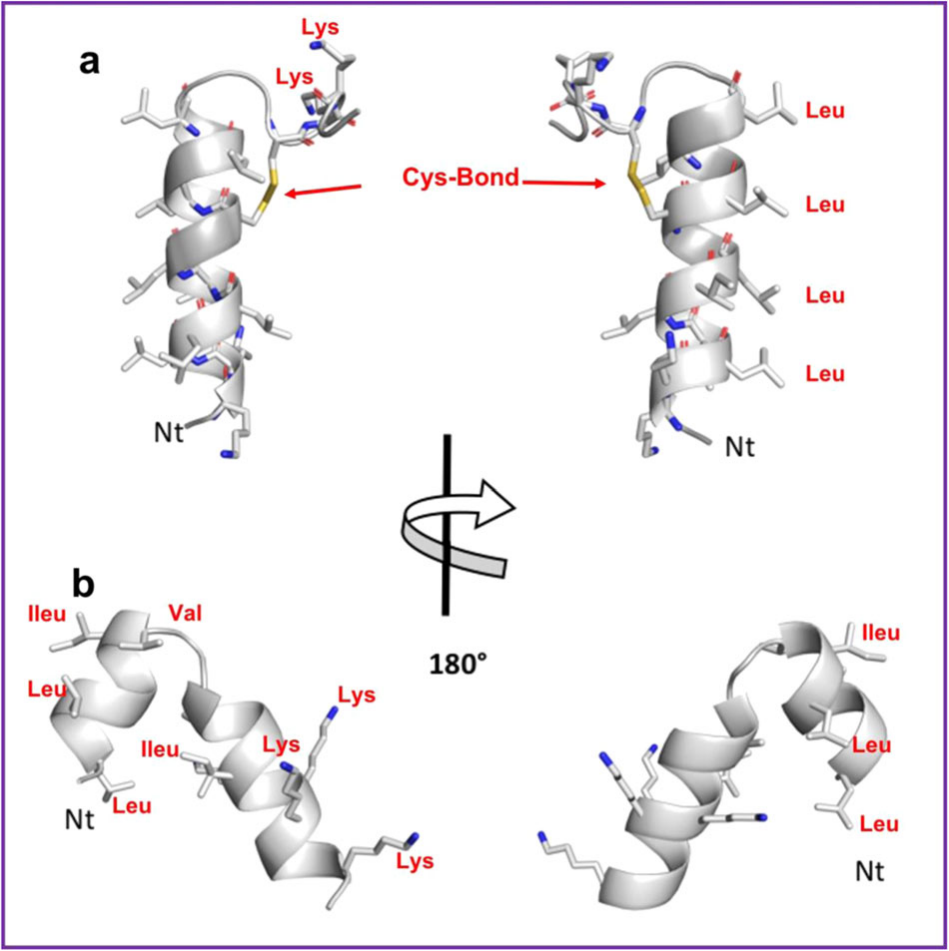
Predicted 3D structures for peptides. Molecular 3D structures for **a** HG2, **b** HG4. Main-chain and side chains are depicted in ribbon and stick representation respectively and coloured according to atom type: Carbon, oxygen and nitrogen in green, red and blue respectively. Two orientations are shown rotated about the shown axis. Ct and Nt as well as selected residues are depicted in the figure. Figures were rendered using PyMol.

Three-dimensional structural modelling of HG2 and HG4 suggests that these peptides have a high proportion of helical content (Fig. 2). In the case of HG2 (Fig. 2a), the cysteine bond stabilises the capping of the helix and the C-terminus region. Noteworthy is the clear amphipathic nature of the helix with the hydrophobic residues, particularly, Leu, aligned in a typical Leu-zipper motif. The N- and C-termini include a high proportion of the positively charged residues (Lys), which contribute to the segregation of charges along the peptide. As shown previously, the amphipathicity of peptides, in particular, with helical conformation, is an important feature of antimicrobial peptides that explains their ability to interact with bacterial membranes^29^.

Modelling also showed a high content of helical conformation in HG4 (Fig. 2b). HG4 forms a helix-turn-helix motif with the C-terminal helix capping stabilised by hydrophobic interactions between the helices. The distribution of charges is asymmetrical as expected, given the sequence of the peptide with the C-terminal part including all charged residues (Lys mainly). The N-terminal helix contains mainly hydrophobic residues and the C-terminal helix all charged residues with the exception of the first turn of the helix, containing a high proportion of hydrophobic residues that form a mini core with the previous helix possibly stabilising the conformation of the motif. The resulting conformation of the peptide is therefore an amphipathic molecule, albeit different from HG2, which could also point to a mechanism of action on membranes.

### Antimicrobial susceptibility studies

#### Determination of minimum inhibitory concentrations (MIC)

We determined the antibacterial activity of HG2 and HG4 against various pathogens including strains of *Acinetobacter baumannii*, *Klebsiella pneumoniae, Pseudomonas aeruginosa, Escherichia coli*, *Salmonella enterica* serovar Typhimurium, *Staphylococcus aureus, Bacillus cereus, Enterococcus faecalis* and *Listeria monocytogenes* and some clinically important multidrug-resistant (MDR) strains (Table 2). HG2 and HG4 had antibacterial activity mostly against Gram-positive pathogens, and were most potent against methicillin-resistant Staphylococcus aureus (MRSA) strains (Table 2). HG2 had a MIC range of 16–32 µg/ml, while HG4’s MIC was 32–64 µg/ml, depending on the MRSA strain and falling within the range of MICs for other rumen-derived AMPs identified previously^14^ as well as those of AMPs identified by rational design, e.g. DP7 amongst others^30^. The peptides also showed activity against some Gram-negative bacteria strains, specifically some non-antibiotic resistant *A. baumannii* strains, *P. aeruginosa* strains C3719 and LES400 isolated from cystic fibrosis patients (Table 2).

**Table 2:** MDR bacteria susceptibility to HG2 and HG4 and comparator antibiotics measured by MIC.

Organisms			MIC for peptides and comparator antibiotics (*µ*g/ml)					
Lab no./Strain ID	Organism	Resistances	Cip/Levof (L)	Polymyxin B	HG2	HG4	Vancomycin	Mupirocin
EMRSA-15	*S. aureus*	MRSA, Cip	>256, 1(L)	256	32	32	2	—
ATCC 33591		MRSA	0.015 (L)	—	64	32	2	—
USA300 BAA-1717		MRSA	0.0075 (L)	—	16	32	2	0.12
RN4220		Sensitive	>256	256	256	32	1	—
JH2-2	*Ent. faecalis*		64	32	256	128	64	—
NCTC 11994	*L. monocytogenes*		64	64	—	512	64	—
518842	*K. pneumoniae*	CTX-M	>128	2	512	512	—	—
ATCC 700603		SHV-18	0.25	2	>512	>512	—	—
NCTC 13442		OXA-48	64	8	512	512	—	—
526903		Sensitive	0.03	4	>512	512	—	—
	*A. baumannii*	IMI, MER	16	0.25	128	64	—	—
515785		OXA-23, OXA-50	>128	0.5	256	128	—	—
515908		Sensitive	16	0.5	32	64	—	—
515722		Sensitive	16	0.25	16	32	—	—
K12	*E. coli*		0.06	2	256	512	128	—
SL1344	*Sal. typhimurium*		0.12	2	256	512	256	—
	*B. cereus*		0.015	—	256	512	—	—
PA01	*P. aeruginosa*		0.5	2	>512	>512	64	—
AMT0060	*P. aeruginosa (CF)*		0.12	0.5	256	256	—	—
C3719			4	1	64	128	—	—
LES400			4	1	64	128	—	—

*Cip* ciprofloxacin, *Lev* (*L*) levofloxacin, *CF* isolates from cystic fibrosis infections, *OXA* oxacillin, *CTX-M* extended-spectrum β-lactamase, SHV-18 β- lactamase, *IMI* imipenem, *MER* meropenem.

#### Time-kill kinetics

**Fig. 3:**
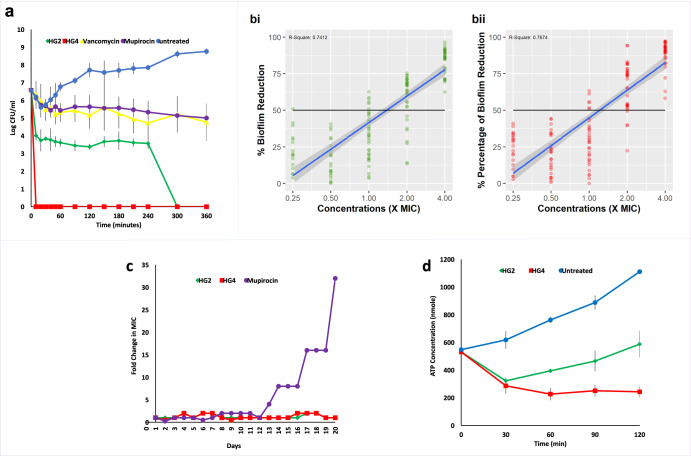
Antimicrobial susceptibility and activity of HG2 and HG4. **a** Time-dependent kill of MRSA USA300 cells by AMPs and comparator antibiotics at 3x MIC concentration. **bi** Anti-biofilm activity of HG2 against MRSA USA300 biofilms and **bii** Anti-biofilm activity of HG4 against MRSA USA300 biofilms. (A total of 30 biological replicates were conducted per concentration per AMP treatment. The anti-biofilm activity plot (% biofilm reduction) was generated using the ‘ggplot2’ package in R with log-linear regression performed using lm() function. Black line = 50% reduction of the biofilm. Blue line = the log-linear regression with the grey area representing a 95% confidence level interval for predictions from the model. The *R*^2^ value of the regression are 0.74 for HG2 and 0.77 for HG4. **c** Resistance acquisition during serial passaging of MRSA USA300 cells in the presence of sub-MIC levels of antimicrobial agents. The y axis is the fold change in MIC during passaging. For mupirocin, 32x MIC was the highest concentration tested. The figure is representative of three independent experiments **d** ATP depletion activity in MRSA USA300 cells. Error bars (±) represent the standard deviation from the mean.

The bactericidal activity of HG2 and HG4 against logarithmic-phase MRSA USA300 cells was investigated by time-kill kinetic studies. Compared to vancomycin and mupirocin, HG2 and HG4 (at 3x MIC concentration) had a rapid bactericidal activity against the MRSA USA300 strain (Fig. 3a), causing reductions of >3 log_10_ CFU/ml and >6 log_10_ CFU/ml, respectively, within the first 10 min. HG2 and HG4 induced complete cell death within 10 min of treatment, with no recovery observed after 24 h of incubation. This rapid and total loss in bacteria cell viability is similar to the killing kinetics that have been reported for many fast-acting antimicrobial peptides^14,31^. As expected, vancomycin and mupirocin at 3x MIC produced ≥2 log_10_ CFU/ml reductions attributable to differences in kill kinetics and mode of action^32^.

#### Anti-biofilm activity

We utilised a 96-well biofilm model^14^ to investigate the ability of HG2 and HG4 to dislodge/disrupt and disperse already formed and established MRSA USA300 biofilms. In general, HG2 and HG4 at sub-MIC (0.25x and 0.5 x MIC) and MIC concentrations have poor anti-biofilm activity, causing less than 50% reduction in biofilm formation. Whereas at supra-MIC concentrations (2x and 4x MIC), both HG2 (Fig. 3bi) and HG4 (Fig. 3bii) are effective against biofilms causing ≥50% biofilm reduction. The anti-biofilm activities of HG2 and HG4 albeit promising may indicate that further modification will be required for their potential suitability as agents for the disinfection of medical devices as well as in the treatment of biofilm infections. Also, see Supplementary Fig. 2 for representative raw data (optical density) output showing anti-biofilm activity in heatmap format.

#### Selection for resistance (serial passage)

Although relatively uncommon, bacterial resistance to cationic antimicrobial peptides is an evolving phenomenon^33^. Resistance to many AMPs including polymyxin B has recently been reported^34^, and it is, therefore, important to understand bacterial resistance to AMPs and to identify and design more robust AMPs. Other mechanisms of resistance to AMPs, which are mostly non-specific and confer moderate levels of resistance^35^, are mainly based on changes in the physicochemical properties of surface molecules and the cytoplasmic membrane^33^. For therapeutic AMP candidates, it is important that bacterial AMP resistance, which may develop due to selective pressure, is not based on mutations or acquisition of specific resistance genes, which can then be horizontally transferred between bacteria species as with conventional antibiotics^36^. Here, we assessed the likelihood of resistant mutants and/or resistance arising when MRSA cells are exposed to sub-MIC levels of HG2 and HG4. Continuous exposure of bacteria cells to sub-lethal doses of the AMPs over a period of 20 days did not produce resistant mutants (Fig. 3c), and MICs remained within 1–2-fold increases compared with mupirocin treated cells, which had a 32-fold MIC increase within the same period. The observed increase in MIC is common for many AMP-based molecules as a small change in the MIC after exposure to the AMP is to be expected^37^. Our inability to recover resistant mutants in this experiment suggests that the HG2 and HG4 may have non-specific or multiple cellular targets as has been described previously for other peptides^38^.

### Biochemical mode of action studies

#### ATP depletion assay

Adenosine triphosphate (ATP) is a high-energy nucleoside triphosphate molecule formed in the cytosol of bacteria and mitochondria and cytosol of eukaryotes driving most cellular and metabolic processes within cells^39^. Changes in the concentration of intracellular ATP can be used as an indicator of cell viability. We tested the effect of HG2 and HG4 on ATP concentration levels in MRSA USA300. As expected, untreated bacterial cells generated increasing amounts of ATP over time, whereas significantly lower concentrations of ATP were observed in HG2 and HG4-treated cells (Fig. 3d). This decrease in ATP concentrations may be an indication of ATP depletion, limiting cellular energy and thus other related cellular processes (such as substrate transport, homoeostasis and anabolism), likely linked to cell membrane disturbance. HG2 and HG4 induced a significant (*P* = 0.018 and 0.003, respectively) decrease in ATP concentration in *S. aureus* MRSA USA300 cells, which is similar to what was reported for other cationic AMPs^28^. Hilpert et al.^28^ demonstrated that many short AMPs had a strong effect on ATP concentration whereas several 26 mer α-helical peptides did not.

#### Bacterial membrane permeabilisation assay

**Fig. 4:**
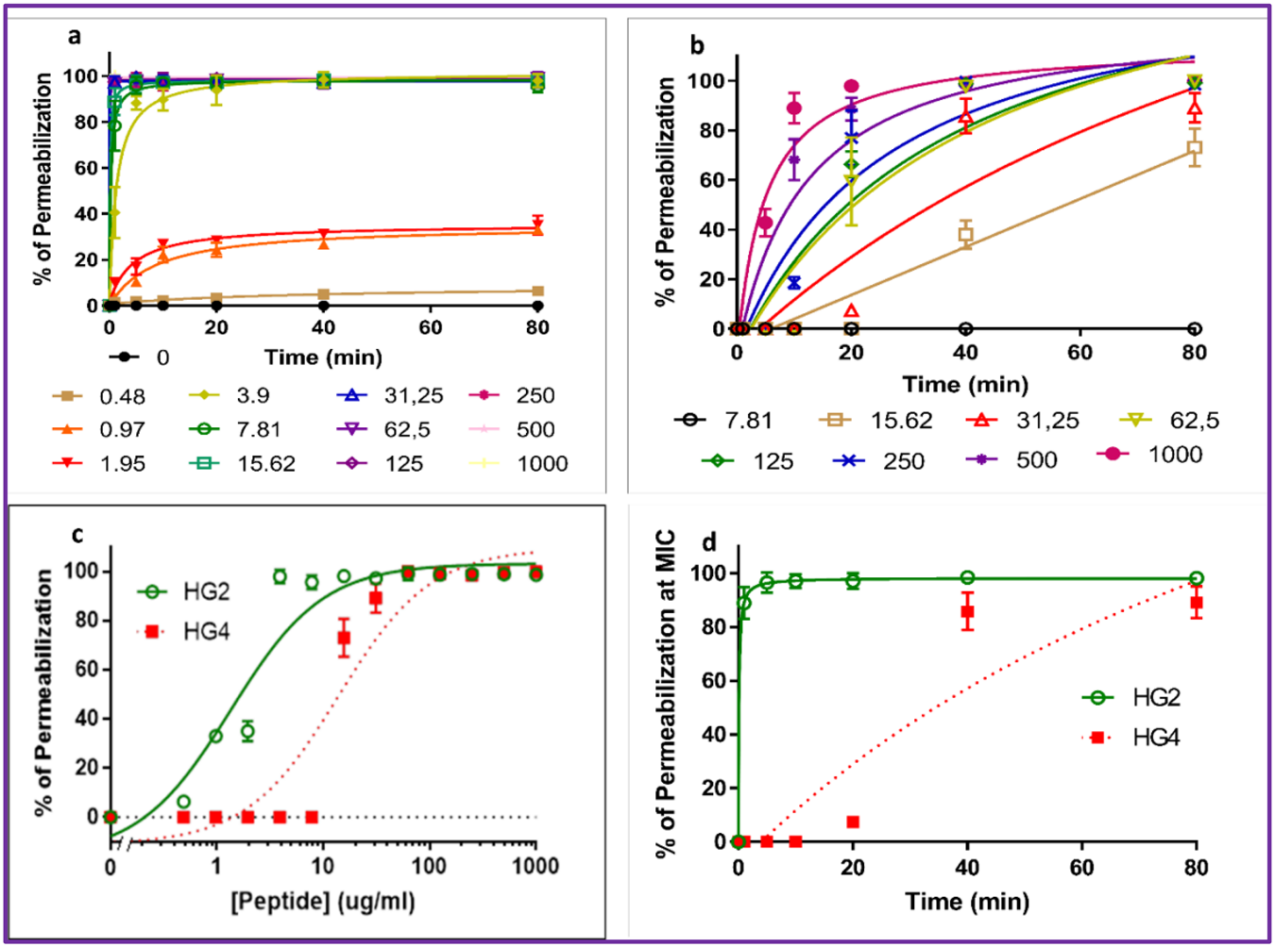
Membrane permeabilisation action of HG2 and HG4 against MRSA USA300 cells. **a** Membrane permeabilisation activity of HG2 at different concentrations (µg/ml) against MRSA USA300 cells measured by propidium iodide assay over time. **b** Membrane permeabilisation activity of HG4 at different concentrations (µg/ml) against MRSA USA300 cells measured by propidium iodide assay over time. **c** Determination of EC50 (effective concentration 50) of HG2 and HG4 membrane permeabilisation measured after 80 min. **d** Membrane permeabilisation kinetics of HG2 and HG4 at their MIC concentration. In all cases, values are from three independent replicates; results are expressed as means ± standard deviation.

Since HG2 and HG4 cause a rapid bactericidal effect, we used the propidium iodide (PI) method^14,40^ to determine if these AMPs were able to permeabilise MRSA USA300 cytoplasmic membrane similar to what was observed for other rumen-derived AMPs^14^. MRSA USA300 cells exposed to increasing concentrations of HG2 or HG4 showed increases in PI entry/fluorescence over time, demonstrating that they were able to permeate the cytoplasmic membrane and therefore indicating that they may possess pore-forming activity (Fig. 4a, b). Indeed, significant permeabilisation (*p* < 0.01) of MRSA USA300 cytoplasmic membrane was observed at sub-MIC concentrations as low as 1 and 16 µg/ml for HG2 and HG4, respectively (MIC values being 16 and 32 µg/ml for HG2 and HG4 respectively). The Effective Concentration 50 (EC_50_) (defined as the concentration of a drug at which the drug is half-maximally effective) of HG2 and HG4 measured after 80 min of incubation were 1.351 ± 0.27 and 13.85 ± 3.22 µg/ml, while total membrane permeabilisation was observed at 3.9 and 62.4 µg/ml, respectively (Fig. 4c). Membrane permeabilisation kinetics of HG2 and HG4 at their MIC concentration showed that HG2 was able to permeabilise the membrane faster (80% permeabilisation at 1 min and maximal effect after 5 min) than HG4 (minor permeabilisation at 20 min, maximal effect after 40 min) (Fig. 4d).

### Transmission electron microscopy

**Fig. 5:**
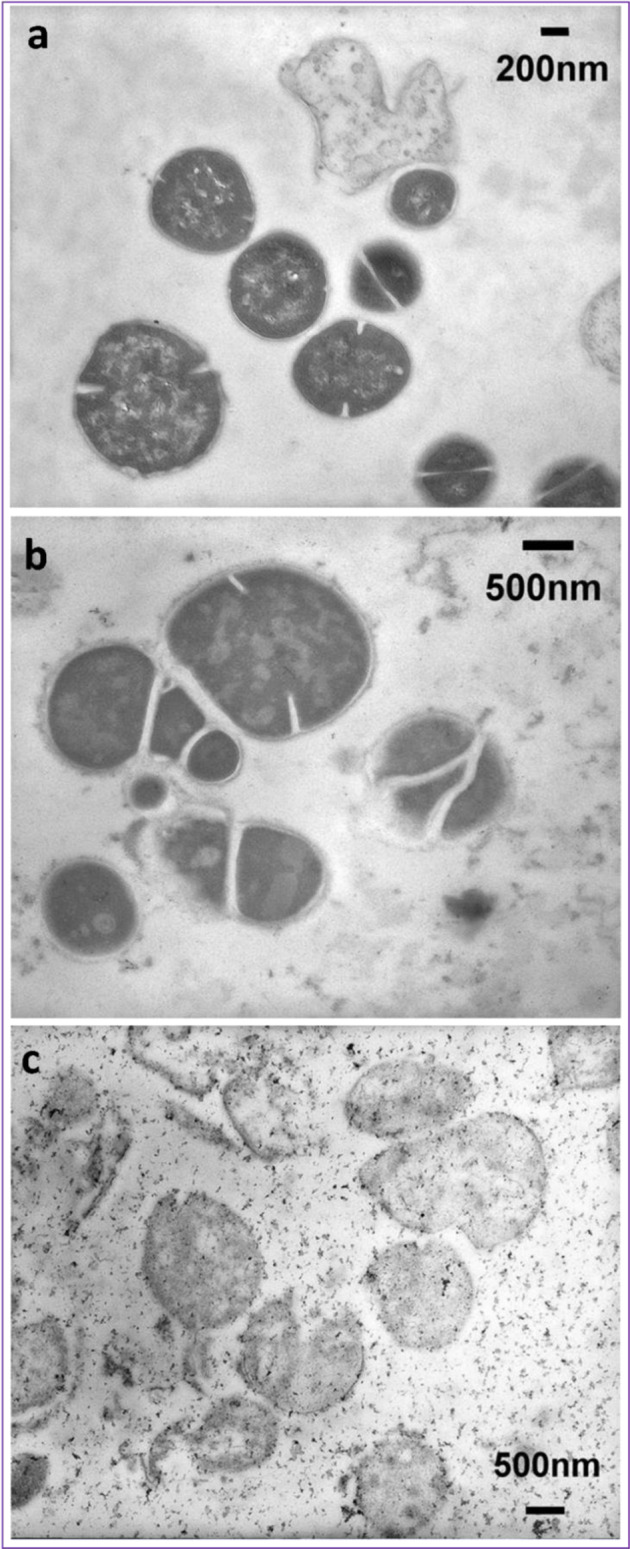
Representative transmission electron micrographs of MRSA USA300 cells. **a** Untreated MRSA USA300 cells. **b** HG2-treated (3x MIC for 1 h) MRSA USA300 cells. **c** HG4-treated (3x MIC for 1 h) MRSA USA300 cells. Scale bars are 200 or 500 nm as shown on micrographs.

Transmission electron micrographs (TEM) of cells treated with HG2 and HG4 at 3x MICs for 60 min revealed changes in cell morphology and some cytoplasmic damage (Fig. 5). The morphological changes observed in the HG2 and HG4-treated MRSA USA300 cells correspond with the membrane permeabilisation activity of the peptides.

### In vitro and ex vivo innocuity and cytotoxicity studies

#### Haemolytic activity

To establish the potential of HG2 and HG4 as therapeutic agents, their haemolytic effects were tested on human red blood cells. HG2 and HG4 induced low haemolysis, with HC_50_ (i.e. the concentration of peptide causing 50% haemolysis) of 409 ± 67 and 458 ± 101 µg/ml, respectively, with a therapeutic window (safety factor) of 26.2 and 14.6X MIC respectively (Supplementary Table 6).

#### Cytotoxicity studies on human primary cells and cells lines

Cytotoxicity of HG2 and HG4 against different human cell types was evaluated by measuring the IC_50_, which is the concentration of peptide inhibiting 50% of the cell viability. Lung fibroblast (i.e. IMR-90) cells were found to be the most sensitive to HG2 and HG4 with IC_50_ of 96 ± 21 and 294 ± 42 µg/ml for HG2 and HG4. Lung epithelial (i.e. BEAS-2B) and liver (i.e. HepG2) cells were the least sensitive to HG2 and HG4 with IC_50_ of 120 ± 25 and 359 ± 76 µg/ml for HG2 and IC_50_ >1000 µg/ml for HG4 (see Supplementary Table 6). Overall, cytotoxicity data showed that HG4 was less toxic than HG2 for human cells and that epithelial cell types (i.e. BEAS-2B and HEPG2) were the least sensitive, while fibroblast cells (i.e. IMR-90) were more susceptible to the peptides (Supplementary Table 6). The high hydrophobicity of HG2 may contribute to its higher toxicity to human erythrocytes and cell lines. This is similar to Gramicidins that possess high hydrophobicity ratios and are exclusively used topically due to their haemolytic side-effects^41^. Although HG2 might be feasible for use in other applications, it can not be excluded that it might be restricted to topical applications to treat superficial infections, unless modified derivatives/analogues of HG2 with improved cytotoxicity become available.

### Peptide–lipid interaction and insertion assay

**Fig. 6:**
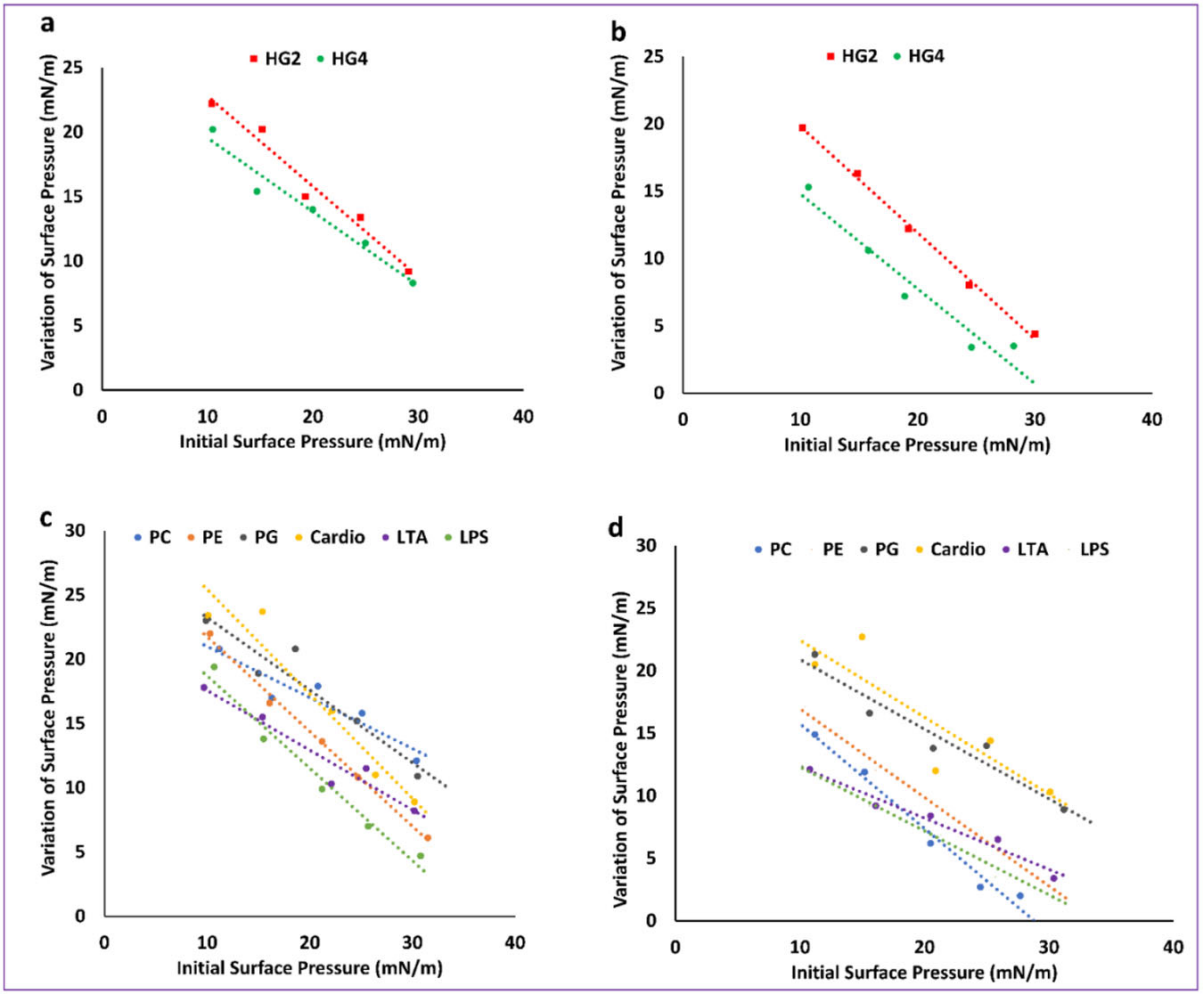
Peptide–lipid interaction and insertion measurements. Interaction of HG2 and HG4 (at 1 µg/mL final concentration) with lipids (either total lipid extracts or pure lipids) was measured using lipid monolayers. **a** interaction HG2 and HG4 with total MRSA lipid extract. **b** interaction HG2 and HG4 with total lipid extract from human erythrocytes. **c** interaction of HG2 with pure lipids and **d** interaction of HG4 with pure lipids. 1-palmitoyl-2-oleoyl-*sn*-glycero-3-phospho-(1’-rac-glycerol) (PG), 1-palmitoyl-2-oleoyl-*sn*-glycero-3-phosphoethanolamine (PE), Cardiolipin (Cardio), Lipoteichoic acid (LTA) from *S. aureus*, Lipopolysaccharide (LPS) from *E. coli* and (1-palmitoyl-2-oleoyl-glycero-3-phosphocholine (PC).

The interaction of HG2 and HG4 with lipid monolayers was measured by the critical pressure of insertion, reflecting the affinity of the peptides for specific lipids^14^. Insertion capacity was first measured using total lipid extracts obtained from MRSA USA300 cells or human erythrocytes (Fig. 6a, b and Supplementary Table 7) and obtained data suggested that HG2 and HG4 had higher affinity and insertion ability in MRSA lipids, with critical pressure of insertion of 35.07 and 30.99 mN/m and 42.59 and 44.18 mN/m for HG2 and HG4 in MRSA and erythrocyte lipids, respectively. These results show that HG4 is less able to insert into erythrocyte lipids than HG2 in accordance with the lower haemolytic activity observed in HG4 compared to HG2 (HC_50_ of 409 and 458 µg/ml for HG2 and HG4, respectively).

To identify the lipid partner(s) recognised by HG2 and HG4 in bacterial and eukaryotic membranes, measurement of the critical pressure of insertion in pure lipids were performed (Fig. 6c, d and Supplementary Table 7). Results indicated that HG2 and HG4 had different affinities and insertion capacities for pure lipids from bacteria or eukaryotes. Whereas HG4 interacts preferentially with bacterial lipids expressed in the outer leaflet of the membrane (1-palmitoyl-2-oleoyl-*sn*-glycero-3-phospho-(1’-rac-glycerol) (PG), 1-palmitoyl-2-oleoyl-*sn*-glycero-3-phosphoethanolamine (PE), cardiolipin, lipoteichoic acid (LTA) from *S. aureus*, lipopolysaccharide (LPS) from *E. coli* over eukaryotic lipids (1-palmitoyl-2-oleoyl-glycero-3-phosphocholine—PC) (i.e. PG > cardiolipin > LTA > LPS > PE > PC), HG2 displayed selectivity, with first the major lipid present in the outer leaflet of the eukaryotic membrane (PC) followed by bacterial membrane lipids (i.e. PC > PG > LTA > Cardio > PE > LPS). These observations are in accordance with the higher toxicity of HG2 (high susceptibility of human cells to HG2) against human cell lines compared to HG4 (Fig. 6c, d and Table S7). Interestingly, HG2 and HG4 insert into LPS, albeit less efficiently than LTA. This corresponds to the low antimicrobial activity of HG2 and HG4 against Gram-negative bacteria compared to their potent activity against Gram-positive bacteria. Measurement of the speed of insertion of HG2 and HG4 in total lipid extracts or pure lipids at an initial surface pressure of 30 ± 0.5 mN/m (corresponding to the theoretical surface pressure of eukaryotic and prokaryotic membranes) showed that HG2 inserts faster into all lipid monolayers compared to HG4 (Supplementary Table 7), confirming results obtained in the membrane permeabilisation assay, which indicated faster bacterial membrane permeabilisation of HG2 when compared to HG4.

### Transcriptomic analysis of peptide activity

**Fig. 7:**
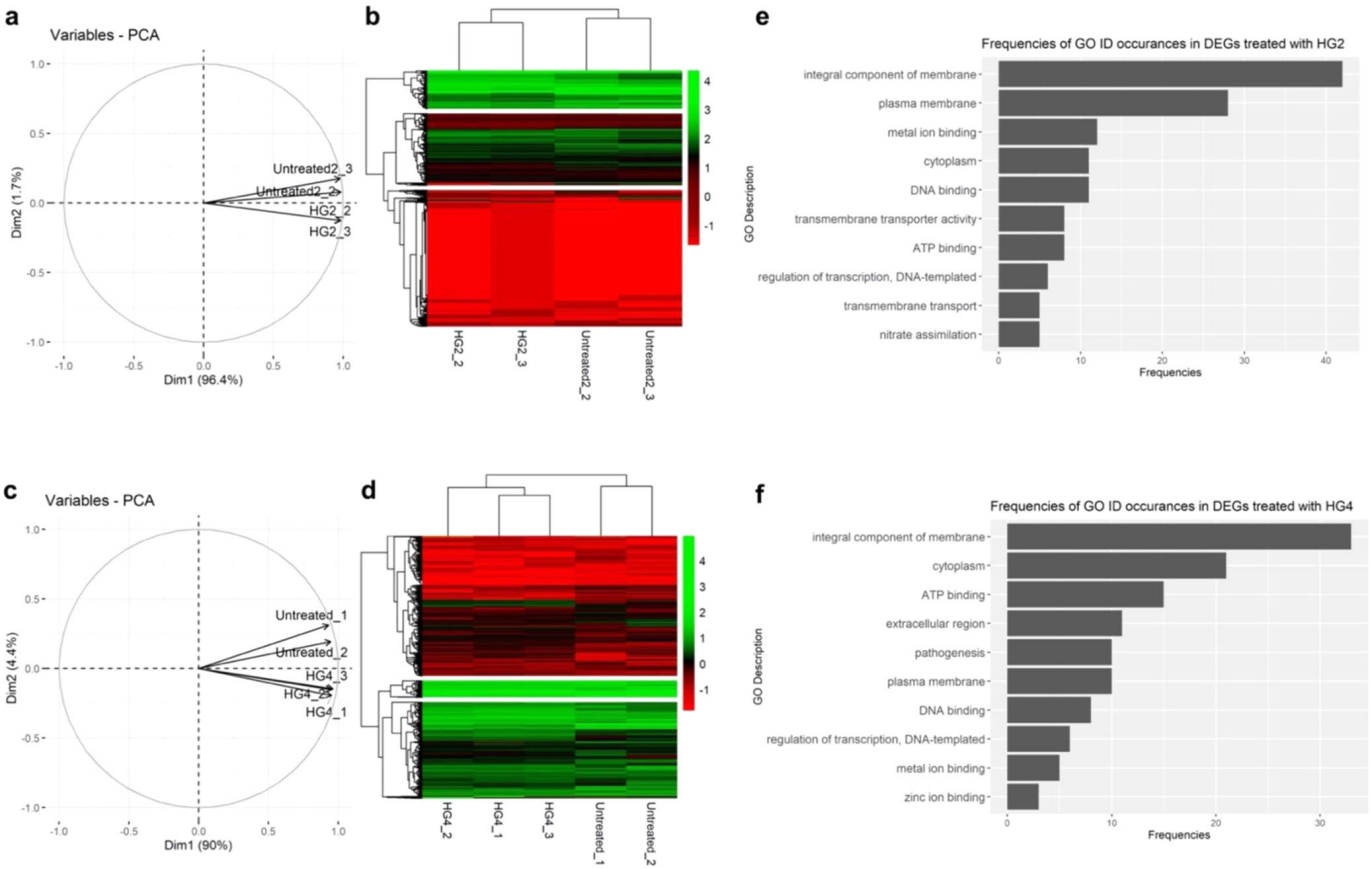
Peptide-induced transcriptional changes in MRSA USA300 cells. **a** Principal component plot and **b** Hierarchical clustering analysis plot in the HG2 treatment group after outlier(s) (HG2_1 and Untreated2_1) removal. **c** Principal component plot and **d** Hierarchical clustering analysis plot in the HG4 treatment group after outlier (Untreated_3) removal. Frequencies of top ten Gene Ontology (GO) terms in the DEGs in samples treated with **e** HG2 and **f** HG4. In total, 12 samples were sequenced (six per HG2 treatment group i.e., HG2_1, HG2_2, HG2_3, Unt_1, Unt_2 and Unt_3 and six per HG4 treatment group including HG4_1, HG4_2, HG4_3, Unt_1, Unt_2 and Unt_3). As the HG2 treatment experiment, was performed separately from the HG4 treatment experiment with each group having their own distinct untreated sample groups, the analysis of each treatment group was performed separately.

We explored the transcriptional changes of *S. aureus* MRSA USA300 cells in response to HG2 and HG4 treatment at their MIC concentrations for 60 min. In total, 12 samples were sequenced (six per HG2 treatment group i.e., HG2_1, HG2_2, HG2_3, Unt_1, Unt_2 and Unt_3 and six per HG4 treatment group including HG4_1, HG4_2, HG4_3, Unt_1, Unt_2 and Unt_3). As the HG2 treatment experiment, was performed separately from the HG4 treatment experiment with each group having their own distinct untreated sample groups, the analysis of each treatment group was performed separately. A total of ~285 million quality-filtered reads (15 Gb of data), with an average length of 75 or 125 bp for HG2 and HG4 sample groups respectively were generated, and a similar quantity of mapped reads to the reference genome (*Staphylococcus aureus* subsp. aureus USA300_TCH1516) were recovered. Hierarchical clustering and principal component analyses were used to determine the consistency of the controlled groups and the effect of the AMP treatments on MRSA USA300 bacterial cells. In both analyses, one HG2 treated and one untreated (control) sample displayed gene expression patterns which were different from the original groupings (Supplementary Fig. 3a, b). Thus, the two outlier samples were removed from further analysis (see Materials and methods session for outlier criteria). Similarly, one control sample which displayed similar expression patterns to the HG4-treated cells was removed from further analysis in the HG4 treatment group (Supplementary Fig. 3c, d). Figure 7a–d show the results for HG2 and HG4-treated samples groups after outlier removal with all remaining samples appearing to be consistent within the preassigned grouping. A total of 114 differentially expressed genes (DEGs) were identified from the HG2 treated group (see Supplementary Table 8). To obtain a functional overview of the gene list, the GO ID from all the DEGs were summarised into a count table (Supplementary Table 9, Fig. 7e). The top three most occurring GO IDs among the DEGs were GO:0016021, GO:0005886 and GO:0046872 with an abundance of 13.5, 9.0 and 3.9% respectively. Each of these terms represents genes involved in the ‘integral component of membrane’, ‘plasma membrane’ and ‘metal ion binding’ functions of the cells. This is in line with the membrane acting mechanism observed in the membrane permeabilisation assay and TEM assay for HG2. In the HG4-treated group, DESeq2 analysis yielded 123 DEGs (Supplementary Table 10). The GO ID from all DEGs were summarised into a count table (Supplementary Table 11 and Fig. 7f), with the top occurring GO IDs being GO:0016021 (11%), GO:0005737 (7%) and GO:0005524 (5%). These DEGs represented genes involved in the ‘integral component of membrane’, ‘cytoplasm’ and ‘ATP binding’ functions of the cells, corroborating the membrane action and ATP depletion observed for HG4.

### Peptide anti-inflammatory activity

**Fig. 8:**
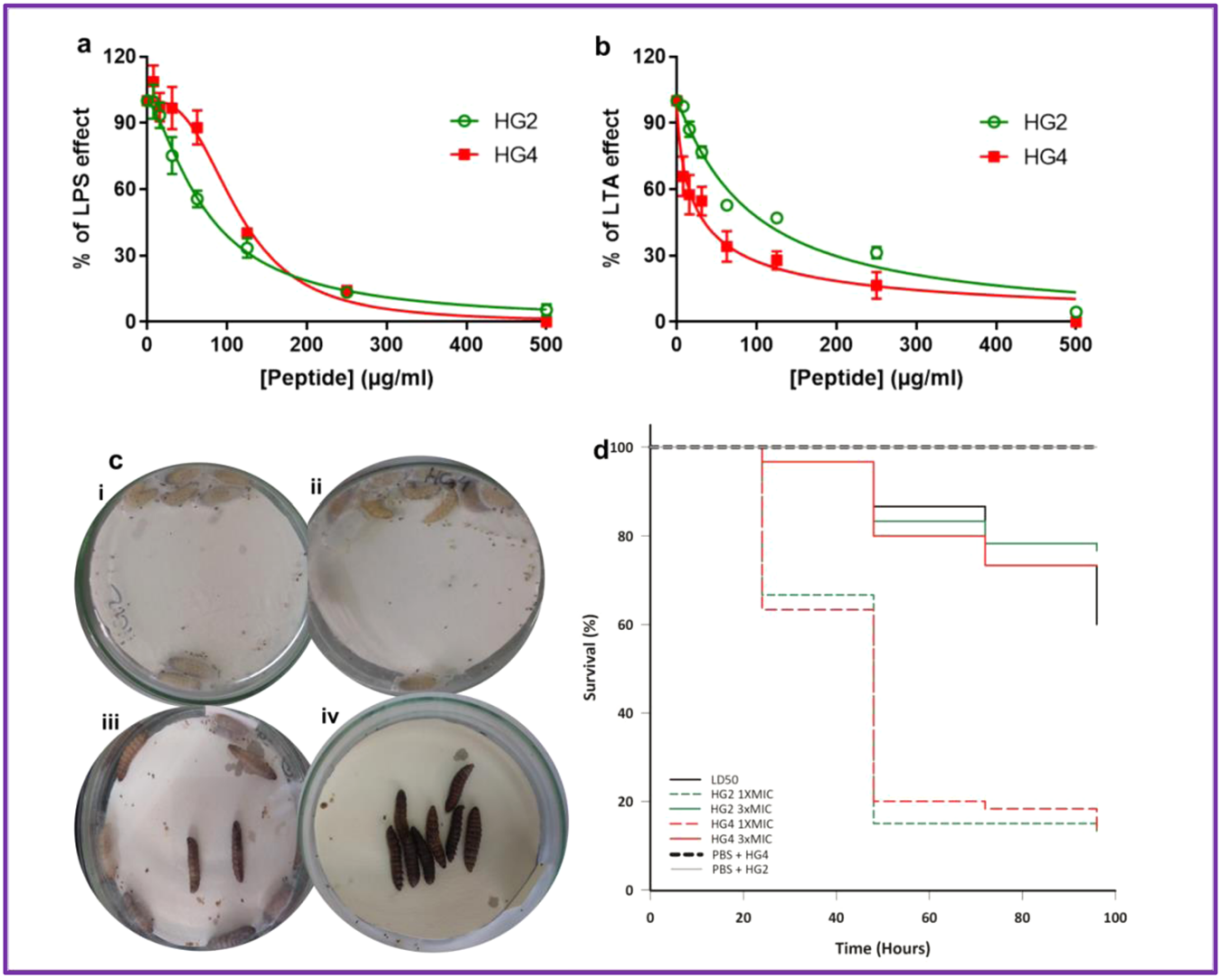
Peptide anti-inflammatory activity in murine macrophages and in vivo efficacy assessment in *G. mellonella* MRSA infection model. Inhibitory action of HG2 and HG4 on LPS or LTA-driven inflammation in murine macrophages: eLUCidate™ Raw 264.7 NF-kB reporter cell line was used to measure the inhibitory action of HG2 and HG4 on LPS (**a**) or LTA (**b**) -mediated inflammation as explained in the Materials and Methods’ section. Data were plotted using GraphPad® Prism 7 software. Results are expressed as means ± standard deviation. Representative images of toxicity assay of peptides (**c**)**—**(**i**) HG2 and (**ii**) HG4 in *G. mellonella* 120 h post-treatment with 3x MIC concentrations. The larvae remained alive and without melanisation. Virulence assay of MRSA USA300 in *G. mellonella* using a lethal dose inoculum of 10^6^ CFU/per larvae—(**iii**) 24 h post-infection: some larvae were dead and partial melanisation was observed. (**iv**). 48 h post-infection: most larvae were dead and complete melanisation was evident. The experiment was done with three experimental replicates, each containing groups of 10 larvae. **d** Kaplan–Meier survival curves of *G. mellonella* infected with a lethal dose (LD_50_) of *S. aureus* (2.25 × 10^6^ CFU/larvae) and treated with peptides HG2 and HG4 at 1x and 3x MIC concentrations and uninfected larvae treated with peptides in PBS at 3x MIC (placebo showing a 100% larvae survival rate).

In addition to their antimicrobial effect, some AMPs have been shown to possess anti-inflammatory activity^42^. The potential inhibitory effect of AMPs against inflammation that is driven by bacterial molecules such as lipopolysaccharide (LPS) and lipoteichoic acid (LTA) are of particular importance. Indeed, an antibacterial activity associated with an anti-inflammatory activity would be a clear benefit compared with conventional antibiotics devoid of such effect in the treatment of infections. The potential anti-inflammatory activity of HG2 and HG4 on LPS- or LTA-driven inflammation was investigated using eLUCidate™ Raw 264.7 NF-kB reporter cell line (Fig. 8a, b). It was observed that both HG2 and HG4 possess dose-dependent inhibitory effects on LPS and LTA-mediated inflammation. Regarding the inflammation caused by the LPS from *E. coli*, the IC_50_ values of HG2 and HG4 were 73.99 ± 3.40 and 113.80 ± 5.00 µg/ml respectively, demonstrating a 1.5-fold higher inhibitory effect of HG2 over HG4. In the case of inflammation caused by the LTA from *S. aureus*, the IC_50_ values of HG2 and HG4 were 91.49 ± 6.03 and 26.45 ± 3.11 µg/ml respectively, demonstrating a 3.45-fold higher inhibitory activity of HG4 over HG2. The observed anti-inflammatory effect of HG2 and HG4 could not be explained by a cytotoxic effect of the peptides since a resazurin assay showed no toxicity of HG2 or HG4 against the eLUCidate™ Raw 264.7 NF-kB reporter cell line during the time of the assay (data not shown). Overall, it seems that HG2 is more active in LPS (Gram-negative)-mediated inflammation whereas HG4 is more active in LTA (Gram-positive) effect. Control inhibitory molecules Pyrrolidine dithiocarbamate (PDTC) and polymyxin B (PMB) gave IC_50_ values for LPS or LTA effect of 10.68 ± 1.20 or 10.85 ± 1.29 µg/ml (PDTC), and 62.26 ± 4.01 or 61.34 ± 3.47 µg/ml (PMB), showing no selectivity in that case. Notably, the inhibitory effect of HG2 and HG4 on LPS/LTA-driven inflammation is observed at concentrations close to or even lower than the ones causing antibacterial effect (i.e. MIC of 16 to 256 µg/ml on *S. aureus* and 256 to 512 µg/ml on *E. coli*).

### In vivo efficacy studies in *Galleria mellonella* infection model

*Galleria mellonella* has been used widely as an effective in vivo model for testing antimicrobial drugs and their toxicity^43,44^. We investigated the toxicity of HG2 and HG4 against *G. mellonella* larvae as well as their ability to protect the larvae from a lethal dose of an MRSA USA300 infection. The peptides HG2 and HG4 showed no toxic effect in *G. mellonella* at either 1x MIC or 3x MIC concentrations (Fig. 8c). As with the control group of larvae, no phenotypic modification such as melanisation and/or motility reduction was observed in larvae injected with HG2 and HG4.

The MRSA USA300 infective dose (LD_50_) was determined as being 10^5^ CFU/larvae, while the lethal dose (LD) was determined as 2.25 × 10^6^ CFU/larvae and caused melanisation and death of all larvae within 24 h (Fig. 8c). Larvae infected with MRSA USA300 LD and treated with peptides HG2 and HG4, at 1x MIC concentration had a survival rate that was ~20% higher than the control group (Fig. 8d). In comparison to larvae in the untreated control group, larvae infected with MRSA USA300 LD, followed by treatment with peptides at 3x MIC increased survival by 4.6-fold and 4.4-fold for HG2 and HG4 with a survival rate of 78 and 75%, respectively (Fig. 8d).

We were able to show that both peptides, especially at 3x MIC, concentration can effectively control MRSA USA300 infection in *G. mellonella*. Other pharmacological aspects of the peptides need to be investigated in order to improve the efficacy of HG2 and HG4, as the survival rate of AMP-treated larvae was comparable to the LD_50_ survival rate, probability due to distribution and/or bioavailability of the peptides in the *G. mellonella* model. Nonetheless, our results show that the peptides HG2 and HG4 at 3x MIC concentration are capable of significantly improving the survival of larvae infected with a lethal dose of MRSA USA300 and are active against this clinically important drug resistant pathogen in an in vivo model.

## Discussion

The two AMPs, HG2 and HG4, identified from a rumen metagenomic dataset further confirms the microbial community within the rumen is not only of great importance to the host animal but can also serve as an invaluable resource for urgently needed alternatives to currently available antibiotics. Furthermore, the study presented here emphasises the usefulness of complementing wet-lab and in silico techniques for the rapid identification of new AMP candidates from environmental sample datasets. The low similarity of the newly identified AMPs to previously known sequences suggests their novelty from an evolutionary point of view. Experimental evaluation and characterisation of the antimicrobial properties of HG2 and HG4, two of the identified AMP candidates, revealed their antimicrobial activity against Gram-positive bacteria. The findings from membrane permeabilisation, transcriptomic and transmission electron microscopy analysis suggest that membrane permeabilisation and a decrease in intracellular ATP concentration might play a role in their antimicrobial activity against MRSA USA300. Both HG2 and HG4 preferentially bind to MRSA total lipids rather than with human erythrocyte lipids. HG4 was less cytotoxic against all cell lines tested and was observed to bind more specifically to pure bacterial membrane lipids, indicating that HG4 may form a superior template for a safer therapeutic candidate than HG2. The non-toxic effect of the peptides against *G. mellonella* larvae, their in vivo efficacy against MRSA USA300 infection in the *G. mellonella* infection model, as well as their inhibitory effect on LPS and LTA-mediated inflammation suggests that these peptides might possess potential as safe alternative therapeutics with anti-biofilm activity for the treatment of bacterial infections. Given the technological advances, improvements in genomic methods and computational analytic approaches as well as the growing abundance of omics data, the approach developed and presented here, alongside other rational design^30^ and deep learning^11^ approaches will facilitate the discovery of novel AMPs and other bioactives from environments where conventional isolation and cultivation of microorganisms has been challenging.

## Methods

### In silico identification of AMPs using computational analysis

Antimicrobial peptide prediction and similarity analysis was performed on the rumen metagenomic dataset from the study by ref. ^17^. The dataset was termed the Library ‘Cow’ dataset and contains 2,547,270 predicted protein sequences (‘metagenemark_predictions.faa.gz’) which were downloaded from the weblink (http://portal.nersc.gov/project/jgimg/CowRumenRawData/submission/). All other datasets (libraries) used for similarity analysis prediction/identification of novel AMP candidates from the ‘Cow’ dataset and their respective sources are as follows: The Library ‘AMP1’, which contained a list of 2308 known antimicrobial peptides (AMPs) downloaded from APD2^45^ (downloaded on November 10, 2013 and available at http://aps.unmc.edu/AP/main.php) and the Library ‘AMP2’, which contains a list of 48 synthetic AMPs (Hilpert Library) identified by ref. ^46^. The MATLAB toolbox Gait-CAD and its successor SciXMiner (http://sourceforge.net/projects/scixminer/)^47^ including the Peptides Extension Package^20^ were used for the computational analysis unless otherwise stated.

The ‘fastread’ function of the MATLAB Bioinformatics toolbox was used to import the ‘Cow’ dataset. For easier computational analysis, the imported dataset was then split into 26 parts with ~100,000 sequences each. Following recommendations that small antimicrobial proteins should have a length <200 amino acids (AAs), with most AMPs (>90%) on the APD2 database having a length of <60 AAs^45^, only protein sequences with a maximum length of 200 AAs^19^ and not more than 5% unknown AAs (marked by X, *) were selected from the ‘Cow’ dataset predicted protein sequences (metagenemark_predictions.faa.gz). Libraries ‘AMP1’ and ‘AMP2’ were combined to produce Library ‘AMP’ composing a total of 2356 peptides. Thereafter, AA distribution and AA dimer (pair) distribution were computed, resulting in a proportion for 20 AAs (and 20 × 20 = 400 AA dimers) for Libraries ‘Cow’ and ‘AMP’. Pairwise distances of AA acid distributions between two peptides (termed 'AAD') were computed with a minimal value of 0 for identical and increasing values for different AAs. Pairwise distances of AA acid and AA acid pair distributions between two peptides were computed respectively (distance for 400 + 20 features), termed ‘AAPD’. For each candidate of Library ‘Cow’, the values of ‘AAD’ and ‘AAPD’ to each peptide in Library ‘AMP’ were computed. Again, for each sequence in Library ‘Cow’, minimal distance values from the previous computational step and the number of the most similar peptides from Library “AMP” were saved as separate features. To select only promising candidates for AMP predictions, only sequences from the Library ‘Cow’ with small AA distances AAD <0.2 or a small AA and AA pair distances AAPD <1.45 were saved. The distances were computed using the 1-norm (Manhattan norm)^20^. The thresholds for AAD and AAPD were heuristically chosen to balance the trade-off between too many weak candidates (too high values of AAD and AAPD) vs. the loss of promising candidates (too low values of AAD and AAPD). All sequences that fulfilled the conditions in the preceding step were collected and some randomly selected hits were used to check similarity in the APD2 database (criteria: small values of AAD or AAPD, different neighbours to explore the variety of the candidates found, short peptide length). Finally, descriptors were computed following procedures described by refs. ^20,21^ to check the expected balance between hydrophobicity and positively charged AAs as a typical design criterion for AMPs.

### Peptide synthesis and three-dimensional modelling of peptide structures

Pure (≥95% purity) peptides were synthesised on resin using solid-phase Fmoc peptide chemistry^24^ by GenScript Inc. USA. A de novo structural prediction method, PEP-FOLD^48^ was used to model the 3D conformation of peptides HG2 and HG4. About 200 simulations were run for each peptide and resulting conformations were clustered and ranked using the sOPEP coarse-grained force field^49^. In the case of HG2, the formation of the cysteine bond between Cys10 and Cys18 was imposed as a restraint to the simulation. The best 3D models for each peptide were manually analysed using PyMOL v1.7.6^50^.

### Antimicrobial susceptibility of bacterial cells

To determine the antimicrobial activity of the new antimicrobial peptides, HG2 and HG4, their minimum inhibitory concentrations (MICs) were determined by broth microdilution method^51^ in cation adjusted Mueller Hinton broth (MHB) following the International Organisation for Standardisation (ISO) 20776-1 standard for MIC testing using a final bacterial inoculum of 5 × 10^5^ CFU/ml^52^. The lowest concentration of the AMPs that inhibited the visible growth of the bacteria was tested after overnight incubation at the appropriate temperatures (37 °C for all organisms except for *Listeria monocytogenes*, which was grown at 30 °C) was defined as the MIC. The peptides dissolved in sterile distilled water were added to bacteria culture and incubated overnight at appropriate conditions. The MICs of the peptides and comparator antibiotics were recorded after 18–24 h. The minimum bactericidal concentrations (MBCs) were also determined and were taken as the lowest concentration of the antimicrobial that prevented the growth of bacterial cells after the subculture of cells (from MIC treatment) onto antibiotic-free media.

### Time-kill kinetics

The bactericidal activity of HG2, HG4 and comparator antimicrobial compounds was assessed using exponential-phase cultures^14^ of MRSA USA300 grown in MHB (1 × 10^6-8^ CFU/ml). Cells were treated with antimicrobial compounds at three times their MIC concentrations (final concentrations) and incubated at 37 °C with gentle shaking at 110 rpm. Samples were taken at different time points, serially diluted and inoculated onto MH agar plates using the spread plate technique. After overnight incubation, the colony-forming units per millilitre of cell culture (CFU/ml) was calculated. Experiments were performed in quadruplicates.

### Anti-biofilm activity

The ability of HG2 and HG4 to disrupt established *S. aureus* biofilms was measured using a 96-well format as described by ref. ^14^. MRSA USA300 cultures grown overnight in Brain Heart Infusion (BHI) broth was resuspended to OD_600nm_ = 0.02 and grown without aeration at 37 °C in 96-well tissue culture plates for an additional 24 h. The planktonic cells were removed by three PBS (phosphate-buffered saline) washes. Thereafter, fresh BHI broth containing peptides HG2 or HG4 at sub- and supra-MIC concentrations (0.5, 1, 2 and 4x MIC) was added to wells containing adherent cells and incubated without shaking for another 24 h. Planktonic cells were again removed by three PBS washes. Biofilms were fixed with methanol for 20 min, stained with 0.4% (w/v) crystal violet solution for 20 min and re-solubilised with 33% (v/v) acetic acid. The optical density of re-solubilised biofilms was measured at 570 nm in a microplate spectrophotometer. The percentage biofilm reduction was calculated using the equation below:

$${{{\mathrm{Percentage}}}}\,{{{\mathrm{biofilm}}}}\,{{{\mathrm{reduction}}}}({{{\mathrm{\% }}}}) = \frac{{{\mathrm{Positive}}\,{\mathrm{Control}} - {\mathrm{OD}}\,{\mathrm{of}}\,{\mathrm{Sample}}}}{{{\mathrm{Positive}}\,{\mathrm{Control}} - {\mathrm{Negative}}\,{\mathrm{Control}}}}$$

The plot showing anti-biofilm activity (% biofilm reduction) was generated using the ‘ggplot2’ package^53^ in R (v 4.1.1)^54^ with log-linear regression performed using lm() function.

### Serial passage/resistance assay

In vitro evaluation of the potential for MRSA USA3000 cells to develop resistance to HG2 and HG4 was performed as follows^14^. Briefly, on Day 1, overnight cultures grown from a single colony of MRSA USA300 strain in MHB at 37 °C with shaking at 225 rpm was subjected to microbroth dilution susceptibility testing performed using a standard doubling-dilution series of AMP concentrations as described for MIC determination. Cultures from the highest concentration that supported growth were diluted 1: 1000 in MHB and used to provide the inoculum for the next passage day. This process was performed for a total duration of 20 days. Any putative mutants recovered were isolated via streak dilution for three generations on MHA, prior to further characterisation.

### ATP determination assay

Adenosine triphosphate (ATP) drives many cellular and metabolic processes and can be used to ascertain the integrity of cells^39^. To determine whether HG2 and HG4 affected ATP levels in *S. aureus*-treated cells, we used the ATP colorimetric/fluorometric assay kit (MAK190, Sigma-Aldrich, UK) which determines ATP concentration by phosphorylating glycerol, resulting in a colorimetric product that shows absorbance at 570 nm. Briefly, in a clear 96-well plate, samples and ATP standard provided with the kit were added in a reaction mix containing ATP assay buffer, ATP Probe and Converter and Developer mix. The reaction mix was incubated in the dark at room temperature for 30 min. Absorbance (570 nm0 was then measured in a microplate spectrophotometer. The background ATP levels obtained from samples and ATP standards were autocorrected by subtracting ATP levels from blank treatments. The amount of ATP in unknown samples was determined from the ATP standard curve and calculated using the formula in the instruction manual. All measurements were performed in triplicates.

### Membrane permeabilisation assay

Permeabilisation of the bacterial cytoplasmic membrane by HG2 and HG4 peptide was evaluated using the cell-impermeable DNA probe propidium iodide^14,40^. Logarithmic-phase bacterial suspension of MRSA USA300 was prepared by diluting overnight cultures in fresh MHB (1 in 10 dilutions), incubated (3 h; 37 °C; 200 rpm), and pelleted by centrifugation (5 min; 3000×*g*). The bacterial cell pellet was then resuspended in sterile PBS at ~10^9^ bacteria/ml. Propidium iodide (at 1 mg/ml, Sigma-Aldrich) was added to the bacterial suspension at a final concentration of 60 µM. This suspension (100 µl) was then transferred into black 96-well plates already containing 100 µl of serially diluted HG2 or HG4 peptide in PBS. Kinetics of fluorescence variations (excitation at 530 nm/emission at 590 nm) were then recorded using a microplate reader for a duration of 80 min with incubation at 37 °C. Cetyl trimethylammonium bromide (CTAB) (at 300 µM) served as positive control giving 100% permeabilisation. The permeabilisation effect of HG2 and HG4 were expressed as a percentage of total permeabilisation.

### Transmission electron microscopy

Transmission electron microscopy^14^ was used to investigate the effects of HG2 and HG4 on MRSA USA300 cell morphology. Mid-log phase *S. aureus* cultures treated with HG2 and HG4 (at 3× MIC for 1 h) were fixed with 2.5% (v/v) glutaraldehyde and post-fixed with 1% osmium tetroxide (w/v). They were then stained with 2% (w/v) uranyl acetate and Reynold’s lead citrate after which they were observed using a JEM1010 transmission electron microscope (JEOL Ltd, Tokyo, Japan) at 80 kV.

### Haemolytic activity

The ability of HG2 and HG4 to cause leakage of erythrocytes from human whole red blood cells was determined to ascertain probable cytotoxicity to mammalian cells and the suitability of peptides for use as therapeutic agents. The haemolytic activity of HG2 and HG4 was determined as follows^14^. Briefly, fresh human erythrocytes (Divbio Science Europe, NL) were washed 3 times by centrifugation at 800 × *g* for 5 min with sterile phosphate buffer saline (PBS, pH 7.4). The washed erythrocytes were resuspended in PBS to a final concentration of 8%. About 100 μl of human erythrocytes were then added to each well of sterile 96-well microplates already containing serial dilutions of the peptides in 100 µl of PBS. The treated red blood cells were incubated at 37 °C for 1 h and centrifuged at 800 × *g* for 5 min. The supernatant (100 μl) was carefully transferred to a new 96-well microplate and absorbance at 450 nm was measured using a microplate reader. Triton X-100 at 0.1% (v/v) was used as positive control giving 100% haemolysis. The haemolysis caused by HG2 and HG4 was expressed as a percentage of total haemolysis. The HC_50_ values for HG2 and HG4 (i.e. the concentration of peptide causing 50% of haemolysis) were calculated using GraphPad® Prism 7 software.

### Peptide–lipid interaction and insertion assay

Peptide–lipid interaction was measured using a lipid monolayer formed at the air:water interface with total lipid extracts and pure lipids. Total lipids were extracted from overnight cultures of MRSA or human erythrocytes using the Folch extraction procedure^14,40^. Extracted total lipids were dried, re-solubilised in chloroform:methanol (2:1, v/v) and stored at −20 °C under nitrogen. Pure prokaryotic and eukaryotic lipids used were: cardiolipin, POPC (1-palmitoyl-2-oleoyl-glycero-3-phosphocholine), POPE (1-palmitoyl-2-oleoyl-*sn*-glycero-3-phosphoethanolamine) and POPG (1-palmitoyl-2-oleoyl-*sn*-glycero-3-phospho-(1’-rac-glycerol) (Avanti Polar Lipid). LTA (Lipoteichoic acid from *S. aureus*) and LPS (lipopolysaccharide from *E. coli*) (Invitrogen, UK) were also tested. Pure lipids were reconstituted in chloroform:methanol (2:1 v/v) at 1 mg/ml and stored at −20 °C under nitrogen. For peptide–lipid interaction assay, lipid monolayers at the air:water interface were formed by spreading total lipid extract or pure lipids at the surface of 800 µl of sterile PBS using a 50 µl Hamilton’s syringe. Lipids were added until the surface pressure reached the desired value. After 5–10 min of incubation allowing the evaporation of the solvent and stabilisation of the initial surface pressure, 8 µl of HG2 or HG4 diluted in sterile PBS at 100 µg/ml were injected into the 800 µl sub-phase of PBS under the lipid monolayer (pH 7.4, volume 800 µl) using a 10 µl Hamilton’s syringe giving a final concentration of peptide of 1 µg/ml, as preliminary experiments had shown that this concentration was optimal. The variation of the surface pressure caused by peptide insertion was then continuously monitored using a fully automated microtensiometer (*µ*TROUGH SX, Kibron Inc, Finland) until it reached equilibrium (maximal surface pressure increase is usually obtained after 15–25 min). To reflect physiological situations, the initial surface pressure was fixed at 30 ± 0.5 mN/m in some experiments as this value corresponds to a lipid packing density theoretically equivalent to that of the outer leaflet of the eukaryotic and prokaryotic cell membrane^55^. In other experiments, the critical pressure of insertion of HG2 or HG4 in the total lipid extracts and pure lipids was measured as follows^14,56^. Briefly, in these experiments, the initial pressure of the lipid monolayer was set up at different values (between 10 and 30 mN/m) and the variation of pressure caused by the injection of the peptide was measured. Critical pressure of insertion was calculated by plotting the variation of surface pressure caused by peptide insertion as a function of the initial surface pressure and corresponds to the theoretical value of initial pressure of lipid monolayer that does not allow the insertion of the peptide, i.e. a variation of pressure equal to 0 mN/m. All experiments were carried out in a controlled atmosphere at 20 ± 1 °C and data were analyzed using the Firmware 2.5 programme (Kibron Inc.). The accuracy of the system under our experimental conditions was determined to be ±0.25 mN/m for surface pressure measurements.

### Transcriptomic analysis of peptide activity

The mechanism of action and effects of HG2 and HG4 against *S. aureus* MRSA USA300 was investigated using transcriptomics analysis^57,58^. Briefly, 5 mL exponential-phase cultures of *S. aureus* strain MRSA USA300 (OD_600nm_ ~0.4) grown in MHB were treated separately with either HG2 and HG4 at their MIC concentration (16 and 32 µg/ml) for 1 h to determine differentially expressed genes in the presence or absence of the peptides. The increased sample volume and limited treatment time of 60 min served to maintain the quality and yield of RNA. Untreated cells served as control. Cells were washed and resuspended in PBS and then RNAprotect Bacteria Reagent (QIAGEN, Hilden, Germany) was added and pelleted. This was followed by incubation in Tris-EDTA buffer containing Proteinase K and Bacterial Lysis Mix (Mutanolysin and Lysozyme) at room temperature for 10 min addition. Thereafter, RNA extraction was performed using the Qiagen RNeasy Plus Mini Kit following the manufacturer’s protocol (Qiagen, Manchester, UK). RNA was quantified using a Qubit™ fluorometer with a broad range RNA kit (Invitrogen, USA), according to the manufacturer’s instructions. All experiments were carried out in triplicates. Next, RNA enrichment was performed by ribosomal RNA depletion using the MICROBExpressTM kit (Thermo Fisher) as per the manufacturer’s guidelines. Enriched mRNA was then used to prepare dual-indexed sequencing libraries with the Illumina® TruSeq® Stranded mRNA Sample Preparation Kit following the Illumina protocol (poly-A enrichment step was omitted) and sequencing in 2 × 75 or 2 × 125 bp format on an Illumina Nextseq 500 (HG2 treatment group and control samples) and Hiseq 2500 platforms (HG4 treatment group and control samples) based on accessibility at the time of the experiment.

Analysis of sequencing data was carried out using the Geneious Prime® (2021.1.1) software (Biomatters Ltd, New Zealand)^59^. Firstly, all raw paired-end fastq reads passing quality check by FastQC v0.11.9 were mapped to the reference genome (*Staphylococcus aureus* subsp. aureus USA300_TCH1516 downloaded from the NCBI website), using Geneious RNA-Seq mapper. All generated data have been deposited in the European Nucleotide Archive under study number PRJEB41423. The ‘calculate expression level tool’ was used to calculate the raw, fragment per kilobase per million counts, transcript counts and their normalised versions Reads per kilobase of transcript per million reads mapped (RPKM), Fragments per kilobase of transcript per million fragments mapped (FPKM) and transcript per million reads (TPM) respectively. The raw count matrix obtained from Geneious software was then loaded into the R environment^54^ and data normalised and transformed to a log10 scale. All functions were run using the default parameters unless stated otherwise. The raw script file used for the analysis can be found at https://github.com/brandonyph/SaureusRNASeq/. The Gene Ontology (GO) information for each gene was obtained from Uniprot Online Mapping Tools (https://www.uniprot.org/uploadlists/). Each sample was run through Principal Component Analysis (PCA) using the ‘FactoMineR’ and ‘factoextra’ package in R. The analysis was conducted with the pca() function for both the individual genes and the plots were constructed with ‘fviz_pca_ind()’ and ‘fviz_pca_var()’.

After the removal of outlier samples, a hierarchical clustering based on normalised gene expression levels was performed on the rows and columns using the 'pheatmap' function in the pheatmap library and the heatmap was constructed on each sample using the same function.

Samples that appear to be outliers in both PCA and hierarchical clustering analysis were removed from the differentially expressed genes (DEGs) analysis step. DEGs for each sample was selected using the DESeq2 algorithm in the Geneious software, with a *p* value cut-off of <0.05, and a log2 fold change of abs(log2FC)> 2. The total frequencies of each GO ID in the DEGs list were then summarised using the 'table()' function and visualised with the 'ggplot library' in R.

### Human cell culture and cytotoxicity studies

The toxicity of HG2 and HG4 was tested by a well-known method^60^ using the following cell types: BEAS-2B (normal airway epithelial cells, ATCC CRL-9609), IMR-90 (normal fibroblasts, ATCC CCL-186) and HepG2 (liver cell line, ATCC HB-8065). BEAS-2B, IMR-90 and HepG2 cells were cultured in Dulbecco’s modified essential medium (DMEM) supplemented with 10% fetal calf serum (FCS), 1% l-glutamine and 1% antibiotics (all from Invitrogen). Cells were routinely grown onto 25 cm^2^ flasks maintained in a 5% CO_2_ incubator at 37 °C. Briefly, cells grown on 25 cm^2^ flasks were detached using trypsin-EDTA solution (Thermofisher) and seeded into 96-well cell culture plates (Greiner Bio-one) at ~10^4^ cells per well (counted using Mallasez’s chamber). The cells were grown at 37 °C in a 5% CO_2_ incubator until they reached confluence (~48–72 h of seeding). Wells were then aspirated and increasing concentrations of HG2 or HG4 were added to the cells and incubated for a further 48 h at 37 °C in a 5% CO_2_ incubator. The wells were then emptied, and cell viability was evaluated using a resazurin based in vitro toxicity assay kit (Sigma-Aldrich) following the manufacturer’s instructions. Briefly, the resazurin stock solution was diluted at 1:100 in sterile PBS containing calcium and magnesium (PBS^++^, pH 7.4) and emptied wells were filled with 100 µl of the diluted resazurin solution. After 4 h incubation at 37 °C with the peptide-treated cells, fluorescence intensity was measured using a microplate reader (excitation wavelength of 530 nm/emission wavelength of 590 nm). The fluorescence values were normalised by the controls and expressed as the percentage of cell viability. The IC_50_ values of HG2 or HG4 on cell viability (i.e. the concentration of peptides causing a reduction of 50% of the cell viability) were calculated using GraphPad® Prism 7 software.

### Peptide anti-inflammatory activity

The inhibitory activity of HG2, HG4 and comparator compounds against LPS and LTA was assessed using eLUCidate™ Raw 264.7 NF-kB reporter cell line (AMSBIO) as previously described^42^. The eLUCidate NF-kB reporter cell line is a stably transfected Raw 264.7 cell line which expresses the Renilla luciferase reporter gene under the transcriptional control of the NF-kB response element, a key transcription factor involved in immune and inflammatory responses. This reporter cell line has been used to measure the induction of various toll-like receptors (TLR) by ligands, including LPS and LTA, therefore providing an effective way to evaluate inhibitors of their activations. Briefly, cells were grown and maintained following the manufacturer’s instructions in Dulbecco’s modified essential medium (DMEM) supplemented with 10% fetal calf serum (FCS), 1% l-glutamine, 1% antibiotics (Invitrogen) and containing 3 µg/ml of puromycin (Sigma-Aldrich, UK). To test the inhibitory activity of HG2 and HG4 on LPS or LTA-driven NF-kB activation, eLUCidate™ Raw 264.7 cells grown on 75 cm^2^ flasks were detached using trypsin-EDTA solution (Thermofisher, UK) and seeded into 96-well cell culture plates (Greiner Bio-one, UK) at ~25,000 cells per well (counted using Mallasez’s chamber). The next day, wells were emptied, and cells were exposed to optimal concentrations of LPS or LTA determined from preliminary experiments, (i.e. 10 ng/ml of LPS from *E. coli* or 10 µg/ml of LTA from *S. aureus* (Invivogen, UK)) in the presence of increasing concentrations of HG2 or HG4 (from 0 to 500 µg/ml, serial 1:2 dilutions). Pyrrolidine dithiocarbamate (PDTC, 100 µM) and polymyxin B (PMB, 100 µM) (Sigma-Aldrich, UK) were used as positive controls based on their well-known activity as anti-inflammatory molecules and blockers/neutralisers of LPS and LTA, respectively. Importantly, all steps were performed using non-pyrogenic plastics and RNase/DNase molecular biology tips to limit the risk of the presence of a trace of LPS/LTA. After 6 h incubation at 37 °C and 5% CO_2_, wells were emptied, and the cells were treated with 70 µl of ice-cold PBS containing 1% Triton X-100. After 10 min incubation on ice under orbital shaking at 200 rpm, 50 µL of cell lysates were collected and transferred into white 96-well luminescence plates (Dominique Dutscher) already containing 100 µL of Renilla luciferase substrate (Yelen, France). Luminescence signals of the wells were immediately measured using a microplate reader (Biotek Synergy Mx, UK). The luminescence values were expressed as a percentage of control inflammation corresponding to values obtained after treatment with LPS or LTA in the absence of an inhibitor. The IC_50_ values of PDTC, PMB, HG2 or HG4 on LPS or LTA-mediated inflammation (i.e. the concentration of molecule causing a reduction of 50% of the luciferase’s induction caused by LPS or LTA) were calculated using GraphPad® Prism 7 software.

### In vivo efficacy in *Galleriamellonella* model

All procedures of larvae rearing, injection and *G. mellonella* killing assays were conducted using validated methods^44^. In each assay, ten larvae weighing between 280–300 mg each were randomly selected. Larvae with the previous melanisation of the cuticle were excluded from the experiments. All experiments were designed in at least four experimental and biological replicates.

Firstly, we evaluated the putative toxic effect of the peptides HG2 and HG4 in *G. mellonella*. Each peptide solution prepared in sterile H_2_O was injected in larvae at the concentration of 1x MIC, as 16 mg/kg of larvae body weight (LBW) for HG2 and 32 mg/kg LBW for HG4; and 3x MIC 64 mg/kg LBW HG2 and 98 mg/kg LBW HG4, as previously determined. After injection with peptides, the larvae were maintained at 37 °C in the dark. Phenotypic aspects such as melanisation and mobility of the larvae as well as survival were monitored every 24 for 96 h. Larvae not inoculated with AMPs were used as controls.

To determine the LD_50_ and LD of *S. aureus* MRSA USA300 in *G. mellonella*, an inoculum of 10 µl of MRSA USA300 suspension in PBS 1X (10^3^ to 10^6^ CFU/larvae) was injected into the larvae haemocoel using insulin syringes (Becton Dickinson, USA). Larvae inoculated with PBS and larvae not inoculated were used as negative controls. After the injections, the larvae were maintained at 37 °C in the dark, the survival was recorded every 24 for 96 h and the LD_50_ and LD were determined.

The efficacy of the peptides HG2 and HG4 to control MRSA USA300 infection in vivo, was evaluated following the protocol by ref. ^43^. The groups of *G. mellonella* larvae were infected with the predetermined lethal dose of MRSA USA300. After 30 min of the larvae injection with the bacteria, the peptides were injected at 1x MIC and 3x MIC concentrations respectively. Larvae injected with PBS solution, and HG2 or HG4 peptide solution (1x MIC or 3x MIC) alone were used as negative controls. Injected larvae were maintained at 37 °C in the dark and their survival was monitored and analyzed as above.

### Statistical analysis

All biological experiments were repeated at least three times and three biological replicates were used wherever applicable. Results are expressed as mean ± standard error. The MRSA USA300 LD_50_ value was calculated by linear regression using software R v.4.11^54^. The Kaplan–Meier method was used to plot the survival curves of *G. mellonella*. Differences in survival were calculated using the log-rank test with the software SigmaPlot (Systat Software Inc., CA, USA). A *p* value of 0.05 was considered to be statistically significant.

## Supplementary information

Supplementary Information

## Data Availability

All data generated or analysed during this study are included in this published article and its Supplementary Information file. The transcriptomic datasets generated and analysed during the study are available under study number PRJEB41423 in the European Nucleotide Archive. The raw script file used for the transcriptomic analysis can be found at https://github.com/brandonyph/SaureusRNASeq/.
