# Supplementary material for: In silico identification of two peptides with antibacterial activity against multidrug-resistant Staphylococcus aureus
Source: NPJ Biofilms Microbiomes. 2022 Jul 14;8:58. doi: 10.1038/s41522-022-00320-0 (PMC9283466; doi:10.1038/s41522-022-00320-0)
Supplement: Supplementary file 1 — Supplementary Information [file 41522_2022_320_MOESM1_ESM.pdf]

Supplementary Information for

In silico identification of two novel peptides with antibacterial activity against multidrug resistant *Staphylococcus aureus*

Linda B Oyama, Hamza Olleik, Ana Carolina Nery Teixeira, Matheus M Guidini, James A Pickup, Brandon Yeo Pei Hui, Nicolas Vidal, Alan R Cookson, Hannah Vallin, Toby Wilkinson, Denise M S Bazzolli, Jennifer Richards, Mandy Wootton, Ralf Mikut, Kai Hilpert, Marc Maresca, Josette Perrier, Matthias Hess, Hilario C Mantovani, Narcis Fernandez-Fuentes, Christopher J Creevey, and Sharon A Huws.

Corresponding authors: Dr Linda B Oyama (l.oyama@qub.ac.uk) and Dr Sharon A Huws (s.huws@qub.ac.uk)

This PDF file includes:

Supplementary Figures 1 to 3
Supplementary Tables 1 to 11

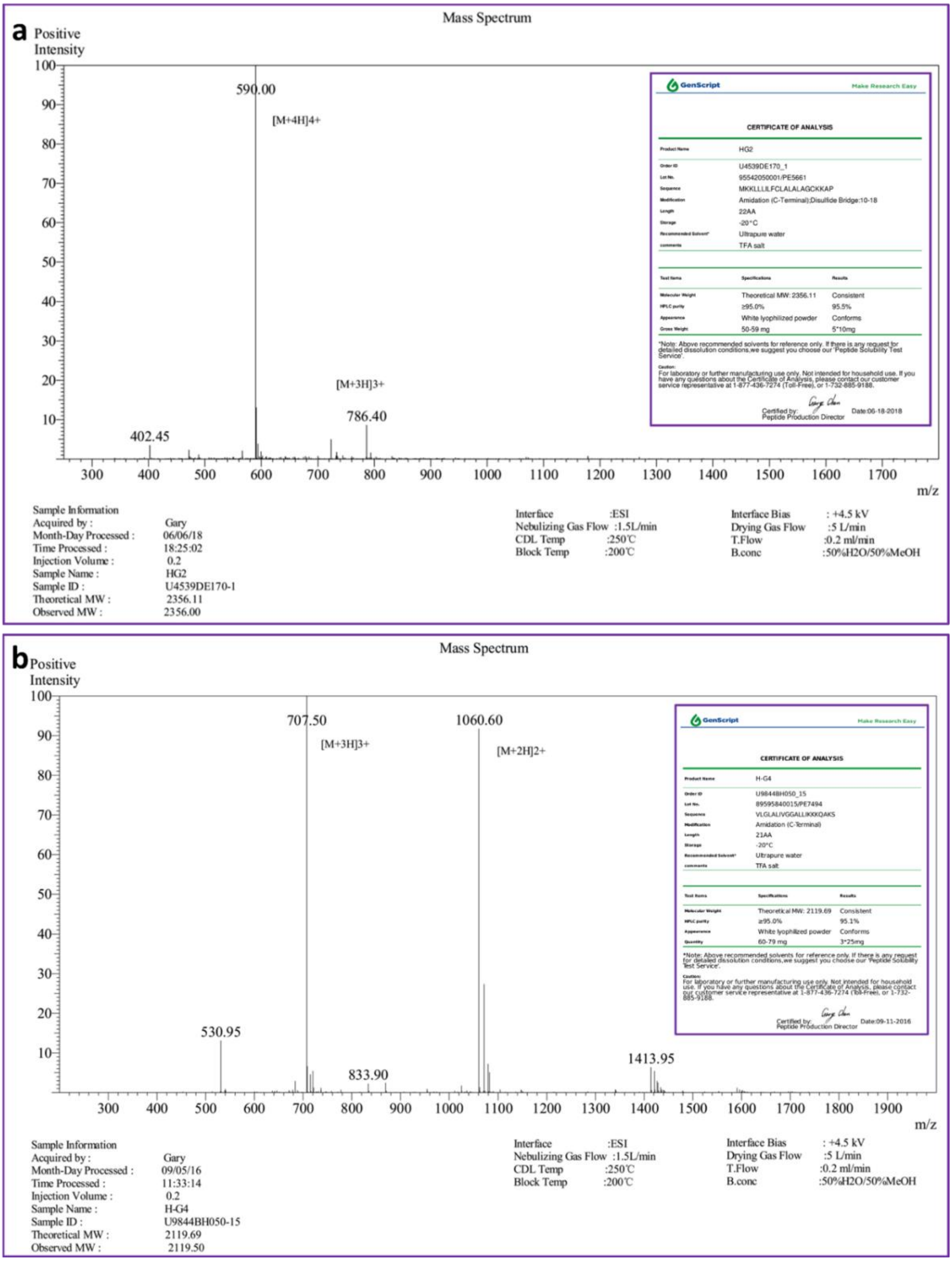

Supplementary Figure 1. Mass spectrometry and certificate of analysis (COA) report for AMP chemical synthesis: a) HG2 and b) HG4. Peptides were synthesised by GenScript Inc. USA. Mass spectrum analysis and COA report show correct molecular weight and purity grade for both peptides.

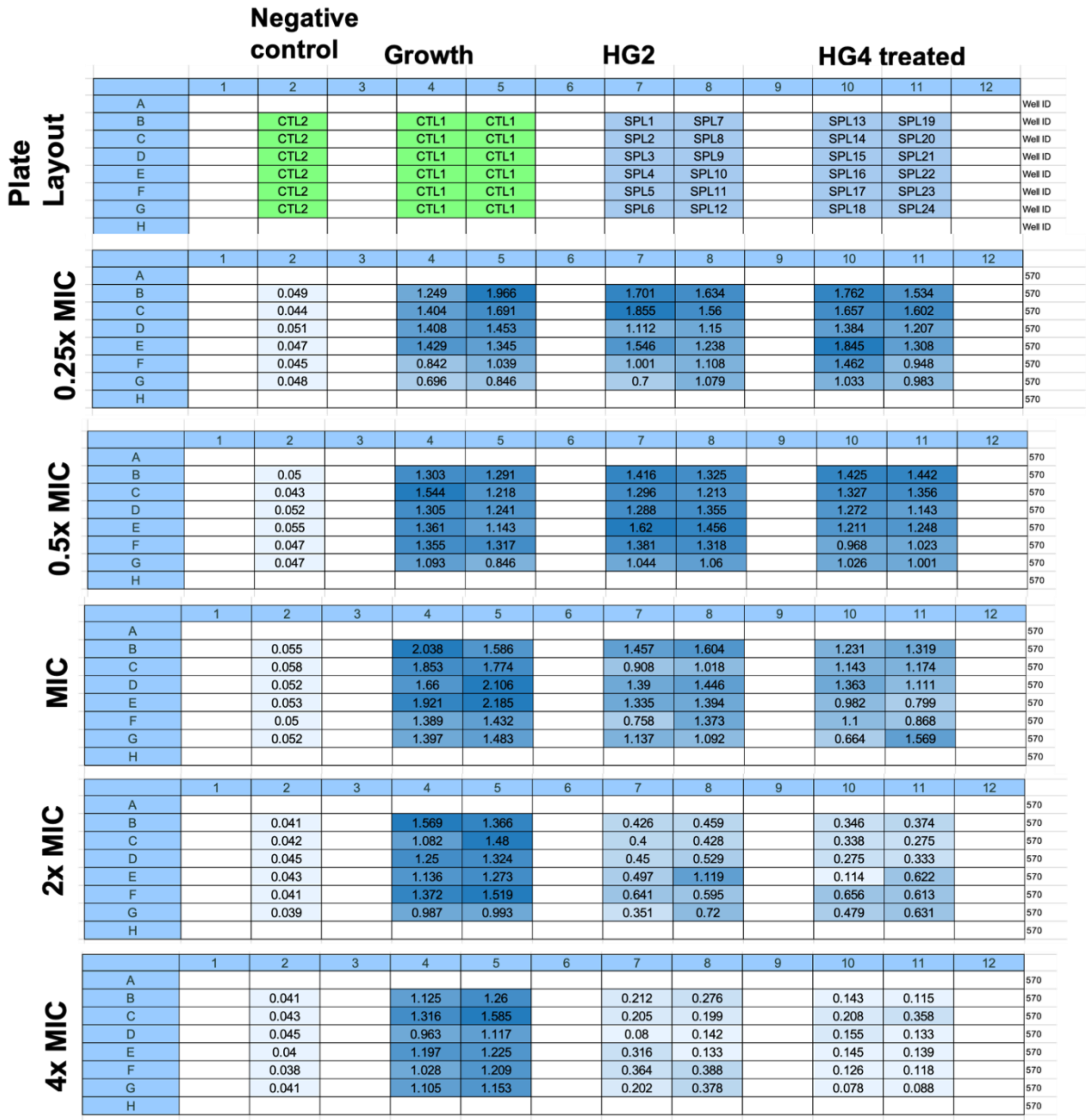

Plate Layout

	1	2 (Negative control)	3	4 (Growth)	5 (Growth)	6	7 (HG2)	8 (HG2)	9	10 (HG4 treated)	11 (HG4 treated)	12	
A													Well ID
B		CTL2		CTL1	CTL1		SPL1	SPL7		SPL13	SPL19		Well ID
C		CTL2		CTL1	CTL1		SPL2	SPL8		SPL14	SPL20		Well ID
D		CTL2		CTL1	CTL1		SPL3	SPL9		SPL15	SPL21		Well ID
E		CTL2		CTL1	CTL1		SPL4	SPL10		SPL16	SPL22		Well ID
F		CTL2		CTL1	CTL1		SPL5	SPL11		SPL17	SPL23		Well ID
G		CTL2		CTL1	CTL1		SPL6	SPL12		SPL18	SPL24		Well ID
H													Well ID

0.25x MIC

	1	2	3	4	5	6	7	8	9	10	11	12	
A													570
B		0.049		1.249	1.966		1.701	1.634		1.762	1.534		570
C		0.044		1.404	1.691		1.855	1.56		1.657	1.602		570
D		0.051		1.408	1.453		1.112	1.15		1.384	1.207		570
E		0.047		1.429	1.345		1.546	1.238		1.845	1.308		570
F		0.045		0.842	1.039		1.001	1.108		1.462	0.948		570
G		0.048		0.696	0.846		0.7	1.079		1.033	0.983		570
H													570

0.5x MIC

	1	2	3	4	5	6	7	8	9	10	11	12	
A													570
B		0.05		1.303	1.291		1.416	1.325		1.425	1.442		570
C		0.043		1.544	1.218		1.296	1.213		1.327	1.356		570
D		0.052		1.305	1.241		1.288	1.355		1.272	1.143		570
E		0.055		1.361	1.143		1.62	1.456		1.211	1.248		570
F		0.047		1.355	1.317		1.381	1.318		0.968	1.023		570
G		0.047		1.093	0.846		1.044	1.06		1.026	1.001		570
H													570

MIC

	1	2	3	4	5	6	7	8	9	10	11	12	
A													570
B		0.055		2.038	1.586		1.457	1.604		1.231	1.319		570
C		0.058		1.853	1.774		0.908	1.018		1.143	1.174		570
D		0.052		1.66	2.106		1.39	1.446		1.363	1.111		570
E		0.053		1.921	2.185		1.335	1.394		0.982	0.799		570
F		0.05		1.389	1.432		0.758	1.373		1.1	0.868		570
G		0.052		1.397	1.483		1.137	1.092		0.664	1.569		570
H													570

2x MIC

	1	2	3	4	5	6	7	8	9	10	11	12	
A													570
B		0.041		1.569	1.366		0.426	0.459		0.346	0.374		570
C		0.042		1.082	1.48		0.4	0.428		0.338	0.275		570
D		0.045		1.25	1.324		0.45	0.529		0.275	0.333		570
E		0.043		1.136	1.273		0.497	1.119		0.114	0.622		570
F		0.041		1.372	1.519		0.641	0.595		0.656	0.613		570
G		0.039		0.987	0.993		0.351	0.72		0.479	0.631		570
H													570

4x MIC

	1	2	3	4	5	6	7	8	9	10	11	12	
A													570
B		0.041		1.125	1.26		0.212	0.276		0.143	0.115		570
C		0.043		1.316	1.585		0.205	0.199		0.208	0.358		570
D		0.045		0.963	1.117		0.08	0.142		0.155	0.133		570
E		0.04		1.197	1.225		0.316	0.133		0.145	0.139		570
F		0.038		1.028	1.209		0.364	0.388		0.126	0.118		570
G		0.041		1.105	1.153		0.202	0.378		0.078	0.088		570
H													570

Supplementary Figure 2. AMP anti-biofilm activity against MRSA USA300 assay plate output in heatmap format: Showing assay plate layout and representative images at the five AMP concentrations tested. Intensity of biofilms ranging from dark blue in the untreated samples (growth control) to very light blue in the HG2 and HG4 treated and samples (at supra-MIC concentrations) and negative controls containing sterile MH broth only.

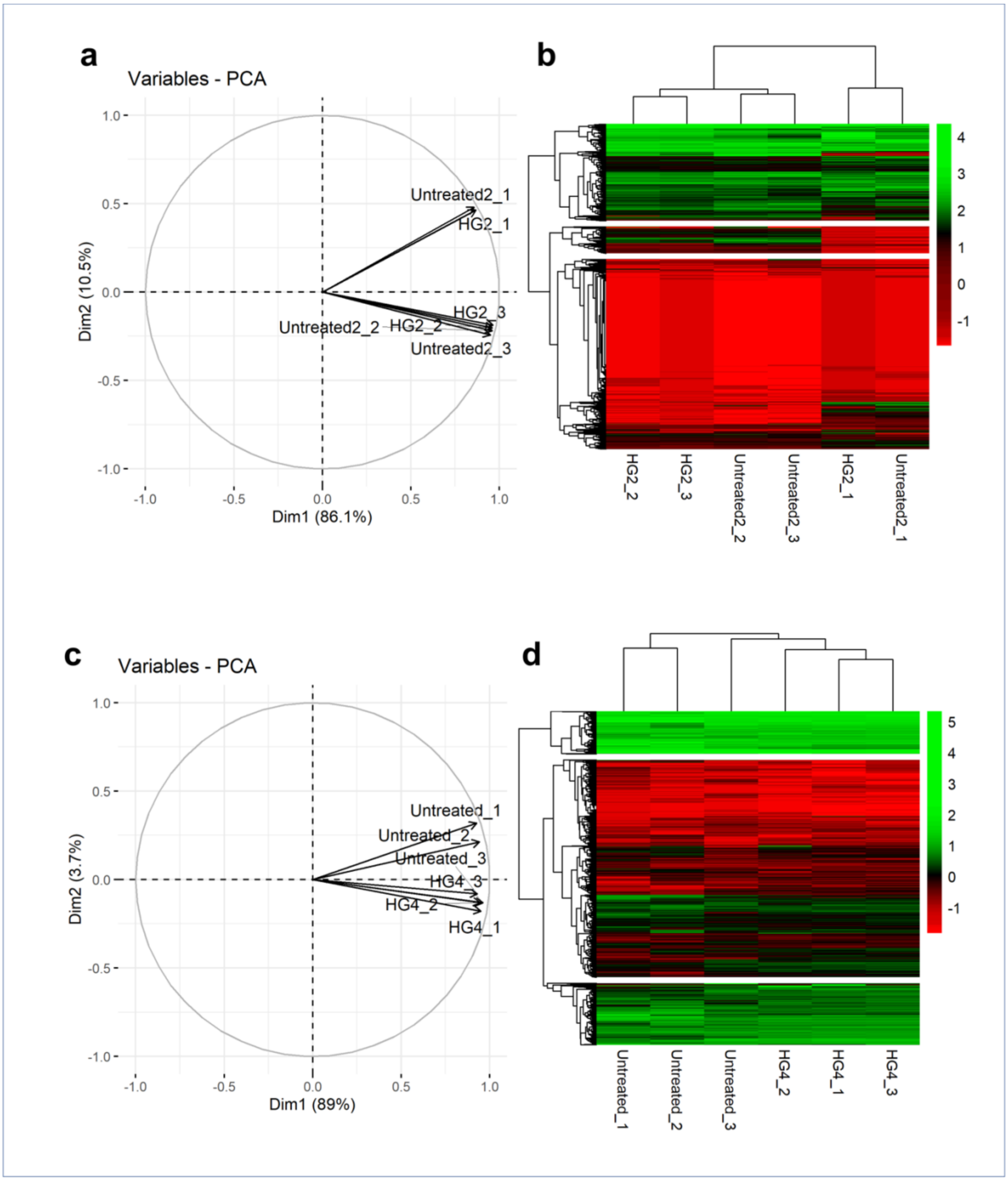

Supplementary Figure 3. Transcriptomic analysis of peptide activity MRSA USA300: a) Principal component plot and **b**) Hierarchical Clustering analysis plot before outlier removal in HG2 treated group. **c)** Principal component plot and **d**) Hierarchical Clustering analysis plot before outlier removal HG4 treated group. HG2_1 and Untreated2_1 being group/treatment outliers and Untreated_3 is an outlier in the HG4 treatment group. PCA plot on samples, showing clustering results of similar display patterns of Untreated_3. Heatmap displaying hierarchical clustering of samples and gene expression, also displaying similar expression patterns of Untreated_3 as compared to the rest.

Supplementary Table 1. Summary of results from computation steps used to identify novel AMPs from the Hess et al, 2011 cow rumen metagenome dataset.

Fusion project	First sequence number	Number of sequences	Number of sequences after first filtering	Candidates fulfilling distance criteria
**0a**	1	100000	35027	32
**0b**	100001	100000	33247	33
**1a**	200001	100000	32561	27
**1b**	300001	100000	37820	37
**2a**	400001	100000	39000	32
**2b**	500001	100000	38806	42
**3a**	600001	100000	32499	35
**3b**	700001	100000	28537	19
**4a**	800001	100000	28701	29
**4b**	900001	100000	33811	11
**5a**	1000001	100000	34135	35
**5b**	1100001	100000	38599	42
**6a**	1200001	100000	31472	29
**6b**	1300001	100000	35397	21
**7a**	1400001	100000	38893	26
**7b**	1500001	100000	40084	30
**8a**	1600001	100000	40459	40
**8b**	1700001	100000	40718	41
**9a**	1800001	100000	40593	22
**9b**	1900001	100000	40600	23
**10a**	2000001	100000	40460	36
**10b**	2100001	100000	40688	46
**11a**	2200001	100000	34674	41
**11b**	2300001	100000	31152	31
**12a**	2400001	100000	33339	36
**12b**	2500001	47270	16364	33
**SUM**		**2547270**	**917636**	**829**

Supplementary Table 2. Six most promising antimicrobial peptide candidates (HG1-HG6) identified in cow rumen metagenome dataset

Assigned name	Sequence	Amino acid (AA) (length)	Location on cow dataset	Most similar homolog on APD2 (stop codon '*' removed)	
				APD ID	Similarity %
**HG1**	VKKAPAKKAAPKAAAKENVKAAAAPATKSTKKPKKV	37	NODE_3603970_length_237431_cov_15.909123_orf_36410 39090..39200	AP00537	45%
**HG2**	MKKLLLILFCLALALAGCKKAP	22	NODE_664976_length_19740_cov_2.033485_orf_00810 19724..19789	AP00494	40%
**HG3**	LLFLALSLLFQLLFPLFLFAFEQQALPFLFLLPLFLLASFQLPLSLFFQLLL	53	NODE_4144838_length_4700_cov_0.195319_orf_38300 3..161	AP01999	30.76%
**HG4**	VLGLALIVGGALLIKKKQAKS	21	NODE_3958153_length_85376_cov_8.525382_orf_203250 82784..82849	AP01737	48%
**HG5**	LFGGGGGTGGFGLGGPGGNGPGGGGGPGGNVPGS	35	NODE_793869_length_3085_cov_43.351379_orf_34140 complement(2443..2547)	AP02183	52.50%
**HG6**	MLSFLFMQKLMKKKLKRRKKLKLKRLLLKKKLLKKQLKRFLKRKFL	47	NODE_2576652_length_4557_cov_3.402019_orf_04080 350..490	AP01010	36.17%

Supplementary Table 3. Scaffold nucleotide sequences from which putative antimicrobials H-G1- H-G6 were identified

Assigned name	Nucleotide sequence on scaffold from which genes have been identified<br>(the highlighted are the regions of interest)	Position on scaffold/orientation	Corresponding Protein sequence	Notes
H-G1	ATGGGATTTCGTGAATCATACAGGCGAGAT<br>TCACGACTGGAACAGCCACGGTACTCACTG<br>CGCAGGTATAGCAGCCGCCNNNNNNNNNN<br>GGCCGAGAAGAAAGAG**GTGAAGAAAGCC**<br>**CCGGCAAAGAAAGCCGCACCTAAGGCAG**<br>**CAGCTAAGGAAAACGTGAAGGCTGCAGC**<br>**AGCTCCGGCTACAAAGTCGACAAAGAAG**<br>**CCGAAGAAAGTGTAG**	39090..39200<br>/5’-3’	VKKAPA<br>KKAAPK<br>AAAKEN<br>VKAAA<br>APATKS<br>TKKPKK<br>V	part of a larger gene but contains a start and stop codon
H-G2	TCAAGCATCAACATGCTGGAAGCCAGCGCC<br>CTCATGAGCGTCGGCAAGATCGACGAGGCG<br>GAAAGCAAGCTTGACGCCGTCTTCGGGGAC<br>GCCAACACCACCGTCGACCAGAAGCAGAG<br>GATCTTGCTTACCAAGGCCATGCTCGCCCT<br>GCGCGCCAAAGGCGACTCCGAGAGCTTCGA<br>GAAGCTGACGGAAGAAGCCATCGCCCTGG<br>ATCCCGAAAGCGAGCTGAGCAAGCAGGCT<br>ACCGTCCAGCTGGAGACGGTCAAGAACATG<br>ATCAAGGAAAGCAAGCAGGAGCAGGAAAA<br>GGAAGTCGAGCAGGAAGTCAGCGAGGAAC<br>AGGAAGAAGCCATAAAGGAACCGGCCGAA<br>TAAGCCGAATACTGTCAGGTCCCAAGC**ATG**<br>**AAAAAACTTTTGCTCATTCTTTTCTGCCT**<br>**CGCCCTGGCCCTGGCCGGCTGCAAAAAG**<br>**GCCCCG**	19724..19789/3'-5'	MKKLLL<br>ILFCLAL<br>ALAGCK<br>KAP	on complementary strand of scaffold sequence
H-G3	**CTTCTCTTTCTTGCTCTTTCTTTGCTTTTT**<br>**CAGCTTCTTTTTCCTCTTTTTCTTTTTGCT**<br>**TTTGAGCAGCAAGCTCTGCCTTTTCTCTT**<br>**TCTTTTGCCTCTTTTTCTGCTTGCTTCTTT**<br>**TCAGCTTCCTCTCTCT**<br>**TTATTCTTTCAGCTTCTTCTCTAG**	38300...3..161/<br>5’-3’	LLFLALS<br>LLFQLLF<br>PLFLFAF<br>EQQALP<br>FLFLLPL<br>FLLASF<br>QLPLSLF<br>FQLLL	No start codon
H-G4	ATGGACCATCCCTGCCGACCAGAAGCAGCA<br>TGCCCGNNNNNNNNNNNNNNNNNNNNNNNN<br>NNNNNNNNNNNNNNNNNNNNNNNNNNNNN<br>NNNNNNNNNNNNNNNNNNNNNNNNNNNNN<br>NNNNNNNNNNNNNNNNNNNNNNNNNNNNN<br>NNNNNNNNNNNNNNNNNNNNNNNNNNNNN<br>NNNNNNNNNNNNNNNNNNNNNNNNNNNGG<br>GCGTGGCGACGGTCGCGCATACCTTCCAGG<br>AAGCTTTGAAGGGTGGCAAGACGAAGGTG<br>CTCGATACGCGT AAGACGGTTCCCGGTTTC<br>CGCACGCTGCAGAAGTATGCGGTACGT GT<br>GGGCGGCGGATCCAACCACCGTATGGGCCT<br>TTTCGACATGGTG CTTGTGAAGGACAATC<br>ACATTGCTGCGGCGGGTGGCGTGCTCCAG<br>GCGCTCGAAGTGGTGAAGAAGAACAACAA<br>GCAGAACCTGATNNNNNNNNNNNNNNNNNN<br>NNNNNNNNNNNNNNNNNNNNNNNNNNNNN<br>NNNNNNNNNNNNNNNNNNNNNNNNNNNNN<br>NNNNNNNNNNNNNNNNNNNNNNNNNNNNN<br>NNNNNNNNNNNNNNNNNNNNNNNNNNNNN<br>NNNNNNNNNNNNNNNNNNNNNNNNNNNNN<br>NNNNNNNNNNNNNNNNNNNNNNNNNNNNN<br>NNNNNNNNNNNNNNNNNNNNNNNNNNNNN<br>NNNNNNNNNNNNNNNNNNNNNNNNNNNNN<br>NNNNNNNNNNNNNNNNNNNNNNNNNNNNN<br>NNNNNNNNNNNNNNNNNNNNNNNNNNNNN<br>NNNNNNNNNNNNNNNNNNNNNNNNNNNNN	82784-82849/5’-3’	VLGLAL<br>IVGGAL<br>LIKKKQ<br>AKS	part of a larger gene but contains a start and stop codon

	NNNNNNNNNNNNNNNNNNNNNNNNNNNNNN<br>NNNNNTTGGGTCGTCGTGTCTTGAAGTTGC<br>GCAAGTGCCTGGAATGTTTGCCAGAAGGTT<br>TNNNNNNNNNNNT**GTGCTTGGCCTTGCACT**<br>**GATTGTCGGTGGAGCTTTGCTTATCAAG**<br>**AAGAAACAGGCGAAATCTTAATTTGCGC**<br>**CATCACGATGGCGACCATCATAATNNNN**<br>**NNNNNNAAGCGCGCTTCTTTCGTTCGTGC**<br>**TTGGCCTTGTGCTGATTGTCGGTGGAGC**<br>**CTTGCTCATCAAGAA**GAGGCAGGCGAAAT<br>CTTAATTTGCGCCATCACGATGGCGACCAT<br>CATAATGGCACAACCGCAAATCTCGCGGGC<br>TGAAAGCTGTTCTCCTAGAATGGCCCAACC<br>CGCAAGGACGGCGAATACCGATTCCAGACT<br>CATCGTGAGTGAGGCGATGGTGGGGTTGAT<br>TTTATCT			
H-G5	ATGATATCGTTAAATATCTTTACGCTTCTTT<br>GACGCTTATATACTTCCATAATACCGCGAA<br>CAGGATGTCTAGGTGTAGGCCAAATACTTA<br>ATTCTTTTTCCTTATCGTTATATCTTACCTGA<br>TATGCTTCACCGAAATCAAGCTTTGCTTGTT<br>CAAGCCACATAAGAGCTGCATTCCAGTTAC<br>CGAGAACATCACCGAAACCTGCAGAGTTTC<br>CATAGCATGCGCCACGGAATATACTGGAAG<br>TATTCATACTCATGACGGAATCATACAATG<br>CATTATGCGGCAATGTGAAGAGTTCATTGA<br>TATCGCCAATCCAGTTTGCGGTCTGGAAAT<br>CGATAACTTCTTCAAGTTCTTGGCAAATCTT<br>ATAATGCGTCCTGCCAGGTTGCAATTCCATT<br>ACAAGATAGTCACGATAGTTACCCTGTCGC<br>GCATAATATCTCTGCACATATCGAATCATG<br>TCGGCGATGATGTAATTTAATTGTTCGTCAC<br>TGATTTCAATACAGATTACAGGAGCGCCGA<br>GCATTAACTTTATATAGTTACGCATTTGCGC<br>CATATTCTGTATCATCGTAGATTCGAGTACA<br>GGGTCGCAA**TTGTTCGGCGGCGGAGGTG**<br>**GAACAGGCGGTTTTGGCCTTGGCGGACC**<br>**CGGTGGAAACGGACCCGGCGGAGGAGG**<br>**CGGTCCTGGCGGAAATGTGCCTGGTTCA**<br>**TAG**CCCGGAGGAGGCGGTTTCAATCGTGAC	2443-2547/3'-5'	LFGGGG<br>GTGGFG<br>LGGPGG<br>NGPGGG<br>GGPGGN<br>VPGS	on<br>complementar<br>y strand of<br>scaffold (full)

H-G6	CTTGAACCGGTCGGCGGCATTATAGGATCA<br>CGCATAGGCATTAAATTATCGTCAATTACA<br>TCACTCAT<br><br>**ATGCTATCATTCCTATTCATGCAGAAGCT**<br>**GATGAAGAAGAAGTTGAAGCGGAGGAAG**<br>**AAGTTGAAGCTGAAGAGGCTCCTGTTGA**<br>**AGAAGAAGCTGTTGAAGAAGCAGTTGAA**<br>**GAGATTCCTGAAGAGGAAGTTTCTTTAG**	350-490/5’-3’	MLSFLF<br>MQKLM<br>KKKLKR<br>RKKLKL<br>KRLLLK<br>KKLLKK<br>QLKRFL<br>KRKFL	full gene

Supplementary Table 4. eggNOG mapper Annonations for HG2 and HG4 containing contigs

<table>
<tr><th>query</th><th>seed_ortholog</th><th>evalue</th><th>score</th><th>eggNOG_OGs</th><th>max_annot_lvl</th><th>COG_category</th><th>Description</th><th>Preferred _name</th><th>GOs</th><th>EC</th><th>KEGG_ko</th></tr>
<tr><td></td><td>1089553.Tph_c17290</td><td>8.88e-57</td><td>195.0</td><td>COG0482@1|root,COG0482@2|Bacteria, 1TPIZ@1239|Firmicutes, 247YV@186801|Clostridia, 42F6N@68295|Thermoanaerobacterales</td><td>186801|Clostridia</td><td>J - Translation, ribosomal structure and biogenesis</td><td>Catalyzes the 2-thiolation of uridine at the wobble position (U34) of tRNA, leading to the formation of s(2)U34</td><td>mnmA</td><td>-</td><td>2.8.1.13</td><td>ko:K00566</td></tr>
<tr><td></td><td>1121422.AUMW0100003</td><td>7.69e-60</td><td>196.0</td><td>COG0413@1|root,COG0413@2|Bacteria, 1TPZA@1239|Firmicutes, 248RR@186801|Clostridia, 260CY@186807|Peptococcaceae</td><td>186801|Clostridia</td><td>H - Coenzyme transport and metabolism</td><td>Catalyzes the reversible reaction in which hydroxymethyl group from 5,10-methylenetetrahydrofolate is transferred onto alpha-ketoisovalerate to form ketopantoate</td><td>panB</td><td>-</td><td>2.1.2.11</td><td>ko:K00606</td></tr>
<tr><td></td><td>1038860.AXAP01000029_</td><td>5.84e-50</td><td>176.0</td><td>COG0166@1|root,COG0166@2|Bacteria, 1MUFP@1224|Proteobacteria, 2TQKP@28211|Alphaproteobacteria, 3JWSI@41294|Bradyrhizobiaceae</td><td>28211|Alphaproteobacteria</td><td>G- Carbohydrate transport and metabolism</td><td>Phosphoglucose isomerase</td><td>pgi</td><td>GO:0005575,GO:0005622,GO:0005623,GO:0005737,GO: 0044424,GO:0044464</td><td>5.3.1.9</td><td>ko:K01810</td></tr>
<tr><td></td><td>1122915.AUGY01000003_</td><td>5.28e-30</td><td>113.0</td><td>COG2148@1|root,COG2148@2|Bacteria, 1TP7M@1239|Firmicutes, 4HCBG@91061|Bacilli, 26QNZ@186822|Paenibacillaceae</td><td>91061|Bacilli</td><td>M - Cell wall/membrane/envelope biogenesis</td><td>Multidrug MFS transporter</td><td>cpsE</td><td>GO:0005575,GO:0005623,GO:0005886,GO:0016020,GO: 0044464,GO:0071944</td><td>-</td><td>ko:K13012,ko:K19428</td></tr>
<tr><td></td><td>1382358.JHVN01000038_</td><td>3.06e-41</td><td>154.0</td><td>COG0518@1|root,COG0519@1|root, COG0518@2|Bacteria, COG0519@2|Bacteria, 1TPG8@1239|Firmicutes, 4HA7Q@91061|Bacilli, 21VJJ@150247|Anoxybacillus</td><td>91061|Bacilli</td><td>F - Nucleotide transport and metabolism</td><td>Catalyzes the synthesis of GMP from XMP</td><td>guaA</td><td>GO:0003674,GO:0003824,GO:0003921,GO:0003922,GO: 0006139,GO:0006163,GO:0006164,GO:0006177,GO:0006725, GO:0006753,GO:0006793,GO:0006796,GO:0006807,GO: 0008150,GO:0008152,GO:0009058,GO:0009116,GO:0009117, GO:0009119,GO:0009123,GO:0009124,GO:0009126,GO: 0009127,GO:0009150,GO:0009152,GO:0009156,GO:0009161, GO:0009163,GO:0009165,GO:0009167,GO:0009168,GO: 0009259,GO:0009260,GO:0009987,GO:0016874,GO:0016879, GO:0016884,GO:0018130,GO:0019438,GO:0019637,GO: 0019693,GO:0034404,GO:0034641,GO:0034654,GO:0042278, GO:0042451,GO:0042455,GO:0044237,GO:0044238,GO: 0044249,GO:0044271,GO:0044281,GO:0044283,GO:0046037, GO:0046128,GO:0046129,GO:0046390,GO:0046483,GO: 0055086,GO:0071704,GO:0072521,GO:0072522,GO:0090407, GO:1901068,GO:1901070,GO:1901135,GO:1901137,GO: 1901293,GO:1901360,GO:1901362,GO:1901564,GO:1901566, GO:1901576,GO:1901657,GO:1901659</td><td>6.3.5.2</td><td>ko:K01951</td></tr>
<tr><td></td><td>765913.ThidrDRAFT_2517</td><td>1.2e-39</td><td>155.0</td><td>COG1262@1|root,COG1262@2|Bacteria, 1QT7U@1224|Proteobacteria, 1RWY0@1236|Gammaproteobacteria, 1X0ZN@135613|Chromatiales</td><td>135613|Chromatiales</td><td>S - Function unknown</td><td>CDS with the same product name</td><td>-</td><td>-</td><td>-</td><td>-</td></tr>
<tr><td></td><td>1121445.ATUZ01000013_</td><td>1.23e-24</td><td>104.0</td><td>COG1624@1|root,COG1624@2|Bacteria, 1PEQT@1224|Proteobacteria, 42P5A@68525|delta/epsilon subdivisions, 2WKPP@28221|Deltaproteobacteria, 2M84W@213115|Desulfovibrionales</td><td>28221|Deltaproteobacteria</td><td>S - Function unknown</td><td>Catalyzes the condensation of 2 ATP molecules into cyclic di-AMP (c-di-AMP), a second messenger used to regulate differing processes in different bacteria</td><td>dacA</td><td>-</td><td>2.7.7.85</td><td>ko:K18672</td></tr>
<tr><td></td><td>886379.AEWI01000144_g</td><td>6.8e-71</td><td>229.0</td><td>COG2148@1|root,COG2148@2|Bacteria, 4NER4@976|Bacteroidetes, 2FNC2@200643|Bacteroidia, 3XIYM@558415|Marinilabiliaceae</td><td>976|Bacteroidetes</td><td>M - Cell wall/membrane/envelope biogenesis</td><td>CoA-binding domain</td><td>-</td><td>-</td><td>-</td><td>ko:K13012</td></tr>
<tr><td></td><td>1411123.JQNH01000001_</td><td>4.48e-22</td><td>98.6</td><td>COG3842@1|root,COG3842@2|Bacteria, 1MU3I@1224|Proteobacteria, 2TQMJ@28211|Alphaproteobacteria</td><td>28211|Alphaproteobacteria</td><td>P - Inorganic ion transport and metabolism</td><td>Part of the ABC transporter complex PotABCD involved in spermidine putrescine import. Responsible for energy coupling to the transport system</td><td>potA</td><td>GO:0005575,GO:0005623,GO:0005886,GO:0006810,GO: 0006811,GO:0006812,GO:0008150,GO:0015695,GO:0015696, GO:0015846,GO:0015847,GO:0016020,GO:0016021,GO: 0031224,GO:0032991,GO:0043190,GO:0044425,GO:0044459, GO:0044464,GO:0051179,GO:0051234,GO:0071702,GO: 0071705,GO:0071944,GO:0098533,GO:0098796,GO:0098797, GO:1902494,GO:1902495,GO:1904949,GO:1990351</td><td>-</td><td>ko:K11076</td></tr>
<tr><td>HG2 containing contig</td><td>247490.KSU1_D1049</td><td>2.05e-144</td><td>436.0</td><td>COG1178@1|root,COG1178@2|Bacteria, 2J51F@203682|Planctomycetes</td><td>203682|Planctomycetes</td><td>U - Intracellular trafficking, secretion, and vesicular transport</td><td>Binding-protein-dependent transport system inner membrane component</td><td>-</td><td>-</td><td>-</td><td>ko:K02011</td></tr>
<tr><td></td><td>1089553.Tph_c17290</td><td>8.88e-57</td><td>195.0</td><td>COG0482@1|root,COG0482@2|Bacteria, 1TPIZ@1239|Firmicutes, 247YV@186801|Clostridia, 42F6N@68295|Thermoanaerobacterales</td><td>186801|Clostridia</td><td>J - Translation, ribosomal structure and biogenesis</td><td>Catalyzes the 2-thiolation of uridine at the wobble position (U34) of tRNA, leading to the formation of s(2)U34</td><td>mnmA</td><td>-</td><td>2.8.1.13</td><td>ko:K00566</td></tr>
</table>

1121422.AUMW0100003!	7.69e-60	196.0	COG0413@1|root,COG0413@2|Bacteria, 1TPZA@1239|Firmicutes, 248RR@186801|Clostridia, 260CY@186807|Peptococcaceae	186801|Clostridia	**H - Coenzyme transport and metabolism**	Catalyzes the reversible reaction in which hydroxymethyl group from 5,10-methylenetetrahydrofolate is transferred onto alpha-ketoisovalerate to form ketopantoate	panB	-	2.1.2.11	ko:K00606
1038860.AXAP01000029_	5.84e-50	176.0	COG0166@1|root,COG0166@2|Bacteria, 1MUFP@1224|Proteobacteria, 2TQKP@28211|Alphaproteobacteria, 3JWSI@41294|Bradyrhizobiaceae	28211|Alphaproteobacteria	**G- Carbohydrate transport and metabolism**	Phosphoglucose isomerase	pgi	GO:0005575,GO:0005622,GO:0005623,GO:0005737,GO:0044424,GO:0044464	5.3.1.9	ko:K01810
1122915.AUGY01000003_	5.28e-30	113.0	COG2148@1|root,COG2148@2|Bacteria, 1TP7M@1239|Firmicutes, 4HCBG@91061|Bacilli, 26QNZ@186822|Paenibacillaceae	91061|Bacilli	**M - Cell wall/membrane/envelope biogenesis**	Multidrug MFS transporter	cpsE	GO:0005575,GO:0005623,GO:0005886,GO:0016020,GO:0044464,GO:0071944	-	ko:K13012,ko:K19428
1382358.JHVN01000038_	3.06e-41	154.0	COG0518@1|root,COG0519@1|root, COG0518@2|Bacteria, COG0519@2|Bacteria, 1TPG8@1239|Firmicutes, 4HA7Q@91061|Bacilli, 21VJJ@150247|Anoxybacillus	91061|Bacilli	**F - Nucleotide transport and metabolism**	Catalyzes the synthesis of GMP from XMP	guaA	GO:0003674,GO:0003824,GO:0003921,GO:0003922,GO:0006139,GO:0006163,GO:0006164,GO:0006177,GO:0006725,GO:0006753,GO:0006793,GO:0006796,GO:0006807,GO:0008150,GO:0008152,GO:0009058,GO:0009116,GO:0009117,GO:0009119,GO:0009123,GO:0009124,GO:0009126,GO:0009127,GO:0009150,GO:0009152,GO:0009156,GO:0009161,GO:0009163,GO:0009165,GO:0009167,GO:0009168,GO:0009259,GO:0009260,GO:0009987,GO:0016874,GO:0016879,GO:0016884,GO:0018130,GO:0019438,GO:0019637,GO:0019693,GO:0034404,GO:0034641,GO:0034654,GO:0042278,GO:0042451,GO:0042455,GO:0044237,GO:0044238,GO:0044249,GO:0044271,GO:0044281,GO:0044283,GO:0046037,GO:0046128,GO:0046129,GO:0046390,GO:0046483,GO:0055086,GO:0071704,GO:0072521,GO:0072522,GO:0090407,GO:1901068,GO:1901070,GO:1901135,GO:1901137,GO:1901293,GO:1901360,GO:1901362,GO:1901564,GO:1901566,GO:1901576,GO:1901657,GO:1901659	6.3.5.2	ko:K01951
765913.ThidrDRAFT_2517	1.2e-39	155.0	COG1262@1|root,COG1262@2|Bacteria, 1QT7U@1224|Proteobacteria, 1RWY0@1236|Gammaproteobacteria, 1X0ZN@135613|Chromatiales	135613|Chromatiales	**S - Function unknown**	CDS with the same product name	-	-	-	-
1121445.ATUZ01000013_	1.23e-24	104.0	COG1624@1|root,COG1624@2|Bacteria, 1PEQT@1224|Proteobacteria, 42P5A@68525|delta/epsilon subdivisions, 2WKPP@28221|Deltaproteobacteria, 2M84W@213115|Desulfovibrionales	28221|Deltaproteobacteria	**S - Function unknown**	Catalyzes the condensation of 2 ATP molecules into cyclic di-AMP (c-di-AMP), a second messenger used to regulate differing processes in different bacteria	dacA	-	2.7.7.85	ko:K18672
886379.AEWI01000144_g	6.8e-71	229.0	COG2148@1|root,COG2148@2|Bacteria, 4NER4@976|Bacteroidetes, 2FNC2@200643|Bacteroidia, 3XIYM@558415|Marinilabiliaceae	976|Bacteroidetes	**M - Cell wall/membrane/envelope biogenesis**	CoA-binding domain	-	-	-	ko:K13012
1411123.JQNH01000001_	4.48e-22	98.6	COG3842@1|root,COG3842@2|Bacteria, 1MU3I@1224|Proteobacteria, 2TQMJ@28211|Alphaproteobacteria	28211|Alphaproteobacteria	**P - Inorganic ion transport and metabolism**	Part of the ABC transporter complex PotABCD involved in spermidine putrescine import. Responsible for energy coupling to the transport system	potA	GO:0005575,GO:0005623,GO:0005886,GO:0006810,GO:0006811,GO:0006812,GO:0008150,GO:0015695,GO:0015696,GO:0015846,GO:0015847,GO:0016020,GO:0016021,GO:0031224,GO:0032991,GO:0043190,GO:0044425,GO:0044459,GO:0044464,GO:0051179,GO:0051234,GO:0071702,GO:0071705,GO:0071944,GO:0098533,GO:0098796,GO:0098797,GO:1902494,GO:1902495,GO:1904949,GO:1990351	-	ko:K11076
247490.KSU1_D1049	2.05e-144	436.0	COG1178@1|root,COG1178@2|Bacteria, 2J51F@203682|Planctomycetes	203682|Planctomycetes	**U - Intracellular trafficking, secretion, and vesicular transport**	Binding-protein-dependent transport system inner membrane component	-	-	-	ko:K02011
59374.Fisuc_2251	2.89e-222	622.0	COG2148@1|root,COG2148@2|Bacteria	2|Bacteria	**M - Cell wall/membrane/envelope biogenesis**	undecaprenyl-phosphate glucose phosphotransferase activity	cpsE	GO:0005575,GO:0005623,GO:0005886,GO:0016020,GO:0044464,GO:0071944	-	ko:K13012
59374.Fisuc_0672	7.05e-167	468.0	COG0101@1|root,COG0101@2|Bacteria	2|Bacteria	**J - Translation, ribosomal structure and biogenesis**	tRNA pseudouridine synthase activity	truA	GO:0000049,GO:0001522,GO:0003674,GO:0003676,GO:0003723,GO:0003824,GO:0005488,GO:0005575,GO:0005618,GO:0005623,GO:0006139,GO:0006396,GO:0006399,GO:0006400,GO:0006725,GO:0006807,GO:0008033,GO:0008150,GO:0008152,GO:0009451,GO:0009982,GO:0009987,GO:0010467,GO:0016070,GO:0016853,GO:0016866,GO:0030312,GO:0031119,GO:0034470,GO:0034641,GO:0034660,GO:0040007,GO:0043170,GO:0043412,GO:0044237,GO:0044238,GO:0044464,GO:0046483,GO:0071704,GO:0071944,GO:0090304,GO:0097159,GO:1901360,GO:1901363	5.4.99.12	ko:K06173
59374.Fisuc_2039	3.95e-53	181.0	COG2199@1|root,COG3706@2|Bacteria	2|Bacteria	**T - Signal transduction mechanisms**	GGDEF domain	-	-	-	-

	59374.Fisuc_2038	3.48e-211	610.0	COG1032@1|root,COG5011@1|root, COG1032@2|Bacteria, COG5011@2|Bacteria	2|Bacteria	**C - Energy production and conversion**	Protein conserved in bacteria	-	-	-	-
	59374.Fisuc_2253	1.24e-42	150.0	COG0460@1|root,COG0460@2|Bacteria	2|Bacteria	**E - Amino acid transport and metabolism**	homoserine dehydrogenase activity	hom	GO:0003674,GO:0003824,GO:0004412,GO:0005575,GO:0005618,GO:0005623,GO:0005886,GO:0006082,GO:0006520,GO:0006566,GO:0006807,GO:0008150,GO:0008152,GO:0008652,GO:0009058,GO:0009066,GO:0009067,GO:0009088,GO:0009987,GO:0016020,GO:0016053,GO:0016491,GO:0016614,GO:0016616,GO:0019752,GO:0030312,GO:0040007,GO:0043436,GO:0044237,GO:0044238,GO:0044249,GO:0044281,GO:0044283,GO:0044464,GO:0046394,GO:0055114,GO:0071704,GO:0071944,GO:1901564,GO:1901566,GO:1901576,GO:1901605,GO:1901607	1.1.1.3	ko:K00003
	59374.Fisuc_2254	9.4e-271	748.0	COG1086@1|root,COG2148@1|root, COG1086@2|Bacteria, COG2148@2|Bacteria	2|Bacteria	**M - Cell wall/membrane/envelope biogenesis**	undecaprenyl-phosphate glucose phosphotransferase activity	wcaJ	GO:0000271,GO:0003674,GO:0003824,GO:0005975,GO:0005976,GO:0006629,GO:0008150,GO:0008152,GO:0008610,GO:0008653,GO:0009058,GO:0009059,GO:0009103,GO:0009243,GO:0009987,GO:0016051,GO:0016740,GO:0016772,GO:0016780,GO:0033692,GO:0034637,GO:0034645,GO:0043170,GO:0044237,GO:0044238,GO:0044249,GO:0044255,GO:0044260,GO:0044262,GO:0044264,GO:0046402,GO:0071704,GO:1901135,GO:1901137,GO:1901576,GO:1903509	2.4.1.187,2.7.8.40	ko:K05946,ko:K21303
	59374.Fisuc_2039	2.45e-77	249.0	COG2199@1|root,COG3706@2|Bacteria	2|Bacteria	**T - Signal transduction mechanisms**	GGDEF domain	-	-	-	-
	59374.Fisuc_2038	4.32e-280	786.0	COG1032@1|root,COG5011@1|root, COG1032@2|Bacteria, COG5011@2|Bacteria	2|Bacteria	**C - Energy production and conversion**	Protein conserved in bacteria	-	-	-	-
	59374.Fisuc_2027	3.22e-142	407.0	COG1989@1|root,COG1989@2|Bacteria	2|Bacteria	**NOU : (N- Cell motility O- Post-translational modification, protein turnover, chaperone functions, and U - Intracellular trafficking, secretion,**	aspartic-type endopeptidase activity	pilD	-	3.4.23.43	ko:K02236,ko:K02278,ko:K02506,ko:K02654,ko:K10966
	935863. AWZR01000001_gene1758	1.62e-08	60.1	COG2091@1|root,COG2091@2|Bacteria, 1MZHC@1224|Proteobacteria, 1S466@1236|Gammaproteobacteria, 1X71D@135614|Xanthomonadales	135614|Xanthomonadales	**H - Coenzyme transport and metabolism**	Belongs to the P-Pant transferase superfamily	hetI	-	-	ko:K06133
	59374.Fisuc_2027	4.58e-17	79.3	COG1989@1|root,COG1989@2|Bacteria	2|Bacteria	**NOU**	aspartic-type endopeptidase activity	pilD	-	3.4.23.43	ko:K02236,ko:K02278,ko:K02506,ko:K02654,ko:K10966
	59374.Fisuc_2027	7.73e-146	417.0	COG1989@1|root,COG1989@2|Bacteria	2|Bacteria	**NOU**	aspartic-type endopeptidase activity	pilD	-	3.4.23.43	ko:K02236,ko:K02278,ko:K02506,ko:K02654,ko:K10966
	59374.Fisuc_2027	1.63e-106	315.0	COG1989@1|root,COG1989@2|Bacteria	2|Bacteria	**NOU**	aspartic-type endopeptidase activity	pilD	-	3.4.23.43	ko:K02236,ko:K02278,ko:K02506,ko:K02654,ko:K10966
	59374.Fisuc_2039	1.28e-119	358.0	COG2199@1|root,COG3706@2|Bacteria	2|Bacteria	**T - Signal transduction mechanisms**	GGDEF domain	-	-	-	-
	521003.COLINT_02374	1.43e-22	99.4	COG1032@1|root,COG1032@2|Bacteria, 2GJY1@201174|Actinobacteria, 4CUXU@84998|Coriobacteriia	84998|Coriobacteriia	**C - Energy production and conversion**	Radical SAM domain protein	-	-	-	-
	68170. KL590505_gene9150	3.14e-19	89.4	COG1032@1|root,COG1032@2|Bacteria, 2GJY1@201174|Actinobacteria, 4DXFP@85010|Pseudonocardiales	201174|Actinobacteria	**C - Energy production and conversion**	Elongator protein 3, MiaB family, Radical SAM	-	-	-	-
	59374.Fisuc_0672	2.32e-132	384.0	COG0101@1|root,COG0101@2|Bacteria	2|Bacteria	**J - Translation, ribosomal structure and biogenesis**	tRNA pseudouridine synthase activity	truA	GO:0000049,GO:0001522,GO:0003674,GO:0003676,GO:0003723,GO:0003824,GO:0005488,GO:0005575,GO:0005618,GO:0005623,GO:0006139,GO:0006396,GO:0006399,GO:0006400,GO:0006725,GO:0006807,GO:0008033,GO:0008150,GO:0008152,GO:0009451,GO:0009982,GO:0009987,GO:0010467,GO:0016070,GO:0016853,GO:0016866,GO:0030312,GO:0031119,GO:0034470,GO:0034641,GO:0034660,GO:0040007,GO:0043170,GO:0043412,GO:0044237,GO:0044238,GO:0044464,GO:0046483,GO:0071704,GO:0071944,GO:0090304,GO:0097159,GO:1901360,GO:1901363	5.4.99.12	ko:K06173
**HG4 containing contig**	59374.Fisuc_0672	4.39e-32	120.0	COG0101@1|root,COG0101@2|Bacteria	2|Bacteria	**J - Translation, ribosomal structure and biogenesis**	tRNA pseudouridine synthase activity	truA	GO:0000049,GO:0001522,GO:0003674,GO:0003676,GO:0003723,GO:0003824,GO:0005488,GO:0005575,GO:0005618,GO:0005623,GO:0006139,GO:0006396,GO:0006399,GO:0006400,GO:0006725,GO:0006807,GO:0008033,GO:0008150,GO:0008152,GO:0009451,GO:0009982,GO:0009987,GO:0010467,GO:0016070,GO:0016853,GO:0016866,GO:0030312,GO:0031119,GO:0034470,GO:0034641,GO:0034660,GO:0040007,GO:0043170,GO:0043412,GO:0044237,GO:0044238,GO:0044464,GO:0046483,GO:0071704,GO:0071944,GO:0090304,GO:0097159,GO:1901360,GO:1901363	5.4.99.12	ko:K06173

<table>
<tr><td>59374.Fisuc_2251</td><td>1.62e-233</td><td>648.0</td><td>COG2148@1|root,COG2148@2|Bacteria</td><td>2|Bacteria</td><td>M - Cell wall/membrane/envelope biogenesis</td><td>undecaprenyl-phosphate glucose phosphotransferase activity</td><td>cpsE</td><td>GO:0005575,GO:0005623,GO:0005886,GO:0016020,GO:0044464,GO:0071944</td><td>-</td><td>ko:K13012</td></tr>
<tr><td>59374.Fisuc_2253</td><td>9.63e-72</td><td>232.0</td><td>COG0460@1|root,COG0460@2|Bacteria</td><td>2|Bacteria</td><td>E - Amino acid transport and metabolism</td><td>homoserine dehydrogenase activity</td><td>hom</td><td>GO:0003674,GO:0003824,GO:0004412,GO:0005575,GO:0005618,GO:0005623,GO:0005886,GO:0006082,GO:0006520,GO:0006566,GO:0006807,GO:0008150,GO:0008152,GO:0008652,GO:0009058,GO:0009066,GO:0009067,GO:0009088,GO:0009987,GO:0016020,GO:0016053,GO:0016491,GO:0016614,GO:0016616,GO:0019752,GO:0030312,GO:0040007,GO:0043436,GO:0044237,GO:0044238,GO:0044249,GO:0044281,GO:0044283,GO:0044464,GO:0046394,GO:0055114,GO:0071704,GO:0071944,GO:1901564,GO:1901566,GO:1901576,GO:1901605,GO:1901607</td><td>1.1.1.3</td><td>ko:K00003</td></tr>
<tr><td>59374.Fisuc_2039</td><td>2.19e-67</td><td>221.0</td><td>COG2199@1|root,COG3706@2|Bacteria</td><td>2|Bacteria</td><td>T - Signal transduction mechanisms</td><td>GGDEF domain</td><td>-</td><td>-</td><td>-</td><td>-</td></tr>
<tr><td>59374.Fisuc_2251</td><td>2.86e-69</td><td>219.0</td><td>COG2148@1|root,COG2148@2|Bacteria</td><td>2|Bacteria</td><td>M - Cell wall/membrane/envelope biogenesis</td><td>undecaprenyl-phosphate glucose phosphotransferase activity</td><td>cpsE</td><td>GO:0005575,GO:0005623,GO:0005886,GO:0016020,GO:0044464,GO:0071944</td><td>-</td><td>ko:K13012</td></tr>
<tr><td>59374.Fisuc_2253</td><td>3.33e-164</td><td>470.0</td><td>COG0460@1|root,COG0460@2|Bacteria</td><td>2|Bacteria</td><td>E - Amino acid transport and metabolism</td><td>homoserine dehydrogenase activity</td><td>hom</td><td>GO:0003674,GO:0003824,GO:0004412,GO:0005575,GO:0005618,GO:0005623,GO:0005886,GO:0006082,GO:0006520,GO:0006566,GO:0006807,GO:0008150,GO:0008152,GO:0008652,GO:0009058,GO:0009066,GO:0009067,GO:0009088,GO:0009987,GO:0016020,GO:0016053,GO:0016491,GO:0016614,GO:0016616,GO:0019752,GO:0030312,GO:0040007,GO:0043436,GO:0044237,GO:0044238,GO:0044249,GO:0044281,GO:0044283,GO:0044464,GO:0046394,GO:0055114,GO:0071704,GO:0071944,GO:1901564,GO:1901566,GO:1901576,GO:1901605,GO:1901607</td><td>1.1.1.3</td><td>ko:K00003</td></tr>
<tr><td>59374.Fisuc_2253</td><td>6.94e-67</td><td>217.0</td><td>COG0460@1|root,COG0460@2|Bacteria</td><td>2|Bacteria</td><td>E - Amino acid transport and metabolism</td><td>homoserine dehydrogenase activity</td><td>hom</td><td>GO:0003674,GO:0003824,GO:0004412,GO:0005575,GO:0005618,GO:0005623,GO:0005886,GO:0006082,GO:0006520,GO:0006566,GO:0006807,GO:0008150,GO:0008152,GO:0008652,GO:0009058,GO:0009066,GO:0009067,GO:0009088,GO:0009987,GO:0016020,GO:0016053,GO:0016491,GO:0016614,GO:0016616,GO:0019752,GO:0030312,GO:0040007,GO:0043436,GO:0044237,GO:0044238,GO:0044249,GO:0044281,GO:0044283,GO:0044464,GO:0046394,GO:0055114,GO:0071704,GO:0071944,GO:1901564,GO:1901566,GO:1901576,GO:1901605,GO:1901607</td><td>1.1.1.3</td><td>ko:K00003</td></tr>
<tr><td>59374.Fisuc_2254</td><td>1.63e-82</td><td>258.0</td><td>COG1086@1|root,COG2148@1|root,COG1086@2|Bacteria,COG2148@2|Bacteria</td><td>2|Bacteria</td><td>M - Cell wall/membrane/envelope biogenesis</td><td>undecaprenyl-phosphate glucose phosphotransferase activity</td><td>wcaJ</td><td>GO:0000271,GO:0003674,GO:0003824,GO:0005975,GO:0005976,GO:0006629,GO:0008150,GO:0008152,GO:0008610,GO:0008653,GO:0009058,GO:0009059,GO:0009103,GO:0009243,GO:0009987,GO:0016051,GO:0016740,GO:0016772,GO:0016780,GO:0033692,GO:0034637,GO:0034645,GO:0043170,GO:0044237,GO:0044238,GO:0044249,GO:0044255,GO:0044260,GO:0044262,GO:0044264,GO:0046402,GO:0071704,GO:1901135,GO:1901137,GO:1901576,GO:1903509</td><td>2.4.1.187,2.7.8.40</td><td>ko:K05946,ko:K21303</td></tr>
<tr><td>59374.Fisuc_2254</td><td>3.27e-239</td><td>669.0</td><td>COG1086@1|root,COG2148@1|root,COG1086@2|Bacteria,COG2148@2|Bacteria</td><td>2|Bacteria</td><td>M - Cell wall/membrane/envelope biogenesis</td><td>undecaprenyl-phosphate glucose phosphotransferase activity</td><td>wcaJ</td><td>GO:0000271,GO:0003674,GO:0003824,GO:0005975,GO:0005976,GO:0006629,GO:0008150,GO:0008152,GO:0008610,GO:0008653,GO:0009058,GO:0009059,GO:0009103,GO:0009243,GO:0009987,GO:0016051,GO:0016740,GO:0016772,GO:0016780,GO:0033692,GO:0034637,GO:0034645,GO:0043170,GO:0044237,GO:0044238,GO:0044249,GO:0044255,GO:0044260,GO:0044262,GO:0044264,GO:0046402,GO:0071704,GO:1901135,GO:1901137,GO:1901576,GO:1903509</td><td>2.4.1.187,2.7.8.40</td><td>ko:K05946,ko:K21303</td></tr>
<tr><td>59374.Fisuc_2254</td><td>1.98e-105</td><td>318.0</td><td>COG1086@1|root,COG2148@1|root,COG1086@2|Bacteria,COG2148@2|Bacteria</td><td>2|Bacteria</td><td>M - Cell wall/membrane/envelope biogenesis</td><td>undecaprenyl-phosphate glucose phosphotransferase activity</td><td>wcaJ</td><td>GO:0000271,GO:0003674,GO:0003824,GO:0005975,GO:0005976,GO:0006629,GO:0008150,GO:0008152,GO:0008610,GO:0008653,GO:0009058,GO:0009059,GO:0009103,GO:0009243,GO:0009987,GO:0016051,GO:0016740,GO:0016772,GO:0016780,GO:0033692,GO:0034637,GO:0034645,GO:0043170,GO:0044237,GO:0044238,GO:0044249,GO:0044255,GO:0044260,GO:0044262,GO:0044264,GO:0046402,GO:0071704,GO:1901135,GO:1901137,GO:1901576,GO:1903509</td><td>2.4.1.187,2.7.8.40</td><td>ko:K05946,ko:K21303</td></tr>
<tr><td>1301100.HG529227_gene5476</td><td>4.24e-29</td><td>109.0</td><td>COG2148@1|root,COG2148@2|Bacteria,1TP7M@1239|Firmicutes,248WV@186801|Clostridia,36GR6@31979|Clostridiaceae</td><td>186801|Clostridia</td><td>M - Cell wall/membrane/envelope biogenesis</td><td>Exopolysaccharide biosynthesis polyprenyl glycosylphosphotransferase</td><td>-</td><td>-</td><td>-</td><td>-</td></tr>
</table>

1276756. AUEX01000004_gene2469	5.62e-24	97.8	COG2148@1|root,COG2148@2|Bacteria, 1MV6W@1224|Proteobacteria, 2VIY4@28216|Betaproteobacteria, 4ACXV@80864|Comamonadaceae	28216|Betaproteobacteria	**M - Cell wall/membrane/envelope biogenesis**	Bacterial sugar transferase	wcaJ	-	2.7.8.40	ko:K21303
59374.Fisuc_0427	4.21e-44	155.0	COG0697@1|root,COG0697@2|Bacteria	2|Bacteria	**EG**	spore germination	ydeD	-	-	-
59374.Fisuc_2914	2.43e-132	380.0	COG1521@1|root,COG1521@2|Bacteria	2|Bacteria	**F - Nucleotide transport and metabolism**	Catalyzes the phosphorylation of pantothenate (Pan), the first step in CoA biosynthesis	coaX	GO:0005575,GO:0005623,GO:0005886,GO:0016020,GO:0044464,GO:0071944	2.7.1.33	ko:K03525
59374.Fisuc_2256	3.39e-90	272.0	COG0451@1|root,COG0451@2|Bacteria	2|Bacteria	**GM**	ADP-glyceromanno-heptose 6-epimerase activity	uxs	-	4.1.1.35,4.2.1.46	ko:K01710,ko:K08678

Supplementary Table 5. Antimicrobial activity of Linear HG2 (without disulphide bond on cysteine residues).

Minimum inhibitory Concentrations MICs (µg/ml)				
**Bacteria strains**	**Antimicrobial compounds**			
	**HG2**	**HG4**	**Polymyxin B sulphate**	**Ciprofloxacin**
P. aeruginosa	>1024	≥1024	1.95	0.49
Sal. typhimurium	>1024	512	1.95	0.12
E. coli	>1024	512	1.95	0.06
EMRA-15	>1024	32	250	>250
S. aureus	>1024	32	250	>250
Ent. faecalis	>1024	128	31.25	62.5
List. monocytogenes	>1024	512	62.5	62.5

Supplementary Table 6. Cytotoxicity and haemolytic activities of HG2 and HG4 on human cells. Cytotoxicity is expressed as IC_{50} (i.e. the concentration of peptide in µg/ml causing a reduction of 50% of the cell viability). Haemolytic activity is expressed as HC_{50} (i.e. the concentration of peptide causing 50% haemolysis). IC_{50} and HC_{50} are expressed in µg/ml concentrations. Therapeutic Indexes (T.I) corresponding to the fold difference between IC_{50} or HC_{50} and MIC values (for MRSA USA300) are given in brackets.

**Cell type (human)**	**HG2 (**X MIC**)**	**HG4 (**X MIC**)**
**BEAS-2B**	120 +/- 25 (X 7.7)	>1000 (> 32)
**HEPG2**	359 +/- 76 (X 23)	>1000 (> 32)
**IMR-90**	96 +/- 21 (X 6.1)	294 +/- 42 (X 9.4)
**Erythrocytes**	409 +/- 67 (X 26.2)	458 +/- 101 (X 14.6)

Supplementary Table 7. Peptide lipid interaction and insertion measurements: interaction of peptides, HG2 and HG4 with total MRSA and erythrocyte lipid extracts, and interaction of HG2 and HG4 (at 1 μg/mL final concentration) with pure lipids the initial surface pressure of lipid monolayer. Maximal variation of surface pressure induced by the injection of peptide in lipid monolayer with initial surface pressure of 30+/-0.5 mN/m

**Lipids**	**HG2** Critical pressure of insertion π_c (mN/m)	**HG2** Speed of insertion (mN/ m/10 sec)	**HG4** Critical pressure of insertion π_c (mN/m)	**HG4** Speed of insertion (mN/m/10 sec)
Erythrocyte extract	35.07	1.13	30.99	1.20
MRSA extract	42.59	3.36	44.18	1.69
Cardiolipin	41.30	4.74	46.49	2.44
LTA	47.81	0.80	40.04	0.39
LPS	36.03	0.43	34.08	0.31
PC	62.79	8.58	28.8	0.50
PE	39.49	1.89	33.9	0.20
PG	51.19	3.70	47.55	2.48

Supplementary Table 8. Differentially Expressed Genes (DEGs) isolated from HG2 treated sample

S/N	Name	Differential Expression Log2 Ratio	protein_id	[redacted]	Gene ontology (biological process)	Gene ontology (cellular component)	Gene ontology (molecular function)	GO ID
1	farE CDS	-11.30840383	WP_001051429.1	[redacted]			integral component of membrane [GO:0016021]; plasma membrane [GO:0005886]	GO:0005886; GO:0016021
2	lrgA CDS	-10.01917583	WP_001792906.1	[redacted]	cytolysis [GO:0019835]; programmed cell death [GO:0012501]	cytolysis [GO:0019835]; programmed cell death [GO:0012501]	integral component of membrane [GO:0016021]; plasma membrane [GO:0005886]; cytolysis [GO:0019835]; programmed cell death [GO:0012501]	GO:0005886; GO:0012501; GO:0016021; GO:0019835
3	class I SAM-dependent methyltransferase CDS	-8.823072126	WP_000084829.1	[redacted]	methylation [GO:0032259]	methylation [GO:0032259]	methyltransferase activity [GO:0008168]; methylation [GO:0032259]	GO:0008168; GO:0032259
4	response regulator transcription factor CDS	-7.725336378	WP_000202389.1	[redacted]	phosphorelay signal transduction system [GO:0000160]; regulation of transcription, DNA-templated [GO:0006355]	phosphorelay signal transduction system [GO:0000160]; regulation of transcription, DNA-templated [GO:0006355]	DNA binding [GO:0003677]; phosphorelay signal transduction system [GO:0000160]; regulation of transcription, DNA-templated [GO:0006355]	GO:0000160; GO:0003677; GO:0006355
5	DUF1450 domain-containing protein CDS	-7.223249061	WP_000798968.1	[redacted]				
6	DNA-directed RNA polymerase subunit omega CDS	-7.1408456	WP_000933956.1	[redacted]	transcription, DNA-templated [GO:0006351]	transcription, DNA-templated [GO:0006351]	DNA binding [GO:0003677]; DNA-directed 5'-3' RNA polymerase activity [GO:0003899]; transcription, DNA-templated [GO:0006351]	GO:0003677; GO:0003899; GO:0006351
7	amino acid permease CDS	-6.265042967	WP_000389956.1	[redacted]	amino acid transport [GO:0006865]; transmembrane transport [GO:0055085]	amino acid transport [GO:0006865]; transmembrane transport [GO:0055085]	integral component of membrane [GO:0016021]; amino acid transport [GO:0006865]; transmembrane transport [GO:0055085]	GO:0006865; GO:0016021; GO:0055085
8	penicillin-binding protein CDS	-5.929129856	WP_000184370.1	[redacted]			integral component of membrane [GO:0016021]	GO:0016021
9	LCP family protein CDS	-5.836294579	WP_000356975.1	[redacted]			integral component of membrane [GO:0016021]; plasma membrane [GO:0005886]	GO:0005886; GO:0016021
10	dephospho-CoA kinase CDS	-5.002828038	WP_001127167.1	[redacted]	coenzyme A biosynthetic process [GO:0015937]	coenzyme A biosynthetic process [GO:0015937]	cytoplasm [GO:0005737]; ATP binding [GO:0005524]; dephospho-CoA kinase activity [GO:0004140]; coenzyme A biosynthetic process [GO:0015937]	GO:0004140; GO:0005524; GO:0005737; GO:0015937
11	NarK/NasA family nitrate transporter CDS	-4.925642769	WP_000278558.1	[redacted]	nitrate assimilation [GO:0042128]	nitrate assimilation [GO:0042128]	integral component of membrane [GO:0016021]; plasma membrane [GO:0005886]; transmembrane transporter activity [GO:0022857]; nitrate assimilation [GO:0042128]	GO:0005886; GO:0016021; GO:0022857; GO:0042128
12	lrgB CDS	-3.837821059	WP_000607067.1	[redacted]	cytolysis [GO:0019835]; programmed cell death [GO:0012501]	cytolysis [GO:0019835]; programmed cell death [GO:0012501]	integral component of membrane [GO:0016021]; plasma membrane [GO:0005886]; cytolysis [GO:0019835]; programmed cell death [GO:0012501]	GO:0005886; GO:0012501; GO:0016021; GO:0019835
13	amino acid permease CDS	-3.813483634	WP_000534425.1	[redacted]			integral component of membrane [GO:0016021]; transmembrane transporter activity [GO:0022857]	GO:0016021; GO:0022857
14	(S)-acetoin forming diacetyl reductase CDS	-3.795748682	WP_000183771.1	[redacted]	acetoin catabolic process [GO:0045150]	acetoin catabolic process [GO:0045150]	diacetyl reductase ((S)-acetoin forming) activity [GO:0052588]; acetoin catabolic process [GO:0045150]	GO:0045150; GO:0052588
15	DUF3387 domain-containing protein CDS	-3.780915861	WP_001549961.1	[redacted]				
16	nreC CDS	-3.760017955	WP_000706315.1	[redacted]	phosphorelay signal transduction system [GO:0000160]; regulation of transcription, DNA-templated [GO:0006355]	phosphorelay signal transduction system [GO:0000160]; regulation of transcription, DNA-templated [GO:0006355]	cytoplasm [GO:0005737]; DNA binding [GO:0003677]; phosphorelay signal transduction system [GO:0000160]; regulation of transcription, DNA-templated [GO:0006355]	GO:0000160; GO:0003677; GO:0005737; GO:0006355
17	nreB CDS	-3.70010162	WP_000606546.1	[redacted]			cytoplasm [GO:0005737]; integral component of membrane [GO:0016021]; 4 iron, 4 sulfur cluster binding [GO:0051539]; ATP binding [GO:0005524]; iron ion binding [GO:0005506]; phosphorelay sensor kinase activity [GO:0000155]; protein dimerization activity [GO:0046983]	GO:0000155; GO:0005506; GO:0005524; GO:0005737; GO:0016021; GO:0046983; GO:0051539
18	narI CDS	-3.595212842	WP_000934151.1	[redacted]	nitrate assimilation [GO:0042128]	nitrate assimilation [GO:0042128]	integral component of membrane [GO:0016021]; nitrate reductase complex [GO:0009325]; plasma membrane [GO:0005886]; metal ion binding [GO:0046872]; nitrate reductase activity [GO:0008940]; nitrate assimilation [GO:0042128]	GO:0005886; GO:0008940; GO:0009325; GO:0016021; GO:0042128; GO:0046872
19	sdaAB CDS	-3.546549727	WP_001140234.1	[redacted]	gluconeogenesis [GO:0006094]	gluconeogenesis [GO:0006094]	4 iron, 4 sulfur cluster binding [GO:0051539]; L-serine ammonia-lyase activity [GO:0003941]; metal ion binding [GO:0046872]; gluconeogenesis [GO:0006094]	GO:0003941; GO:0006094; GO:0046872; GO:0051539

Supplementary Table 8. Differentially Expressed Genes (DEGs) isolated from HG2 treated sample

20	nirD CDS	-3.502786463	WP_000448222.1	[redacted]	nitrate assimilation [GO:0042128]	nitrate assimilation [GO:0042128]	2 iron, 2 sulfur cluster binding [GO:0051537]; metal ion binding [GO:0046872]; nitrite reductase [NAD(P)H] activity [GO:0008942]; nitrate assimilation [GO:0042128]	GO:0008942; GO:0042128; GO:0046872; GO:0051537
21	amino acid permease CDS	-3.48836976	WP_000818525.1	[redacted]	transmembrane transport [GO:0055085]	transmembrane transport [GO:0055085]	integral component of membrane [GO:0016021]; transmembrane transport [GO:0055085]	GO:0016021; GO:0055085
22	msaA CDS	-3.457895695	WP_000269923.1	[redacted]				
23	narJ CDS	-3.450293315	WP_000606768.1	[redacted]	chaperone-mediated protein complex assembly [GO:0051131]; nitrate assimilation [GO:0042128]	chaperone-mediated protein complex assembly [GO:0051131]; nitrate assimilation [GO:0042128]	unfolded protein binding [GO:0051082]; chaperone-mediated protein complex assembly [GO:0051131]; nitrate assimilation [GO:0042128]	GO:0042128; GO:0051082; GO:0051131
24	heavy-metal-associated domain-containing protein CDS	-3.429524643	WP_000581792.1	[redacted]			metal ion binding [GO:0046872]	GO:0046872
25	amino acid permease CDS	-3.306044368	WP_000101538.1	[redacted]	transmembrane transport [GO:0055085]	transmembrane transport [GO:0055085]	integral component of membrane [GO:0016021]; transmembrane transport [GO:0055085]	GO:0016021; GO:0055085
26	urease subunit gamma CDS	-3.287095778	WP_000545928.1	[redacted]	urea catabolic process [GO:0043419]	urea catabolic process [GO:0043419]	cytoplasm [GO:0005737]; nickel cation binding [GO:0016151]; urease activity [GO:0009039]; urea catabolic process [GO:0043419]	GO:0005737; GO:0009039; GO:0016151; GO:0043419
27	Veg family protein CDS	-3.258565466	WP_001126664.1	[redacted]	regulation of transcription, DNA-templated [GO:0006355]	regulation of transcription, DNA-templated [GO:0006355]	regulation of transcription, DNA-templated [GO:0006355]	GO:0006355
28	nreA CDS	-3.153049033	WP_000186933.1	[redacted]				
29	narH CDS	-3.114843431	WP_000692645.1	[redacted]	nitrate metabolic process [GO:0042126]	nitrate metabolic process [GO:0042126]	nitrate reductase complex [GO:0009325]; nitrate reductase activity [GO:0008940]; nitrate metabolic process [GO:0042126]	GO:0008940; GO:0009325; GO:0042126
30	formate/nitrite transporter family protein CDS	-3.072371321	WP_000572040.1	[redacted]			integral component of membrane [GO:0016021]; transmembrane transporter activity [GO:0022857]	GO:0016021; GO:0022857
31	cold-shock protein CDS	-2.983735897	WP_001059082.1	[redacted]			cytoplasm [GO:0005737]; nucleic acid binding [GO:0003676]	GO:0003676; GO:0005737
32	Nramp family divalent metal transporter CDS	-2.948086848	WP_001060842.1	[redacted]			integral component of membrane [GO:0016021]; plasma membrane [GO:0005886]; metal ion binding [GO:0046872]; metal ion transmembrane transporter activity [GO:0046873]; symporter activity [GO:0015293]	GO:0005886; GO:0015293; GO:0016021; GO:0046872; GO:0046873
33	cold-shock protein CDS	-2.927664076	WP_001059079.1	[redacted]			cytoplasm [GO:0005737]; DNA binding [GO:0003677]	GO:0003677; GO:0005737
34	putP CDS	-2.903750884	WP_000957015.1	[redacted]	proline transport [GO:0015824]	proline transport [GO:0015824]	integral component of membrane [GO:0016021]; plasma membrane [GO:0005886]; proline:sodium symporter activity [GO:0005298]; sodium ion binding [GO:0031402]; proline transport [GO:0015824]	GO:0005298; GO:0005886; GO:0015824; GO:0016021; GO:0031402
35	L-lactate permease CDS	-2.835720952	WP_000960852.1	[redacted]			integral component of plasma membrane [GO:0005887]; lactate transmembrane transporter activity [GO:0015129]	GO:0005887; GO:0015129
36	srtA CDS	-2.816104526	WP_000759361.1	[redacted]			integral component of membrane [GO:0016021]; plasma membrane [GO:0005886]; cysteine-type peptidase activity [GO:0008234]; metal ion binding [GO:0046872]	GO:0005886; GO:0008234; GO:0016021; GO:0046872
37	cytochrome ubiquinol oxidase subunit I CDS	-2.792526687	WP_000381849.1	[redacted]	aerobic electron transport chain [GO:0019646]	aerobic electron transport chain [GO:0019646]	cytochrome complex [GO:0070069]; integral component of membrane [GO:0016021]; plasma membrane [GO:0005886]; electron transfer activity [GO:0009055]; metal ion binding [GO:0046872]; aerobic electron transport chain [GO:0019646]	GO:0005886; GO:0009055; GO:0016021; GO:0019646; GO:0046872; GO:0070069
38	kinase-associated lipoprotein B CDS	-2.762945907	WP_000781156.1	[redacted]			kinase activity [GO:0016301]	GO:0016301
39	transposase CDS	-2.75747142	WP_000159787.1	[redacted]	transposition, DNA-mediated [GO:0006313]	transposition, DNA-mediated [GO:0006313]	DNA binding [GO:0003677]; transposase activity [GO:0004803]; transposition, DNA-mediated [GO:0006313]	GO:0003677; GO:0004803; GO:0006313
40	IS1182 family transposase CDS	-2.706396874		[redacted]				

Supplementary Table 8. Differentially Expressed Genes (DEGs) isolated from HG2 treated sample

41	PTS fructose transporter subunit IIC CDS	-2.700733556	WP_000353634.1	[redacted]	phosphoenolpyruvate-dependent sugar phosphotransferase system [GO:0009401]	phosphoenolpyruvate-dependent sugar phosphotransferase system [GO:0009401]	integral component of membrane [GO:0016021]; plasma membrane [GO:0005886]; protein-N(PI)-phosphohistidine-sugar phosphotransferase activity [GO:0008982]; phosphoenolpyruvate-dependent sugar phosphotransferase system [GO:0009401]	GO:0005886; GO:0008982; GO:0009401; GO:0016021
42	holA CDS	-2.670769457	WP_001282562.1	[redacted]	DNA replication [GO:0006260]	DNA replication [GO:0006260]	DNA polymerase III complex [GO:0009360]; DNA binding [GO:0003677]; DNA-directed DNA polymerase activity [GO:0003887]; DNA replication [GO:0006260]	GO:0003677; GO:0003887; GO:0006260; GO:0009360
43	csoR CDS	-2.66861595	WP_000138932.1	[redacted]	regulation of transcription, DNA-templated [GO:0006355]	regulation of transcription, DNA-templated [GO:0006355]	DNA binding [GO:0003677]; metal ion binding [GO:0046872]; regulation of transcription, DNA-templated [GO:0006355]	GO:0003677; GO:0006355; GO:0046872
44	DASS family sodium-coupled anion symporter CDS	-2.644967961	WP_001288049.1	[redacted]			integral component of membrane [GO:0016021]; transmembrane transporter activity [GO:0022857]	GO:0016021; GO:0022857
45	DUF2871 domain-containing protein CDS	-2.641271777	WP_001253464.1	[redacted]			integral component of membrane [GO:0016021]	GO:0016021
46	mreC CDS	-2.640164443	WP_000911041.1	[redacted]	regulation of cell shape [GO:0008360]	regulation of cell shape [GO:0008360]	regulation of cell shape [GO:0008360]	GO:0008360
47	ABC transporter substrate-binding protein CDS	-2.594557887	WP_000517187.1	[redacted]	transmembrane transport [GO:0055085]	transmembrane transport [GO:0055085]	ATP-binding cassette (ABC) transporter complex [GO:0043190]; transmembrane transport [GO:0055085]	GO:0043190; GO:0055085
48	cobA CDS	-2.548744873	WP_000109968.1	[redacted]	methylation [GO:0032259]; siroheme biosynthetic process [GO:0019354]	methylation [GO:0032259]; siroheme biosynthetic process [GO:0019354]	uroporphyrin-III C-methyltransferase activity [GO:0004851]; methylation [GO:0032259]; siroheme biosynthetic process [GO:0019354]	GO:0004851; GO:0019354; GO:0032259
49	biotin-dependent carboxyltransferase family protein CDS	-2.543868197	WP_000022323.1	[redacted]			ATP binding [GO:0005524]; hydrolase activity [GO:0016787]	GO:0005524; GO:0016787
50	nitrate reductase subunit alpha CDS	-2.528694922	WP_000514391.1	[redacted]	nitrate metabolic process [GO:0042126]	nitrate metabolic process [GO:0042126]	nitrate reductase complex [GO:0009325]; plasma membrane [GO:0005886]; 4 iron, 4 sulfur cluster binding [GO:0051539]; metal ion binding [GO:0046872]; molybdopterin cofactor binding [GO:0043546]; nitrate reductase activity [GO:0008940]; nitrate metabolic process [GO:0042126]	GO:0005886; GO:0008940; GO:0009325; GO:0042126; GO:0043546; GO:0046872; GO:0051539
51	traP CDS	-2.522312924	WP_000737976.1	[redacted]	pathogenesis [GO:0009405]	pathogenesis [GO:0009405]	membrane [GO:0016020]; pathogenesis [GO:0009405]	GO:0009405; GO:0016020
52	NAD(P)/FAD-dependent oxidoreductase CDS	-2.483583096	WP_001118736.1	[redacted]	nitrate assimilation [GO:0042128]	nitrate assimilation [GO:0042128]	4 iron, 4 sulfur cluster binding [GO:0051539]; flavin adenine dinucleotide binding [GO:0050660]; heme binding [GO:0020037]; metal ion binding [GO:0046872]; NADP binding [GO:0050661]; nitrite reductase [NAD(P)H] activity [GO:0008942]; nitrate assimilation [GO:0042128]	GO:0008942; GO:0020037; GO:0042128; GO:0046872; GO:0050660; GO:0050661; GO:0051539
53	exonuclease SbcCD subunit D CDS	-2.462862144	WP_000238227.1	[redacted]			hydrolase activity [GO:0016787]	GO:0016787
54	upp CDS	-2.448346285	WP_000048712.1	[redacted]	nucleoside metabolic process [GO:0009116]; UMP salvage [GO:0044206]; uracil salvage [GO:0006223]	nucleoside metabolic process [GO:0009116]; UMP salvage [GO:0044206]; uracil salvage [GO:0006223]	GTP binding [GO:0005525]; magnesium ion binding [GO:0000287]; uracil phosphoribosyltransferase activity [GO:0004845]; nucleoside metabolic process [GO:0009116]; UMP salvage [GO:0044206]; uracil salvage [GO:0006223]	GO:0000287; GO:0004845; GO:0005525; GO:0006223; GO:0009116; GO:0044206
55	peptide MFS transporter CDS	-2.398035159	WP_000193752.1	[redacted]	oligopeptide transport [GO:0006857]	oligopeptide transport [GO:0006857]	integral component of membrane [GO:0016021]; plasma membrane [GO:0005886]; peptide transmembrane transporter activity [GO:1904680]; oligopeptide transport [GO:0006857]	GO:0005886; GO:0006857; GO:0016021; GO:1904680
56	response regulator transcription factor CDS	-2.380658046		[redacted]				
57	LacI family DNA-binding transcriptional regulator CDS	-2.37833836	WP_000256338.1	[redacted]	regulation of transcription, DNA-templated [GO:0006355]	regulation of transcription, DNA-templated [GO:0006355]	DNA binding [GO:0003677]; regulation of transcription, DNA-templated [GO:0006355]	GO:0003677; GO:0006355
58	membrane protein CDS	-2.356458567	WP_001024035.1	[redacted]			integral component of membrane [GO:0016021]	GO:0016021
59	hypothetical protein CDS	-2.352040652	WP_001788599.1	[redacted]				
60	ytxJ CDS	-2.323996491	WP_000985617.1	[redacted]				

Supplementary Table 8. Differentially Expressed Genes (DEGs) isolated from HG2 treated sample

61	quinone-dependent dihydroorotate dehydrogenase CDS	-2.314752783	WP_000277415.1	[redacted]	'de novo' pyrimidine nucleobase biosynthetic process [GO:0006207]; 'de novo' UMP biosynthetic process [GO:0044205]	'de novo' pyrimidine nucleobase biosynthetic process [GO:0006207]; 'de novo' UMP biosynthetic process [GO:0044205]	cytoplasm [GO:0005737]; plasma membrane [GO:0005886]; dihydroorotate dehydrogenase activity [GO:0004152]; orotate reductase (NADH) activity [GO:0004589]; 'de novo' pyrimidine nucleobase biosynthetic process [GO:0006207]; 'de novo' UMP biosynthetic process [GO:0044205]	GO:0004152; GO:0004589; GO:0005737; GO:0005886; GO:0006207; GO:0044205
62	metal-dependent transcriptional regulator CDS	-2.288350675	WP_000954654.1	[redacted]			DNA binding [GO:0003677]; DNA-binding transcription factor activity [GO:0003700]; protein dimerization activity [GO:0046983]; transition metal ion binding [GO:0046914]	GO:0003677; GO:0003700; GO:0046914; GO:0046983
63	ABC transporter permease CDS	-2.286282403	WP_000593417.1	[redacted]	nickel cation transport [GO:0015675]; transmembrane transport [GO:0055085]	nickel cation transport [GO:0015675]; transmembrane transport [GO:0055085]	integral component of membrane [GO:0016021]; plasma membrane [GO:0005886]; nickel cation transport [GO:0015675]; transmembrane transport [GO:0055085]	GO:0005886; GO:0015675; GO:0016021; GO:0055085
64	hypothetical protein CDS	-2.276216245	WP_000828354.1	[redacted]				
65	APC family permease CDS	-2.274749157	WP_000483049.1	[redacted]			integral component of membrane [GO:0016021]; transmembrane transporter activity [GO:0022857]	GO:0016021; GO:0022857
66	hypothetical protein CDS	-2.271644549	WP_001790562.1	[redacted]				
67	cytochrome d ubiquinol oxidase subunit II CDS	-2.249950309	WP_000638456.1	[redacted]			integral component of membrane [GO:0016021]; plasma membrane [GO:0005886]; oxidoreductase activity [GO:0016491]	GO:0005886; GO:0016021; GO:0016491
68	hypothetical protein CDS	-2.248749647	WP_001251935.1	[redacted]				
69	yfhO CDS	-2.248677232	WP_000915229.1	[redacted]			integral component of membrane [GO:0016021]; transferase activity [GO:0016740]	GO:0016021; GO:0016740
70	4-oxalocrotonate tautomerase CDS	-2.246540446	WP_001123276.1	[redacted]	cellular aromatic compound metabolic process [GO:0006725]	cellular aromatic compound metabolic process [GO:0006725]	isomerase activity [GO:0016853]; cellular aromatic compound metabolic process [GO:0006725]	GO:0006725; GO:0016853
71	YwpF-like family protein CDS	-2.242105648	WP_000846746.1	[redacted]				
72	PTS-dependent dihydroxyacetone kinase phosphotransferase subunit DhaM CDS	-2.238042017	WP_001124167.1	[redacted]	glycerol catabolic process [GO:0019563]; phosphoenolpyruvate-dependent sugar phosphotransferase system [GO:0009401]	glycerol catabolic process [GO:0019563]; phosphoenolpyruvate-dependent sugar phosphotransferase system [GO:0009401]	integral component of membrane [GO:0016021]; phosphoenolpyruvate-glycerone phosphotransferase activity [GO:0047324]; glycerol catabolic process [GO:0019563]; phosphoenolpyruvate-dependent sugar phosphotransferase system [GO:0009401]	GO:0009401; GO:0016021; GO:0019563; GO:0047324
73	DUF420 domain-containing protein CDS	-2.227081854	WP_000538766.1	[redacted]			integral component of membrane [GO:0016021]	GO:0016021
74	S1 RNA-binding domain-containing protein CDS	-2.218519839	WP_001067294.1	[redacted]			nucleic acid binding [GO:0003676]	GO:0003676
75	YjzD family protein CDS	-2.186535552	WP_000682089.1	[redacted]			integral component of membrane [GO:0016021]	GO:0016021
76	hypothetical protein CDS	-2.17857337	WP_001790128.1	[redacted]				
77	heme A synthase CDS	-2.163798773	WP_000467123.1	[redacted]	heme A biosynthetic process [GO:0006784]	heme A biosynthetic process [GO:0006784]	integral component of membrane [GO:0016021]; plasma membrane [GO:0005886]; oxidoreductase activity, acting on the CH-CH group of donors [GO:0016627]; heme A biosynthetic process [GO:0006784]	GO:0005886; GO:0006784; GO:0016021; GO:0016627
78	dltD CDS	-2.117738355	WP_000769417.1	[redacted]	lipoteichoic acid biosynthetic process [GO:0070395]	lipoteichoic acid biosynthetic process [GO:0070395]	integral component of membrane [GO:0016021]; plasma membrane [GO:0005886]; lipoteichoic acid biosynthetic process [GO:0070395]	GO:0005886; GO:0016021; GO:0070395
79	protoheme IX farnesyltransferase CDS	-2.116667352	WP_000031401.1	[redacted]	heme O biosynthetic process [GO:0048034]	heme O biosynthetic process [GO:0048034]	integral component of membrane [GO:0016021]; plasma membrane [GO:0005886]; protoheme IX farnesyltransferase activity [GO:0008495]; heme O biosynthetic process [GO:0048034]	GO:0005886; GO:0008495; GO:0016021; GO:0048034
80	GGDEF domain-containing protein CDS	-2.110446368	WP_000460983.1	[redacted]	cell wall organization [GO:0071555]	cell wall organization [GO:0071555]	integral component of membrane [GO:0016021]; plasma membrane [GO:0005886]; phosphorelay sensor kinase activity [GO:0000155]; cell wall organization [GO:0071555]	GO:0000155; GO:0005886; GO:0016021; GO:0071555
81	hypothetical protein CDS	-2.094059913	WP_000110603.1	[redacted]				
82	hypothetical protein CDS	-2.090938764	WP_000267034.1	[redacted]			integral component of membrane [GO:0016021]	GO:0016021
83	hypothetical protein CDS	-2.06964456	WP_001789909.1	[redacted]				

Supplementary Table 8. Differentially Expressed Genes (DEGs) isolated from HG2 treated sample

84	BCCT family transporter CDS	-2.040946873	WP_000792345.1	[redacted]	nitrogen compound transport [GO:0071705]	nitrogen compound transport [GO:0071705]	integral component of membrane [GO:0016021]; plasma membrane [GO:0005886]; transmembrane transporter activity [GO:0022857]; nitrogen compound transport [GO:0071705]	GO:0005886; GO:0016021; GO:0022857; GO:0071705
85	spxA CDS	-2.034624093	WP_000258003.1	[redacted]	negative regulation of transcription, DNA-templated [GO:0045892]	negative regulation of transcription, DNA-templated [GO:0045892]	cytoplasm [GO:0005737]; negative regulation of transcription, DNA-templated [GO:0045892]	GO:0005737; GO:0045892
86	50S ribosomal protein L28 CDS	-2.032699189	WP_000517908.1	[redacted]	translation [GO:0006412]	translation [GO:0006412]	ribosome [GO:0005840]; structural constituent of ribosome [GO:0003735]; translation [GO:0006412]	GO:0003735; GO:0005840; GO:0006412
87	qoxA CDS	-2.025483708	WP_000032836.1	[redacted]			integral component of membrane [GO:0016021]; plasma membrane [GO:0005886]; respirasome [GO:0070469]; copper ion binding [GO:0005507]; cytochrome bo3 ubiquinol oxidase activity [GO:0009486]; cytochrome-c oxidase activity [GO:0004129]; oxidoreductase activity, acting on diphenols and related substances as donors, oxygen as acceptor [GO:0016682]	GO:0004129; GO:0005507; GO:0005886; GO:0009486; GO:0016021; GO:0016682; GO:0070469
88	MFS transporter CDS	-2.022734292	WP_000381270.1	[redacted]			integral component of membrane [GO:0016021]; plasma membrane [GO:0005886]; transmembrane transporter activity [GO:0022857]	GO:0005886; GO:0016021; GO:0022857
89	sterile alpha motif-like domain-containing protein CDS	-2.007836019	WP_000011687.1	[redacted]				
90	DUF368 domain-containing protein CDS	-2.002752489	WP_001183858.1	[redacted]			integral component of membrane [GO:0016021]	GO:0016021
91	organic hydroperoxide resistance protein CDS	-2.001810129	WP_000974460.1	[redacted]	response to oxidative stress [GO:0006979]	response to oxidative stress [GO:0006979]	response to oxidative stress [GO:0006979]	GO:0006979
92	pfkA CDS	2.002458277	WP_000717561.1	[redacted]	fructose 6-phosphate metabolic process [GO:0006002]	fructose 6-phosphate metabolic process [GO:0006002]	cytoplasm [GO:0005737]; 6-phosphofructokinase activity [GO:0003872]; ATP binding [GO:0005524]; metal ion binding [GO:0046872]; fructose 6-phosphate metabolic process [GO:0006002]	GO:0003872; GO:0005524; GO:0005737; GO:0006002; GO:0046872
93	sufD CDS	2.054635221	WP_000205572.1	[redacted]	iron-sulfur cluster assembly [GO:0016226]	iron-sulfur cluster assembly [GO:0016226]	iron-sulfur cluster assembly [GO:0016226]	GO:0016226
94	rpmD CDS	2.059963118	WP_001096577.1	[redacted]	translation [GO:0006412]	translation [GO:0006412]	large ribosomal subunit [GO:0015934]; structural constituent of ribosome [GO:0003735]; translation [GO:0006412]	GO:0003735; GO:0006412; GO:0015934
95	ABC transporter permease CDS	2.113214525	WP_000876318.1	[redacted]			integral component of membrane [GO:0016021]; plasma membrane [GO:0005886]; transmembrane transporter activity [GO:0022857]	GO:0005886; GO:0016021; GO:0022857
96	guaC CDS	2.132165781	WP_000688126.1	[redacted]	purine nucleotide metabolic process [GO:0006163]	purine nucleotide metabolic process [GO:0006163]	GMP reductase complex [GO:1902560]; GMP reductase activity [GO:0003920]; purine nucleotide metabolic process [GO:0006163]	GO:0003920; GO:0006163; GO:1902560
97	argininosuccinate synthase CDS	2.144738598	WP_000660045.1	[redacted]	arginine biosynthetic process [GO:0006526]	arginine biosynthetic process [GO:0006526]	cytoplasm [GO:0005737]; argininosuccinate synthase activity [GO:0004055]; ATP binding [GO:0005524]; arginine biosynthetic process [GO:0006526]	GO:0004055; GO:0005524; GO:0005737; GO:0006526
98	PLP-dependent aminotransferase family protein CDS	2.171846235	WP_000799248.1	[redacted]	biosynthetic process [GO:0009058]	biosynthetic process [GO:0009058]	DNA-binding transcription factor activity [GO:0003700]; pyridoxal phosphate binding [GO:0030170]; transaminase activity [GO:0008483]; biosynthetic process [GO:0009058]	GO:0003700; GO:0008483; GO:0009058; GO:0030170
99	pdxS CDS	2.17445988	WP_000034728.1	[redacted]	pyridoxal phosphate biosynthetic process [GO:0042823]	pyridoxal phosphate biosynthetic process [GO:0042823]	pyridoxal 5'-phosphate synthase (glutamine hydrolysing) activity [GO:0036381]; pyridoxal phosphate biosynthetic process [GO:0042823]	GO:0036381; GO:0042823
100	CDP-glycerol glycerophosphotransferase family protein CDS	2.269646627	WP_000595298.1	[redacted]	teichoic acid biosynthetic process [GO:0019350]	teichoic acid biosynthetic process [GO:0019350]	plasma membrane [GO:0005886]; CDP-glycerol glycerophosphotransferase activity [GO:0047355]; teichoic acid biosynthetic process [GO:0019350]	GO:0005886; GO:0019350; GO:0047355
101	vraR CDS	2.351645181	WP_000153530.1	[redacted]	phosphorelay signal transduction system [GO:0000160]; regulation of transcription, DNA-templated [GO:0006355]	phosphorelay signal transduction system [GO:0000160]; regulation of transcription, DNA-templated [GO:0006355]	DNA binding [GO:0003677]; phosphorelay signal transduction system [GO:0000160]; regulation of transcription, DNA-templated [GO:0006355]	GO:0000160; GO:0003677; GO:0006355
102	vraD CDS	2.459955391	WP_000154162.1	[redacted]			ATP binding [GO:0005524]	GO:0005524
103	ABC transporter substrate-binding protein CDS	2.565682289	WP_001214661.1	[redacted]				

Supplementary Table 8. Differentially Expressed Genes (DEGs) isolated from HG2 treated sample

104	helix-turn-helix transcriptional regulator CDS	2.59028116	WP_000451438.1	[redacted]				
105	gluconate permease CDS	2.632164208	WP_000481425.1	[redacted]			integral component of membrane [GO:0016021]; gluconate transmembrane transporter activity [GO:0015128]	GO:0015128; GO:0016021
106	NADH-dependent flavin oxidoreductase CDS	2.634772098	WP_000838037.1	[redacted]			FMN binding [GO:0010181]; oxidoreductase activity [GO:0016491]	GO:0010181; GO:0016491
107	M42 family metallopeptidase CDS	2.636591049	WP_001076671.1	[redacted]			aminopeptidase activity [GO:0004177]; cellulase activity [GO:0008810]; metal ion binding [GO:0046872]	GO:0004177; GO:0008810; GO:0046872
108	TerC family protein CDS	2.806246403	WP_000928413.1	[redacted]			integral component of membrane [GO:0016021]; plasma membrane [GO:0005886]	GO:0005886; GO:0016021
109	sensor histidine kinase CDS	2.858134202	WP_001017131.1	[redacted]			integral component of membrane [GO:0016021]; plasma membrane [GO:0005886]; ATP binding [GO:0005524]; phosphorelay sensor kinase activity [GO:0000155]; protein dimerization activity [GO:0046983]	GO:0000155; GO:0005524; GO:0005886; GO:0016021; GO:0046983
110	YmfK family protein CDS	2.878188392	WP_000214890.1	[redacted]				
111	gntK CDS	2.886039075	WP_000876663.1	[redacted]	D-gluconate metabolic process [GO:0019521]	D-gluconate metabolic process [GO:0019521]	ATP binding [GO:0005524]; gluconokinase activity [GO:0046316]; glycerol kinase activity [GO:0004370]; D-gluconate metabolic process [GO:0019521]	GO:0004370; GO:0005524; GO:0019521; GO:0046316
112	GntR family transcriptional regulator CDS	3.29413264	WP_000990133.1	[redacted]			DNA binding [GO:0003677]; DNA-binding transcription factor activity [GO:0003700]; transaminase activity [GO:0008483]	GO:0003677; GO:0003700; GO:0008483
113	glmS CDS	3.375138041	WP_000334465.1	[redacted]	carbohydrate derivative biosynthetic process [GO:1901137]; carbohydrate metabolic process [GO:0005975]; glutamine metabolic process [GO:0006541]	carbohydrate derivative biosynthetic process [GO:1901137]; carbohydrate metabolic process [GO:0005975]; glutamine metabolic process [GO:0006541]	cytoplasm [GO:0005737]; carbohydrate derivative binding [GO:0097367]; glutamine-fructose-6-phosphate transaminase (isomerizing) activity [GO:0004360]; carbohydrate derivative biosynthetic process [GO:1901137]; carbohydrate metabolic process [GO:0005975]; glutamine metabolic process [GO:0006541]	GO:0004360; GO:0005737; GO:0005975; GO:0006541; GO:0097367; GO:1901137
114	fmtA CDS	8.714976044	WP_000671243.1	[redacted]	cell wall organization [GO:0071555]; response to antibiotic [GO:0046677]	cell wall organization [GO:0071555]; response to antibiotic [GO:0046677]	plasma membrane [GO:0005886]; hydrolase activity [GO:0016787]; cell wall organization [GO:0071555]; response to antibiotic [GO:0046677]	GO:0005886; GO:0016787; GO:0046677; GO:0071555

Supplementary Table 9. Frequencies of Top 10 statistically significant Gene Ontology (GO) ID and description in the Differentially expressed genes (DEGs) in the HG2 treatment group

GO ID	Count	Percentage Occurrence in DEGs	Term Descriptions
**GO:0016021**	42	13.5%	integral component of membrane
**GO:0005886**	28	9.0%	plasma membrane
**GO:0046872**	12	3.9%	metal ion binding
**GO:0003677**	11	3.5%	DNA binding
**GO:0005737**	11	3.5%	cytoplasm
**GO:0005524**	8	2.6%	ATP binding
**GO:0022857**	8	2.6%	transmembrane transporter activity
**GO:0006355**	6	1.9%	regulation of transcription, DNA-templated
**GO:0042128**	5	1.6%	nitrate assimilation
**GO:0055085**	5	1.6%	transmembrane transport

Supplementary Table 10. Differentially Expressed Genes (DEGs) isolated from HG4 treated sample

S/N	Name	Differential Expression Log2 Ratio	Differential Expression p-value	protein_id	Gene ontology (biological process)	Gene ontology (cellular component)	Gene ontology (molecular function)	GO ID
1	methionine adenosyltransferase CDS	-8.938612437	6.79E-52	WP_000933822.1	one-carbon metabolic process [GO:0006730]; S-adenosylmethionine biosynthetic process [GO:0006556]	one-carbon metabolic process [GO:0006730]; S-adenosylmethionine biosynthetic process [GO:0006556]	cytoplasm [GO:0005737]; ATP binding [GO:0005524]; magnesium ion binding [GO:0000287]; methionine adenosyltransferase activity [GO:0004478]; one-carbon metabolic process [GO:0006730]; S-adenosylmethionine biosynthetic process [GO:0006556]	GO:0000287; GO:0004478; GO:0005524; GO:0005737; GO:0006556; GO:0006730
2	hypothetical protein CDS	-4.061000848	1.49E-05	WP_000184341.1				
3	hypothetical protein CDS	-3.959693763	4.82E-05	WP_000695365.1				
4	hypothetical protein CDS	-3.624370342	6.67E-06	WP_001790530.1				
5	amino acid ABC transporter ATP-binding protein CDS	-3.574693876	1.69E-11	WP_000590809.1			ABC-type amino acid transporter activity [GO:0015424]; ATP binding [GO:0005524]; ATPase-coupled transmembrane transporter activity [GO:0042626]	GO:0005524; GO:0015424; GO:0042626
6	ABC transporter permease subunit CDS	-3.499145706	2.27E-11	WP_000649907.1	amino acid transport [GO:0006865]; nickel cation transport [GO:0015675]	amino acid transport [GO:0006865]; nickel cation transport [GO:0015675]	ATP-binding cassette (ABC) transporter complex [GO:0043190]; transmembrane transporter activity [GO:0022857]; amino acid transport [GO:0006865]; nickel cation transport [GO:0015675]	GO:0006865; GO:0015675; GO:0022857; GO:0043190
7	ilvA CDS	-3.316711233	0.0007243019622	WP_000216853.1	cellular amino acid biosynthetic process [GO:0008652]; isoleucine biosynthetic process [GO:0009097]; threonine metabolic process [GO:0006566]	cellular amino acid biosynthetic process [GO:0008652]; isoleucine biosynthetic process [GO:0009097]; threonine metabolic process [GO:0006566]	L-threonine ammonia-lyase activity [GO:0004794]; pyridoxal phosphate binding [GO:0030170]; cellular amino acid biosynthetic process [GO:0008652]; isoleucine biosynthetic process [GO:0009097]; threonine metabolic process [GO:0006566]	GO:0004794; GO:0006566; GO:0008652; GO:0009097; GO:0030170
8	ATP phosphoribosyltransferase regulatory subunit CDS	-3.200985502	1.41E-06	WP_001065590.1	histidine biosynthetic process [GO:0000105]	histidine biosynthetic process [GO:0000105]	cytoplasm [GO:0005737]; histidine biosynthetic process [GO:0000105]	GO:0000105; GO:0005737
9	phosphate/phosphite/phosphonate ABC transporter substrate-binding protein CDS	-3.083127957	6.03E-05	WP_000787672.1	transmembrane transport [GO:0055085]	transmembrane transport [GO:0055085]	ATP-binding cassette (ABC) transporter complex [GO:0043190]; transmembrane transport [GO:0055085]	GO:0043190; GO:0055085
10	sak CDS	-3.026639031	1.43E-12	WP_000919350.1	pathogenesis [GO:0009405]; plasminogen activation [GO:0031639]	pathogenesis [GO:0009405]; plasminogen activation [GO:0031639]	extracellular region [GO:0005576]; pathogenesis [GO:0009405]; plasminogen activation [GO:0031639]	GO:0005576; GO:0009405; GO:0031639
11	phosphoribosylanthranilate isomerase CDS	-2.968566578	0.00165256602	WP_000768192.1	tryptophan biosynthetic process [GO:0000162]	tryptophan biosynthetic process [GO:0000162]	phosphoribosylanthranilate isomerase activity [GO:0004640]; tryptophan biosynthetic process [GO:0000162]	GO:0000162; GO:0004640
12	putative holin-like toxin CDS	-2.938401634	3.54E-13					
13	ABC transporter ATP-binding protein CDS	-2.931048588	2.69E-09	WP_001229911.1			plasma membrane [GO:0005886]; ATP binding [GO:0005524]	GO:0005524; GO:0005886
14	type I toxin-antitoxin system Fst family toxin CDS	-2.912751182	3.09E-07	WP_001791613.1			integral component of membrane [GO:0016021]	GO:0016021
15	putative holin-like toxin CDS	-2.908777827	9.99E-12	WP_011447039.1			integral component of membrane [GO:0016021]	GO:0016021
16	ATP phosphoribosyltransferase CDS	-2.866865588	0.001539483174	WP_000944150.1	histidine biosynthetic process [GO:0000105]	histidine biosynthetic process [GO:0000105]	cytoplasm [GO:0005737]; ATP binding [GO:0005524]; ATP phosphoribosyltransferase activity [GO:0003879]; histidine biosynthetic process [GO:0000105]	GO:0000105; GO:0003879; GO:0005524; GO:0005737
17	hypothetical protein CDS	-2.826285725	1.74E-09	WP_000691527.1				
18	type I toxin-antitoxin system Fst family toxin CDS	-2.791658451	6.38E-10	WP_000482650.1			integral component of membrane [GO:0016021]	GO:0016021
19	hypothetical protein CDS	-2.743611094	6.76E-12	WP_001802298.1			integral component of membrane [GO:0016021]	GO:0016021
20	gallidermin/nisin family lantibiotic CDS	-2.677590274	0.001287630754	WP_000416756.1	cytolysis [GO:0019835]; defense response to bacterium [GO:0042742]	cytolysis [GO:0019835]; defense response to bacterium [GO:0042742]	extracellular region [GO:0005576]; signaling receptor binding [GO:0005102]; cytolysis [GO:0019835]; defense response to bacterium [GO:0042742]	GO:0005102; GO:0005576; GO:0019835; GO:0042742
21	pepA1 CDS	-2.676126236	0.0003067409243	WP_001801861.1				
22	sasF CDS	-2.6677212	6.16E-08	WP_001151900.1			cell wall [GO:0005618]; extracellular region [GO:0005576]	GO:0005576; GO:0005618
23	competence protein ComK CDS	-2.633700294	3.72E-05	WP_000287265.1	establishment of competence for transformation [GO:0030420]	establishment of competence for transformation [GO:0030420]	establishment of competence for transformation [GO:0030420]	GO:0030420
24	mgrA CDS	-2.632616436	1.24E-18	WP_001283444.1	pathogenesis [GO:0009405]	pathogenesis [GO:0009405]	cytoplasm [GO:0005737]; DNA binding [GO:0003677]; DNA-binding transcription factor activity [GO:0003700]; pathogenesis [GO:0009405]	GO:0003677; GO:0003700; GO:0005737; GO:0009405
25	TM2 domain-containing protein CDS	-2.620009741	8.14E-10	WP_001198024.1			integral component of membrane [GO:0016021]	GO:0016021
26	NupC/NupG family nucleoside CNT transporter CDS	-2.61005245	5.10E-11	WP_001022961.1			integral component of membrane [GO:0016021]; nucleoside transmembrane transporter activity [GO:0005337]	GO:0005337; GO:0016021
27	lukG CDS	-2.602634958	1.41E-06	WP_000595324.1	cytolysis in other organism [GO:0051715]; pathogenesis [GO:0009405]	cytolysis in other organism [GO:0051715]; pathogenesis [GO:0009405]	extracellular region [GO:0005576]; cytolysis in other organism [GO:0051715]; pathogenesis [GO:0009405]	GO:0005576; GO:0009405; GO:0051715
28	hypothetical protein CDS	-2.59128668	0.0001804965816	WP_000499647.1			integral component of membrane [GO:0016021]	GO:0016021

Supplementary Table 10. Differentially Expressed Genes (DEGs) isolated from HG4 treated sample

29	argH CDS	-2.586332026	1.15E-05	WP_000066062.1	arginine biosynthetic process via ornithine [GO:0042450]	arginine biosynthetic process via ornithine [GO:0042450]	cytoplasm [GO:0005737]; argininosuccinate lyase activity [GO:0004056]; arginine biosynthetic process via ornithine [GO:0042450]	GO:0004056; GO:0005737; GO:0042450
30	sarR CDS	-2.574986576	4.92E-10	WP_000036076.1	pathogenesis [GO:0009405]; regulation of transcription, DNA-templated [GO:0006355]	pathogenesis [GO:0009405]; regulation of transcription, DNA-templated [GO:0006355]	cytoplasm [GO:0005737]; DNA binding [GO:0003677]; pathogenesis [GO:0009405]; regulation of transcription, DNA-templated [GO:0006355]	GO:0003677; GO:0005737; GO:0006355; GO:0009405
31	tnpA CDS	-2.566420112	1.74E-05					
32	transposase CDS	-2.525902981	0.008909283528					
33	ABC transporter permease CDS	-2.517682235	8.75E-05	WP_000761395.1			integral component of membrane [GO:0016021]; plasma membrane [GO:0005886]	GO:0005886; GO:0016021
34	replication initiation protein CDS	-2.510445444	0.003696310511					
35	AI-2E family transporter CDS	-2.505660602	0.0002829887154	WP_000933105.1			integral component of membrane [GO:0016021]	GO:0016021
36	hypothetical protein CDS	-2.502720071	5.81E-05	WP_000827000.1			plasma membrane [GO:0005886]	GO:0005886
37	arcC CDS	-2.45815054	6.83E-07	WP_001074342.1	arginine metabolic process [GO:0006525]; carbamoyl phosphate catabolic process [GO:0035975]	arginine metabolic process [GO:0006525]; carbamoyl phosphate catabolic process [GO:0035975]	cytoplasm [GO:0005737]; ATP binding [GO:0005524]; carbamate kinase activity [GO:0008804]; arginine metabolic process [GO:0006525]; carbamoyl phosphate catabolic process [GO:0035975]	GO:0005524; GO:0005737; GO:0006525; GO:0008804; GO:0035975
38	hypothetical protein CDS	-2.441636517	4.60E-08	WP_000991705.1				
39	uhpT CDS	-2.409652169	4.00E-07	WP_001008722.1			integral component of membrane [GO:0016021]; plasma membrane [GO:0005886]; transmembrane transporter activity [GO:0022857]	GO:0005886; GO:0016021; GO:0022857
40	ruvX CDS	-2.401625335	3.31E-13	WP_000939059.1	rRNA 5'-end processing [GO:0000967]	rRNA 5'-end processing [GO:0000967]	cytoplasm [GO:0005737]; nuclease activity [GO:0004518]; rRNA 5'-end processing [GO:0000967]	GO:0000967; GO:0004518; GO:0005737
41	hypothetical protein CDS	-2.390525989	2.70E-07	WP_000735558.1				
42	SarA family transcriptional regulator CDS	-2.384171227	8.81E-08	WP_000032855.1	regulation of transcription, DNA-templated [GO:0006355]	regulation of transcription, DNA-templated [GO:0006355]	regulation of transcription, DNA-templated [GO:0006355]	GO:0006355
43	DUF961 family protein CDS	-2.381753003	0.004426857839	WP_000805733.1				
44	hypothetical protein CDS	-2.381480195	3.30E-08	WP_000031108.1				
45	hypothetical protein CDS	-2.361224037	4.11E-05	WP_001789984.1			integral component of membrane [GO:0016021]	GO:0016021
46	argininosuccinate synthase CDS	-2.353314959	2.90E-05	WP_000660045.1	arginine biosynthetic process [GO:0006526]	arginine biosynthetic process [GO:0006526]	cytoplasm [GO:0005737]; argininosuccinate synthase activity [GO:0004055]; ATP binding [GO:0005524]; arginine biosynthetic process [GO:0006526]	GO:0004055; GO:0005524; GO:0005737; GO:0006526
47	raiA CDS	-2.344393429	1.37E-07	WP_000617735.1	primary metabolic process [GO:0044238]; regulation of translation [GO:0006417]	primary metabolic process [GO:0044238]; regulation of translation [GO:0006417]	cytoplasm [GO:0005737]; primary metabolic process [GO:0044238]; regulation of translation [GO:0006417]	GO:0005737; GO:0006417; GO:0044238
48	Y-family DNA polymerase CDS	-2.338922278	8.47E-06	WP_000283016.1	DNA repair [GO:0006281]	DNA repair [GO:0006281]	damaged DNA binding [GO:0003684]; DNA repair [GO:0006281]	GO:0003684; GO:0006281
49	ABC-2 transporter permease CDS	-2.338845541	0.01363448676	WP_000677714.1			integral component of membrane [GO:0016021]	GO:0016021
50	hypothetical protein CDS	-2.306626719	0.0005018130123	WP_001788321.1				
51	PH domain-containing protein CDS	-2.291674093	1.29E-11	WP_000599587.1				
52	hypothetical protein CDS	-2.28502047	0.01922177786	WP_000431307.1			integral component of membrane [GO:0016021]	GO:0016021
53	histidinol dehydrogenase CDS	-2.27652191	0.004117944595	WP_000930643.1	histidine biosynthetic process [GO:0000105]	histidine biosynthetic process [GO:0000105]	histidinol dehydrogenase activity [GO:0004399]; NAD binding [GO:0051287]; zinc ion binding [GO:0008270]; histidine biosynthetic process [GO:0000105]	GO:0000105; GO:0004399; GO:0008270; GO:0051287
54	lantibiotic protection ABC transporter ATP-binding subunit CDS	-2.27513227	6.43E-05	WP_001096791.1			ATP binding [GO:0005524]; ATPase-coupled transmembrane transporter activity [GO:0042626]; hydrolase activity [GO:0016787]	GO:0005524; GO:0016787; GO:0042626
55	hypothetical protein CDS	-2.245011373	1.65E-09	WP_000801007.1				
56	phosphatidylinositol-specific phospholipase C CDS	-2.229518949	0.02065012141	WP_000710576.1	lipid catabolic process [GO:0016042]; pathogenesis [GO:0009405]; signal transduction [GO:0007165]	lipid catabolic process [GO:0016042]; pathogenesis [GO:0009405]; signal transduction [GO:0007165]	extracellular region [GO:0005576]; phosphatidylinositol diacylglycerol-lyase activity [GO:0004436]; phosphoric diester hydrolase activity [GO:0008081]; lipid catabolic process [GO:0016042]; pathogenesis [GO:0009405]; signal transduction [GO:0007165]	GO:0004436; GO:0005576; GO:0007165; GO:0008081; GO:0009405; GO:0016042
57	hypothetical protein CDS	-2.183541019	0.0005648907511	WP_001791821.1				
58	pseudouridine-5'-phosphate glycosidase CDS	-2.180076641	8.63E-08	WP_000002068.1	nucleobase catabolic process [GO:0046113]	nucleobase catabolic process [GO:0046113]	hydrolase activity, acting on glycosyl bonds [GO:0016798]; metal ion binding [GO:0046872]; pseudouridylate synthase activity [GO:0004730]; nucleobase catabolic process [GO:0046113]	GO:0004730; GO:0016798; GO:0046113; GO:0046872

Supplementary Table 10. Differentially Expressed Genes (DEGs) isolated from HG4 treated sample

59	glucosamine-6-phosphate isomerase CDS	-2.169047337	5.73E-06	WP_001251224.1	N-acetylglucosamine metabolic process [GO:0006044]	N-acetylglucosamine metabolic process [GO:0006044]	glucosamine-6-phosphate deaminase activity [GO:0004342]; N-acetylglucosamine metabolic process [GO:0006044]	GO:0004342; GO:0006044
60	sarA CDS	-2.166457168	6.87E-11	WP_001018677.1	pathogenesis [GO:0009405]; regulation of transcription, DNA-templated [GO:0006355]	pathogenesis [GO:0009405]; regulation of transcription, DNA-templated [GO:0006355]	cytoplasm [GO:0005737]; DNA binding [GO:0003677]; metal ion binding [GO:0046872]; pathogenesis [GO:0009405]; regulation of transcription, DNA-templated [GO:0006355]	GO:0003677; GO:0005737; GO:0006355; GO:0009405; GO:0046872
61	agrB CDS	-2.143560741	5.34E-06	WP_001105707.1	pathogenesis [GO:0009405]; quorum sensing [GO:0009372]	pathogenesis [GO:0009405]; quorum sensing [GO:0009372]	integral component of membrane [GO:0016021]; plasma membrane [GO:0005886]; peptidase activity [GO:0008233]; pathogenesis [GO:0009405]; quorum sensing [GO:0009372]	GO:0005886; GO:0008233; GO:0009372; GO:0009405; GO:0016021
62	graF CDS	-2.116551407	0.0006889745272	WP_000064214.1				
63	serS CDS	-2.108810827	5.55E-08	WP_000884334.1	selenocysteine biosynthetic process [GO:0016260]; selenocysteinyl-tRNA(Sec) biosynthetic process [GO:0097056]; seryl-tRNA aminoacylation [GO:0006434]	selenocysteine biosynthetic process [GO:0016260]; selenocysteinyl-tRNA (Sec) biosynthetic process [GO:0097056]; seryl-tRNA aminoacylation [GO:0006434]	cytoplasm [GO:0005737]; ATP binding [GO:0005524]; serine-tRNA ligase activity [GO:0004828]; selenocysteine biosynthetic process [GO:0016260]; selenocysteinyl-tRNA(Sec) biosynthetic process [GO:0097056]; seryl-tRNA aminoacylation [GO:0006434]	GO:0004828; GO:0005524; GO:0005737; GO:0006434; GO:0016260; GO:0097056
64	cyclic lactone autoinducer peptide CDS	-2.104876598	7.32E-06	WP_001093929.1				
65	adhP CDS	-2.100255645	2.54E-12	WP_001200748.1			alcohol dehydrogenase (NAD+) activity [GO:0004022]; zinc ion binding [GO:0008270]	GO:0004022; GO:0008270
66	S8 family serine peptidase CDS	-2.093774316	0.0007617405055	WP_000691541.1			serine-type endopeptidase activity [GO:0004252]	GO:0004252
67	ABC transporter permease CDS	-2.061586921	0.001147296609	WP_000569120.1	nickel cation transport [GO:0015675]; transmembrane transport [GO:0055085]	nickel cation transport [GO:0015675]; transmembrane transport [GO:0055085]	integral component of membrane [GO:0016021]; plasma membrane [GO:0005886]; nickel cation transport [GO:0015675]; transmembrane transport [GO:0055085]	GO:0005886; GO:0015675; GO:0016021; GO:0055085
68	hypothetical protein CDS	-2.060439022	7.24E-06	WP_000193917.1			integral component of membrane [GO:0016021]	GO:0016021
69	ABC transporter permease CDS	-2.058629482	4.41E-09	WP_000536961.1			integral component of membrane [GO:0016021]; ATP binding [GO:0005524]	GO:0005524; GO:0016021
70	mecA CDS	-2.056163287	3.70E-11	WP_001217730.1			protein-macromolecule adaptor activity [GO:0030674]	GO:0030674
71	lukH CDS	-2.044574218	0.003290643513	WP_000791407.1	cytolysis in other organism [GO:0051715]; pathogenesis [GO:0009405]	cytolysis in other organism [GO:0051715]; pathogenesis [GO:0009405]	extracellular region [GO:0005576]; cytolysis in other organism [GO:0051715]; pathogenesis [GO:0009405]	GO:0005576; GO:0009405; GO:0051715
72	ABC transporter permease CDS	-2.024168106	8.17E-06	WP_000398941.1	nickel cation transport [GO:0015675]; transmembrane transport [GO:0055085]	nickel cation transport [GO:0015675]; transmembrane transport [GO:0055085]	integral component of membrane [GO:0016021]; plasma membrane [GO:0005886]; nickel cation transport [GO:0015675]; transmembrane transport [GO:0055085]	GO:0005886; GO:0015675; GO:0016021; GO:0055085
73	MerR family transcriptional regulator CDS	-2.023803688	0.02699973889	WP_000850012.1	regulation of transcription, DNA-templated [GO:0006355]	regulation of transcription, DNA-templated [GO:0006355]	DNA binding [GO:0003677]; regulation of transcription, DNA-templated [GO:0006355]	GO:0003677; GO:0006355
74	qoxD CDS	-2.023044285	3.07E-08	WP_001797236.1	ATP synthesis coupled electron transport [GO:0042773]	ATP synthesis coupled electron transport [GO:0042773]	integral component of membrane [GO:0016021]; plasma membrane [GO:0005886]; oxidoreductase activity, acting on diphenols and related substances as donors, oxygen as acceptor [GO:0016682]; ATP synthesis coupled electron transport [GO:0042773]	GO:0005886; GO:0016021; GO:0016682; GO:0042773
75	cap8F CDS	-2.007558487	0.04059714434	WP_001028293.1			catalytic activity [GO:0003824]	GO:0003824
76	PTS glucose transporter subunit IIA CDS	-2.000161573	2.85E-11	WP_000473652.1	phosphoenolpyruvate-dependent sugar phosphotransferase system [GO:0009401]	phosphoenolpyruvate-dependent sugar phosphotransferase system [GO:0009401]	cytoplasm [GO:0005737]; kinase activity [GO:0016301]; metal ion binding [GO:0046872]; phosphoenolpyruvate-dependent sugar phosphotransferase system [GO:0009401]	GO:0005737; GO:0009401; GO:0016301; GO:0046872
77	DUF5079 family protein CDS	2.015974001	0.03985739591	WP_000208414.1			integral component of membrane [GO:0016021]	GO:0016021
78	phosphate ABC transporter substrate-binding protein PstS CDS	2.027093121	0.0388002751	WP_000759232.1	phosphate ion transport [GO:0006817]	phosphate ion transport [GO:0006817]	plasma membrane [GO:0005886]; phosphate ion binding [GO:0042301]; phosphate ion transport [GO:0006817]	GO:0005886; GO:0006817; GO:0042301
79	sdpB CDS	2.042149721	0.01953242817	WP_000426696.1	CAAX-box protein processing [GO:0071586]	CAAX-box protein processing [GO:0071586]	integral component of membrane [GO:0016021]; metalloendopeptidase activity [GO:0004222]; CAAX-box protein processing [GO:0071586]	GO:0004222; GO:0016021; GO:0071586
80	GNAT family N-acetyltransferase CDS	2.047190159	0.0208497551	WP_000620953.1			N-acetyltransferase activity [GO:0008080]	GO:0008080
81	hypothetical protein CDS	2.051976683	0.006912650185	WP_000414205.1				
82	typA CDS	2.124746689	8.86E-08	WP_000182654.1	ribosomal large subunit assembly [GO:0000027]	ribosomal large subunit assembly [GO:0000027]	cytoplasm [GO:0005737]; GTP binding [GO:0005525]; GTPase activity [GO:0003924]; ribosome binding [GO:0043022]; rRNA binding [GO:0019843]; tRNA binding [GO:0000049]; ribosomal large subunit assembly [GO:0000027]	GO:0000027; GO:0000049; GO:0003924; GO:0005525; GO:0005737; GO:0019843; GO:0043022
83	purK CDS	2.142987263	0.0008010238944	WP_001010412.1	'de novo' IMP biosynthetic process [GO:0006189]	'de novo' IMP biosynthetic process [GO:0006189]	5-(carboxyamino)imidazole ribonucleotide synthase activity [GO:0034028]; ATP binding [GO:0005524]; metal ion binding [GO:0046872]; NAD binding [GO:0051287]; phosphoribosylaminoimidazole carboxylase activity [GO:0004638]; 'de novo' IMP biosynthetic process [GO:0006189]	GO:0004638; GO:0005524; GO:0006189; GO:0034028; GO:0046872; GO:0051287

Supplementary Table 10. Differentially Expressed Genes (DEGs) isolated from HG4 treated sample

84	HAD family hydrolase CDS	2.167140209	0.0181436129	WP_000667097.1			phosphoglycolate phosphatase activity [GO:0008967]	GO:0008967
85	nreB CDS	2.176189733	1.31E-11	WP_000606546.1			cytoplasm [GO:0005737]; integral component of membrane [GO:0016021]; 4 iron, 4 sulfur cluster binding [GO:0051539]; ATP binding [GO:0005524]; iron ion binding [GO:0005506]; phosphorelay sensor kinase activity [GO:0000155]; protein dimerization activity [GO:0046983]	GO:0000155; GO:0005506; GO:0005524; GO:0005737; GO:0016021; GO:0046983; GO:0051539
86	CHAP domain-containing protein CDS	2.179781492	0.02583834357	WP_000247465.1			extracellular region [GO:0005576]; integral component of membrane [GO:0016021]; amidase activity [GO:0004040]	GO:0004040; GO:0005576; GO:0016021
87	DUF3169 family protein CDS	2.191384176	1.07E-07	WP_000698752.1			integral component of membrane [GO:0016021]; plasma membrane [GO:0005886]	GO:0005886; GO:0016021
88	nreC CDS	2.198075114	4.51E-12	WP_000706315.1	phosphorelay signal transduction system [GO:0000160]; regulation of transcription, DNA-templated [GO:0006355]	phosphorelay signal transduction system [GO:0000160]; regulation of transcription, DNA-templated [GO:0006355]	cytoplasm [GO:0005737]; DNA binding [GO:0003677]; phosphorelay signal transduction system [GO:0000160]; regulation of transcription, DNA-templated [GO:0006355]	GO:0000160; GO:0003677; GO:0005737; GO:0006355
89	serine hydrolase family protein CDS	2.201545199	0.001583956496	WP_000132890.1			hydrolase activity [GO:0016787]	GO:0016787
90	scb CDS	2.20332759	7.44E-07	WP_000669537.1			extracellular region [GO:0005576]	GO:0005576
91	DUF1398 family protein CDS	2.209809987	0.004793119298	WP_000886005.1				
92	isoprenylcysteine carboxyl methyltransferase family protein CDS	2.218468281	0.004601695576	WP_000603755.1			integral component of membrane [GO:0016021]; protein C-terminal S-isoprenylcysteine carboxyl O-methyltransferase activity [GO:0004671]	GO:0004671; GO:0016021
93	hypothetical protein CDS	2.23449313	0.02214341541	WP_001790169.1			integral component of membrane [GO:0016021]	GO:0016021
94	gatC CDS	2.277844105	0.0004266925159	WP_000170162.1	regulation of translational fidelity [GO:0006450]; translation [GO:0006412]	regulation of translational fidelity [GO:0006450]; translation [GO:0006412]	asparaginyl-tRNA synthase (glutamine-hydrolyzing) activity [GO:0050566]; ATP binding [GO:0005524]; glutaminyl-tRNA synthase (glutamine-hydrolyzing) activity [GO:0050567]; regulation of translational fidelity [GO:0006450]; translation [GO:0006412]	GO:0005524; GO:0006412; GO:0006450; GO:0050566; GO:0050567
95	tRNA1(Val) (adenine(37)-N6)-methyltransferase CDS	2.285901889	0.01277087653	WP_000910689.1			methyltransferase activity [GO:0008168]	GO:0008168
96	helix-turn-helix transcriptional regulator CDS	2.292071389	0.008159497935	WP_000429766.1			DNA binding [GO:0003677]	GO:0003677
97	PrsW family intramembrane metalloprotease CDS	2.309361475	0.0008379221639	WP_001789851.1			integral component of membrane [GO:0016021]; peptidase activity [GO:0008233]	GO:0008233; GO:0016021
98	hypothetical protein CDS	2.319210526	8.18E-07	WP_001251935.1				
99	sdrC CDS	2.36134001	0.01328147768	WP_001060483.1	cell adhesion [GO:0007155]	cell adhesion [GO:0007155]	cell wall [GO:0005618]; extracellular region [GO:0005576]; cell adhesion [GO:0007155]	GO:0005576; GO:0005618; GO:0007155
100	rhodanese-like domain-containing protein CDS	2.370158836	1.28E-05	WP_001276571.1			integral component of membrane [GO:0016021]	GO:0016021
101	GntR family transcriptional regulator CDS	2.385762344	0.003815964276	WP_000990133.1			DNA binding [GO:0003677]; DNA-binding transcription factor activity [GO:0003700]; transaminase activity [GO:0008483]	GO:0003677; GO:0003700; GO:0008483
102	protein-serine/threonine phosphatase Stp1 CDS	2.388954081	0.002320903601	WP_000888494.1			protein serine phosphatase activity [GO:0106306]; protein threonine phosphatase activity [GO:0106307]	GO:0106306; GO:0106307
103	ribonuclease III CDS	2.431330393	0.0003452988949	WP_000043237.1	mRNA processing [GO:0006397]; rRNA catabolic process [GO:0016075]; rRNA processing [GO:0006364]; tRNA processing [GO:0008033]	mRNA processing [GO:0006397]; rRNA catabolic process [GO:0016075]; rRNA processing [GO:0006364]; tRNA processing [GO:0008033]	cytoplasm [GO:0005737]; metal ion binding [GO:0046872]; ribonuclease III activity [GO:0004525]; rRNA binding [GO:0019843]; mRNA processing [GO:0006397]; rRNA catabolic process [GO:0016075]; rRNA processing [GO:0006364]; tRNA processing [GO:0008033]	GO:0004525; GO:0005737; GO:0006364; GO:0006397; GO:0008033; GO:0016075; GO:0019843; GO:0046872
104	tatC CDS	2.436923007	0.002386765691	WP_001795631.1	protein transport by the Tat complex [GO:0043953]	protein transport by the Tat complex [GO:0043953]	integral component of plasma membrane [GO:0005887]; TAT protein transport complex [GO:0033281]; protein transmembrane transporter activity [GO:0008320]; protein transport by the Tat complex [GO:0043953]	GO:0005887; GO:0008320; GO:0033281; GO:0043953
105	tRNA threonylcarbamoyladenosine dehydratase CDS	2.439279232	4.20E-07	WP_000682646.1			ubiquitin-like modifier activating enzyme activity [GO:0008641]	GO:0008641
106	superantigen-like protein SSL11 CDS	2.449426345	0.006482517158	WP_000769163.1	pathogenesis [GO:0009405]	pathogenesis [GO:0009405]	extracellular region [GO:0005576]; pathogenesis [GO:0009405]	GO:0005576; GO:0009405
107	recX CDS	2.46904785	0.0004190525296	WP_001124419.1	regulation of DNA repair [GO:0006282]	regulation of DNA repair [GO:0006282]	cytoplasm [GO:0005737]; regulation of DNA repair [GO:0006282]	GO:0005737; GO:0006282
108	penicillin-binding protein CDS	2.475568729	0.002734099117	WP_000184370.1			integral component of membrane [GO:0016021]	GO:0016021
109	thymidine kinase CDS	2.493362302	4.27E-05	WP_000273356.1	DNA biosynthetic process [GO:0071897]	DNA biosynthetic process [GO:0071897]	cytoplasm [GO:0005737]; ATP binding [GO:0005524]; thymidine kinase activity [GO:0004797]; zinc ion binding [GO:0008270]; DNA biosynthetic process [GO:0071897]	GO:0004797; GO:0005524; GO:0005737; GO:0008270; GO:0071897

110	efb CDS	2.539718971	3.60E-10	WP_000791587.1	pathogenesis [GO:0009405]	pathogenesis [GO:0009405]	extracellular space [GO:0005615]; complement binding [GO: 0001848]; pathogenesis [GO:0009405]	GO:0001848; GO:0005615; GO: 0009405
111	hypothetical protein CDS	2.554894233	0.008206586427	WP_000678764.1			integral component of membrane [GO:0016021]	GO:0016021
112	SH3 domain-containing protein CDS	2.573913241	2.19E-06					
113	pmtA CDS	2.581059061	1.62E-08	WP_000991302.1			ATP binding [GO:0005524]; hydrolase activity [GO:0016787]	GO:0005524; GO:0016787
114	BglG family transcription antiterminator CDS	2.602911254	6.23E-05	WP_000954261.1	phosphoenolpyruvate-dependent sugar phosphotransferase system [GO:0009401]; regulation of transcription, DNA-templated [GO:0006355]	phosphoenolpyruvate-dependent sugar phosphotransferase system [GO: 0009401]; regulation of transcription, DNA-templated [GO:0006355]	protein-N(PI)-phosphohistidine-sugar phosphotransferase activity [GO:0008982]; phosphoenolpyruvate-dependent sugar phosphotransferase system [GO:0009401]; regulation of transcription, DNA-templated [GO:0006355]	GO:0006355; GO:0008982; GO: 0009401
115	vraD CDS	2.691153034	0.00507866329	WP_000154162.1			ATP binding [GO:0005524]	GO:0005524
116	brnQ CDS	2.728120892	0.0008686188876	WP_001039275.1			integral component of membrane [GO:0016021]; plasma membrane [GO:0005886]; branched-chain amino acid transmembrane transporter activity [GO:0015658]	GO:0005886; GO:0015658; GO: 0016021
117	threonine/serine exporter family protein CDS	2.774399982	0.003813860213	WP_000498572.1			integral component of membrane [GO:0016021]	GO:0016021
118	teichoic acid D-Ala incorporation-associated protein DltX CDS	2.823125899	5.90E-05	WP_000837752.1			integral component of membrane [GO:0016021]	GO:0016021
119	LLM class flavin-dependent oxidoreductase CDS	2.836091081	0.002957806242	WP_000240058.1			(R)-limonene 1,2-monooxygenase NADH activity [GO:0106319]; (R)-limonene 1,2-monooxygenase NADPH activity [GO:0106320]; (S)-limonene 1,2-monooxygenase activity [GO:0052601]	GO:0052601; GO:0106319; GO: 0106320
120	coa CDS	2.870667971	8.17E-09	WP_000744073.1				
121	rsmA CDS	2.953205492	0.0001852488753	WP_000886500.1			cytoplasm [GO:0005737]; 16S rRNA (adenine(1518)-N(6)/adenine (1519)-N(6))-dimethyltransferase activity [GO:0052908]; RNA binding [GO:0003723]	GO:0003723; GO:0005737; GO: 0052908
122	GntR family transcriptional regulator CDS	3.443297153	1.81E-07	WP_000922329.1			DNA binding [GO:0003677]; DNA-binding transcription factor activity [GO:0003700]; transaminase activity [GO:0008483]	GO:0003677; GO:0003700; GO: 0008483
123	ribonuclease HIII CDS	3.560207505	9.94E-05	WP_001284258.1	RNA catabolic process [GO:0006401]	RNA catabolic process [GO:0006401]	cytoplasm [GO:0005737]; magnesium ion binding [GO:0000287]; RNA binding [GO:0003723]; RNA-DNA hybrid ribonuclease activity [GO:0004523]; RNA catabolic process [GO:0006401]	GO:0000287; GO:0003723; GO: 0004523; GO:0005737; GO: 0006401

Supplementary Table 11. Frequencies of Top 10 statistically significant Gene Ontology (GO) ID and description in the Differentially expressed genes (DEGs) in the HG4 treatment group

GO ID	Count	Percentage Occurrence in DEGs	Term Descriptions
**GO:0016021**	30	11%	integral component of membrane
**GO:0005737**	19	7%	cytoplasm
**GO:0005524**	14	5%	ATP binding
**GO:0005576**	10	4%	extracellular region
**GO:0005886**	10	4%	plasma membrane
**GO:0009405**	10	4%	pathogenesis
**GO:0003677**	7	3%	DNA binding
**GO:0006355**	6	2%	regulation of transcription, DNA-templated
**GO:0046872**	4	1%	metal ion binding
**GO:0008270**	3	1%	zinc ion binding
